# 40 Years of Duocarmycins: A Graphical Structure/Function Review of Their Chemical Evolution, from SAR to Prodrugs and ADCs

**DOI:** 10.1021/jacsau.2c00448

**Published:** 2022-11-15

**Authors:** Jan G. Felber, Oliver Thorn-Seshold

**Affiliations:** Department of Pharmacy, Ludwig-Maximilians University of Munich; Butenandtstr. 5-13, D-81377 Munich, Germany

**Keywords:** duocarmycin, cancer prodrug, CC-1065, antibody-drug-conjugates (ADC), CBI therapeutics, structural evolution

## Abstract

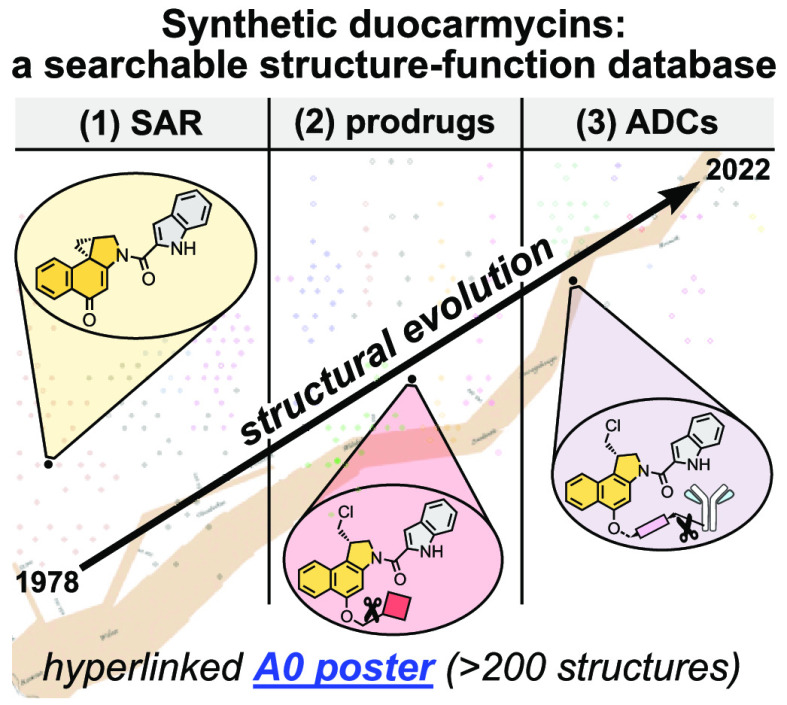

Synthetic analogues of the DNA-alkylating cytotoxins of the duocarmycin class have been extensively investigated in the past 40 years, driven by their high potency, their unusual mechanism of bioactivity, and the beautiful modularity of their structure–activity relationship (SAR). This Perspective analyzes how the molecular designs of synthetic duocarmycins have evolved: from (1) early SAR studies, through to modern applications for directed cancer therapy as (2) prodrugs and (3) antibody–drug conjugates in late-stage clinical development. Analyzing 583 primary research articles and patents from 1978 to 2022, we distill out a searchable A0-format “Minard map” poster of ca. 200 key structure/function-tuning steps tracing chemical developments across these three key areas. This structure-based overview showcases the ingenious approaches to tune and target bioactivity, that continue to drive development of the elegant and powerful duocarmycin platform.

## 1 Introduction

The natural products CC-1065 and duocarmycin SA are irreversible DNA alkylators that react after docking in the minor groove. Since their isolation from *Streptomyces* from 1978 onward,^1,2^ their picomolar cytotoxic potency has attracted continuous attention. Several total syntheses have been reported,^3−5^ and biochemical research has shown how their site-selectivity of DNA alkylation depends on structural features and stereochemistry.^6,7^ Clinical drug^8^ and prodrug^9^ candidates for cancer treatment quickly advanced to phase I and II clinical trials.^10−13^ Even after initial trials were discontinued due to narrow therapeutic index or strong side effects, an entire “duocarmycin family” of synthetic analogues with a broad range of aims and applications have been pursued. This minireview aims to distill this diversity of duocarmycin development into a rapidly grasped, yet comprehensive, format.

**Figure 1:**
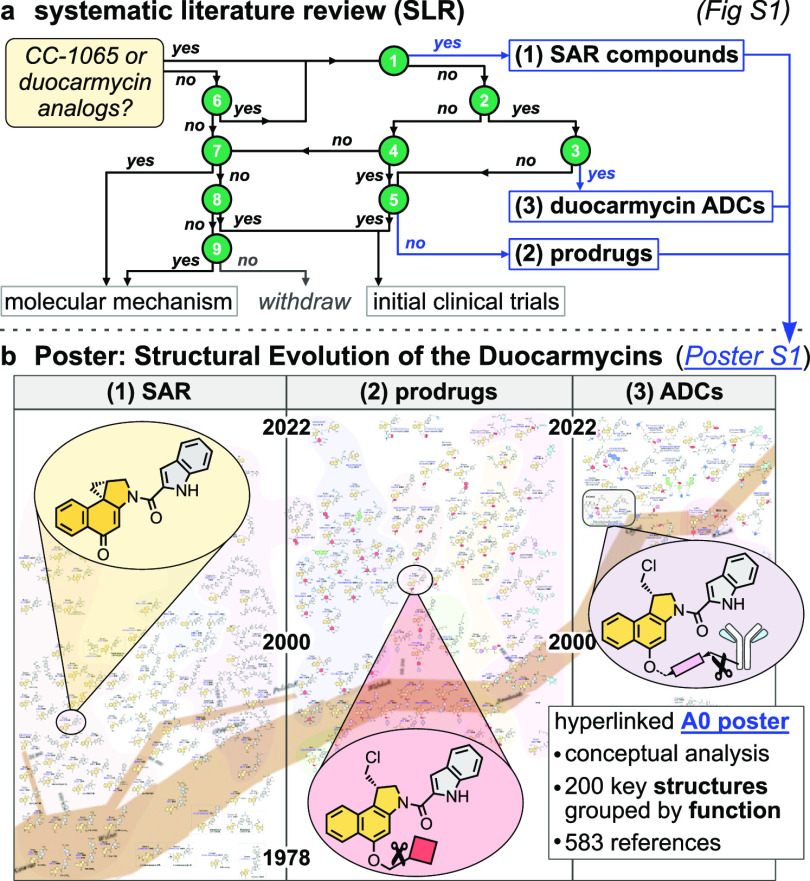
(a) 583 duocarmycin research reports were classified by a nine-point scheme, then structurally analyzed. (b) The A0-sized Poster S1 “Minard map” summarizes the structural evolution of >200 duocarmycin-derived agents from SAR to prodrugs and ADC.

Medicinal chemistry around duocarmycins has focused on three key areas (Figure 1). **(1) SAR** studies have explored the relationship of pharmacophore structure to DNA alkylation, and simplified synthetic analogues such as the cyclopropabenz[*e*]indoles (CBIs)^14^ have been developed, to retain the parent functionality but with greater chemical tractability.^15^**(2) Prodrugs** aiming to direct activity better toward target cancer cells have explored activatable alkylation motifs and bifunctional conjugates.^16^**(3) Antibody-drug conjugates** (ADCs) have also been developed for improved targeting, and in this incarnation the first duocarmycin derivative was recently FDA-approved.^17^

In this Perspective we present a focused digest of the chemistry in these areas, collating most of the prolific research on duocarmycins using a systematic literature review workflow (SLR;^18^Figure 1a, details in Supporting Information), then graphically summarizing it for rapid analysis. We classify the SLR database according to research focus, use it for meta-analysis, and provide it for future researchers with an interest in the field to orient their molecular designs. We then provide a searchable, dynamic datafile in A0 poster format (Figure 1b; Poster S1) which groups and analyzes structural design features, with particular focus on duocarmycin family (1) SAR, (2) prodrugs and bifunctional conjugates, and (3) ADCs.

## 2 Systematic Literature Review (SLR)

SLR^18^ was conducted to collate and group the vast majority of experimental literature concerning duocarmycins. Two groups with low structural diversity were split off: (a) reports of the isolation, characterization, and mechanism of action of natural products structurally related to CC-1065; and (b) reports of preclinical and clinical trials of early cancer drug candidates. Three groups with high structural diversity are analyzed here: **(1) SAR**: synthesis and cellular evaluation of derivatives in structure–activity-relationship (SAR) studies; **(2) Prodrugs**: synthesis, evaluation, and/or therapeutic use of prodrugs, mainly based on bioactivation of the *seco*-duocarmycin latent alkylator functional unit, or of bifunctional small molecule conjugates bearing at least one (*seco*-)duocarmycin; **(3) ADCs**: synthesis, conjugation, and therapeutic efficacy of (multi)functional ADCs incorporating a synthetic duocarmycin or its *seco*-precursor.

**Figure 2:**
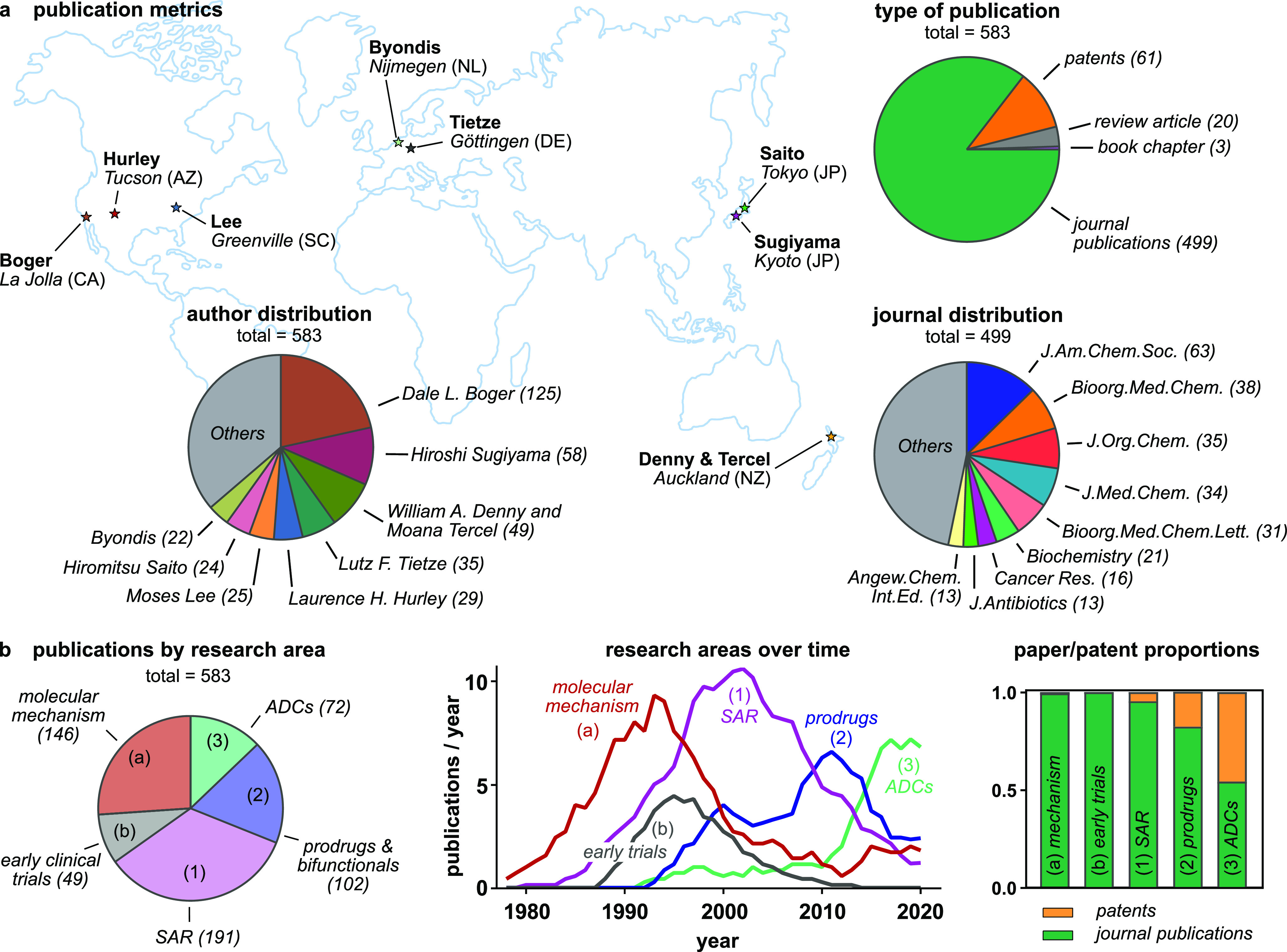
Literature metrics. (a) 583 primary research items form the duocarmycin literature database reviewed here. Charts show the major research groups (>20 publications) and journals (>10 publications). (b) The literature was grouped as: (**a**) natural products, biochemistry, and molecular mechanism of CC-1065 and close analogues; (**b**) initial clinical trial compounds; then the focus groups of this Perspective: (**1**) synthetic analogues and SAR; (**2**) prodrugs and bifunctional conjugates; (**3**) ADCs. Group histograms reveal the chronological progress of duocarmycin research. Paper/patent ratios may indicate perceived commercialisation chances.

Literature screening was first done by Boolean keyword search initiated with, e.g., [“CC-1065” or “duocarmycin”] AND [“analog” or “prodrug” or “derivative”] then refined with more specific keywords (see Supporting Information). From this, the major academic groups or pharmaceutical companies in each area of research were identified. For each group, all references reporting duocarmycin family agents were manually collected and categorized. Lastly, selected recent reviews on specific topics within the field of duocarmycins^16,17,19−24^ were harvested for additional references. Thus, a comprehensive duocarmycin structural library was assembled, from 583 reports—mainly of primary research (Figure 2).

### 2.1 Literature Metrics

A bibliographic overview of this library is given in Figure 2. Of the 583 total research items, the vast majority were published in scientific journals (123 journals, 499 publications, 86%) covering all areas from basic biology, biochemistry, medicinal chemistry, and molecular sciences, to physical chemistry, theoretical chemistry, and preclinical or clinical oncology. Major progress in chemical design and SAR has been published in chemistry (JACS 63, JOC 35, JMC 33, ANIE 13, Chem. Eur. J. 11) and bioorganic chemistry journals (BMC 38, BMCL 31); isolation and mechanism reports cluster in Biochemistry (21) and J. Antibiotics (13); and clinical results in oncology journals (Cancer Res. 16, Cancer Chemother. Pharmacol. 11, Mol. Cancer Ther. 9). 61 patents or patent applications filed by academic groups and pharmaceutical companies also entered this library (Figure 2a).

### 2.2 Evolution of the Focus of Duocarmycin Research

The sequence of duocarmycin development is easily visible when analyzing the five report groups by date (Figure 2b). Isolation and early molecular mechanism studies (group a; 146 items) dominate the 1980s and 1990s and have been key for further molecular designs. Rapidly following initial cytotoxicity studies, small molecule drugs (adozelesin and bizelesin) and hydrolytic prodrugs (carzelesin and pibrozelesin) were taken into initial clinical anticancer trials, that were discontinued during the 2000s (group b; 49 items). Hurley (29), Krueger (17), and others were the major academic groups driving both these developments.

Exhaustive and creative structural variations during the 1990s and 2000s largely mapped the SAR in this molecular class (**Group 1, SAR:** 192 items) with major contributions by Boger (125), Sugiyama (58), and Lee (25). Innovation increasingly focused on targeting, with activatable prodrugs and bifunctional small molecule conjugates taking off during the 2000s and 2010s (**Group 2, Prodrugs:** 102 items) led by Denny and Tercel (49), Tietze (35), Saito (23), and others. Finally, since the 2010s, conjugates of duocarmycins with monoclonal antibodies (**Group 3, ADCs:** 71 items) have opened up a new future for this class of bioactives. Combining the tunable potency and molecular flexibility of the duocarmycins, with the potential for enriched delivery to cancers, has led to a new wave of duocarmycin antibody–drug conjugates in clinical trials, driven by Byondis B.V. (22), Medarex Inc. (7), and others. With their increasing therapeutic relevance, the share of patents in the last two areas of research is also significantly higher (Figure 2b).

## 3 Structural Evolution of Duocarmycin Analogues

**Figure 3:**
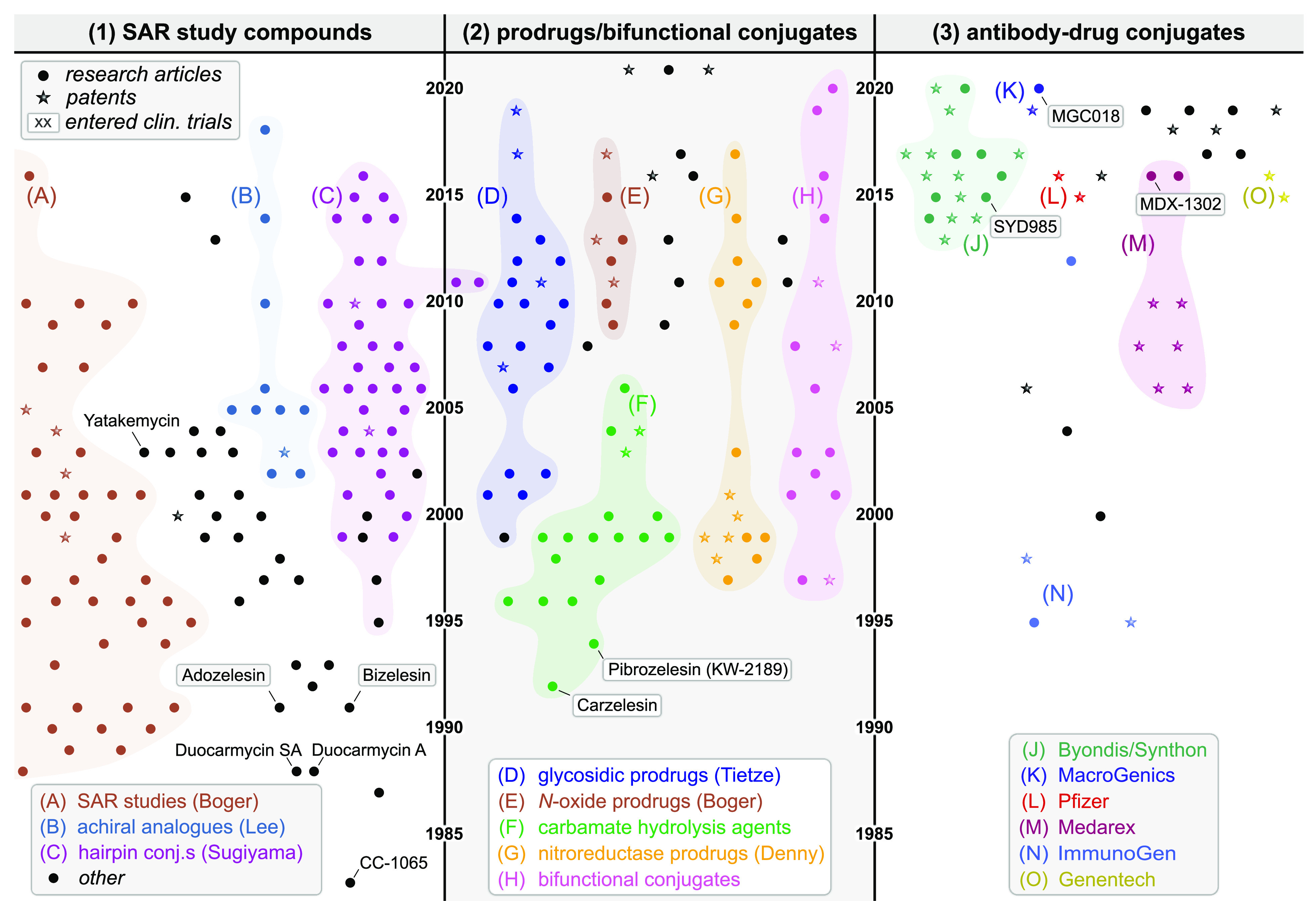
Structural developments of the duocarmycins (cartoon; all chemical structures in Poster S1). In Group 1 (SAR compounds), studies resolved the molecular motifs crucial for rational tuning of bioactivity. In Group 2 (Prodrugs), non-natural prodrugs (glycosides, nitroaryls, carbamates, *N*-oxides) and bifunctional conjugates expanded the scope of duocarmycins. In Group 3 (ADCs), industry has been a main driver of research.

The structural evolution of duocarmycins across these groups can also be best understood along a time axis, that resolves both the stepwise and disruptive innovations that have driven this field from 1978 to 2022. Figure 3 is a cartoon representation showing a data point for each research item in the three focus groups (circle: journal; star: patent); the A0-size Poster S1 in the Supporting Information maps these data points one-to-one onto representative chemical structures from each research item, color-coded for functionality, and DOI-hyperlinked for access to the original papers. We also encourage interested chemists to print a copy for easy reference.

### 3.1 Group 1: SAR Compounds

The lead natural product CC-1065 was isolated in 1978,^1^ and its first total synthesis was reported in 1988, laying the grounds for much synthetic development.^3^ During the 1990s and 2000s, systematic variations of both the core alkylator motif (“segment A”, typically an activated cyclopropane) and the DNA-docking motif (“segment B”) led to our current understanding of the structural features that need to be arranged for DNA association and sequence-selective alkylation (succinctly described by Hurley^7^).

**Figure 4:**
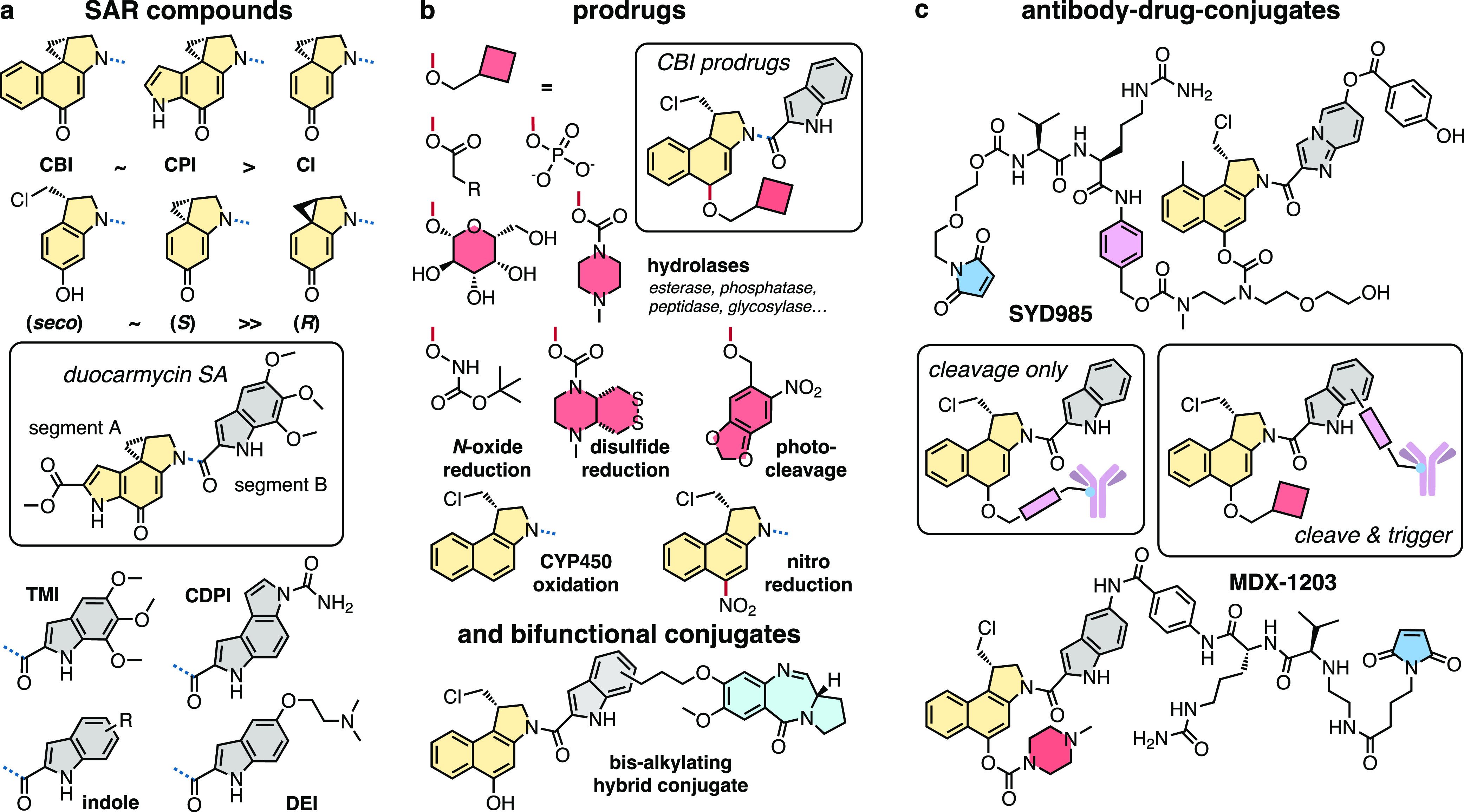
Structural elements of duocarmycin therapeutics. (a) SAR analysis: variations of segments A and B. (b) Activatable prodrugs: strategies to trigger bioactivity. (c) Antibody–drug conjugates: CBI-ADCs including clinical candidates SYD985 and MDX-1302. See also Poster S1.

Many heterocyclic systems beyond the native cyclopropa[*e*]pyrroloindole (CPI) of duocarmycin SA^25^ can serve as segment A. Much research has focused on the chemically tractable CBI, with optional substitutions;^14^ cyclopropaindole^26^ (CI) and others^27^ also alkylate DNA with the reactivity trend (CBI ∼ CPI > CI) (Figure 4a).

The activated cyclopropane electrophile must be in its native (*S*)-configuration for DNA alkylation,^28,29^ but high potency can be maintained with “proagent” *seco*-variants, that use *in situ* intramolecular Winstein spirocyclization to unfurl their activated cyclopropane, relying on the *para*-phenol.^30^ Good leaving groups (-Cl, -Br, -OMs)^9,31^ and several alternatives to the dihydroindole (5-, 6-, 7-membered rings)^32^ are all tolerated. Alternatively, masking this phenol suppresses spirocyclization:^33^ a disruptive step that opened the door for rational tuning of prodrug candidates in later years (see below). The group of Lee also introduced achiral *seco*-variants that are similarly reactive, but structurally simpler and more accessible.^34,35^

Segment B heterocycles have mainly clustered around indole-based rings that strengthen DNA binding. Stepwise simplification of the native dimeric segment B (in CC-1065) gave variously the deoxygenated CDPI dimer,^36^ 3,4,5-trimethoxyindole (TMI),^29^ and monoalkoxylated (DEI),^37^ or even simple mono/oligoindoles; and other heterocycles^38^ can also be used. These do impact DNA binding, alkylation site-selectivity, and potency; but overall, the tolerance for segment B variance is high (Figure 4a).

Assembling the A and B segments has also received attention. A remarkable class of hairpin duocarmycin conjugates was driven by Lown and Sugiyama in the 2000s.^39^ Using synthetic oligo-pyrroles/imidazoles from the minor-groove binder distamycin A as segment B binding domains gave potent duocarmycin analogues allowing sequence-selective alkylation in specific areas of DNA.^40,41^ “Standard” duocarmycins consist of segments A and B linked by an amide bond: but the natural product Yatakemycin^42,43^ has revealed that multiple B segments may be used, and randomly shuffled around without losing bioactivity.^44^ Dimeric bisalkylators with two A segments, allowing interstrand DNA cross-linking, also give extremely high potency.^45,46^

### 3.2 Group 2: Activatable Prodrugs and Bifunctionals

Early trials already exploited duocarmycin prodrugs where *seco*-duocarmycin bioactivity was to be triggered *in situ* by unmasking a *para-*phenol, to avoid parasitic loss of a preformed cyclopropane *en route* to target tissues. These carbamate hydrolysis designs (Carzelesin,^47,48^ Pibrozelesin/KW-2189^9,49^) were discontinued in clinical trials due to side effects and low therapeutic index.^13,50^ Follow-up work mined esters, solubilized carbamates, phosphates, and others as other hydrolytic activation methods (Figure 4b),^51−56^ although none of these promise any greater mechanistic selectivity for cancer, since the activating hydrolases are ubiquitously expressed.

Key steps toward cancer-selective prodrugs were initiated by the lab of Denny in the late 1990s. They introduced nitro-*seco*-CBIs that can be irreversibly reduced to the amino-*seco-*CBI in the low-oxygen conditions found in solid tumors. These amines then undergo Winstein cyclization becoming DNA-alkylators (Figure 4b).^57−59^ In the early 2000s, Tietze developed glycosidic prodrugs that can be built modularly, aiming at antitumor uses relying on glycosidases.^37,60,61^ Adopting novel chemistries in the 2010s, Boger introduced *O*-amino-*N*-acyl-*seco*-CBIs that are also subject to bioreductive activation.^62−64^ The field of masked *seco*-CBIs has by now exploited the full arsenal of chemical biology, passing through reducible Co-complexes,^65^ Fe(II)-reactive peroxides,^66^ photoactivated designs,^67,68^ and oxidizable naphthalenes.^69^ Recently, cyclic dichalcogenides (that resist monothiol exchange, but can be reductively activated by specific oxidorectases such as thioredoxin) have joined this panoply of prodrugs.^70,71^ Finally, bifunctional conjugates of duocarmycins with other pharmaceuticals (glucuronide,^72^ biotin,^73^ antibiotics,^74^ pyrrolobenzodiazepine (Figure 4b),^75,76^ albumin,^77^ peptides^78^) show the wide applicability and adaptability of this unique class of bioactives.

### 3.3 Group 3: Antibody–Drug Conjugates (ADCs)

Monoclonal antibodies against cancer-selective biomarkers have the potential to deliver high-potency cytotoxic cancer drugs to tumors in a targeted and therapeutically effective manner. The duocarmycins’ outstanding potency has motivated much ADC research, with two general designs emerging. Type A designs mask the *seco-*duocarmycin phenol with a linker conjugated to the antibody: allowing spirocyclization-based activation after linker cleavage. Intracellular cleavage of these linkers (dipeptides like ValCit that are prone to lysosomal proteolysis; hydrolyzable phosphates; reducible disulfides) can directly liberate the key Winstein cyclization phenol, but additional self-immolative spacers, that undergo cyclization or elimination cascades to liberate this phenol, are common.^79−83^ Type B designs attach a phenolic prodrug of the duocarmycin, via a peripheral site, to the antibody: permitting an extra layer of prodrug-based selectivity if prerelease activation can be avoided (Figure 4c). ADCs of Type B are less clearly reported, and many are IP-protected by pharmaceutical companies.^84−87^

**Figure 5:**
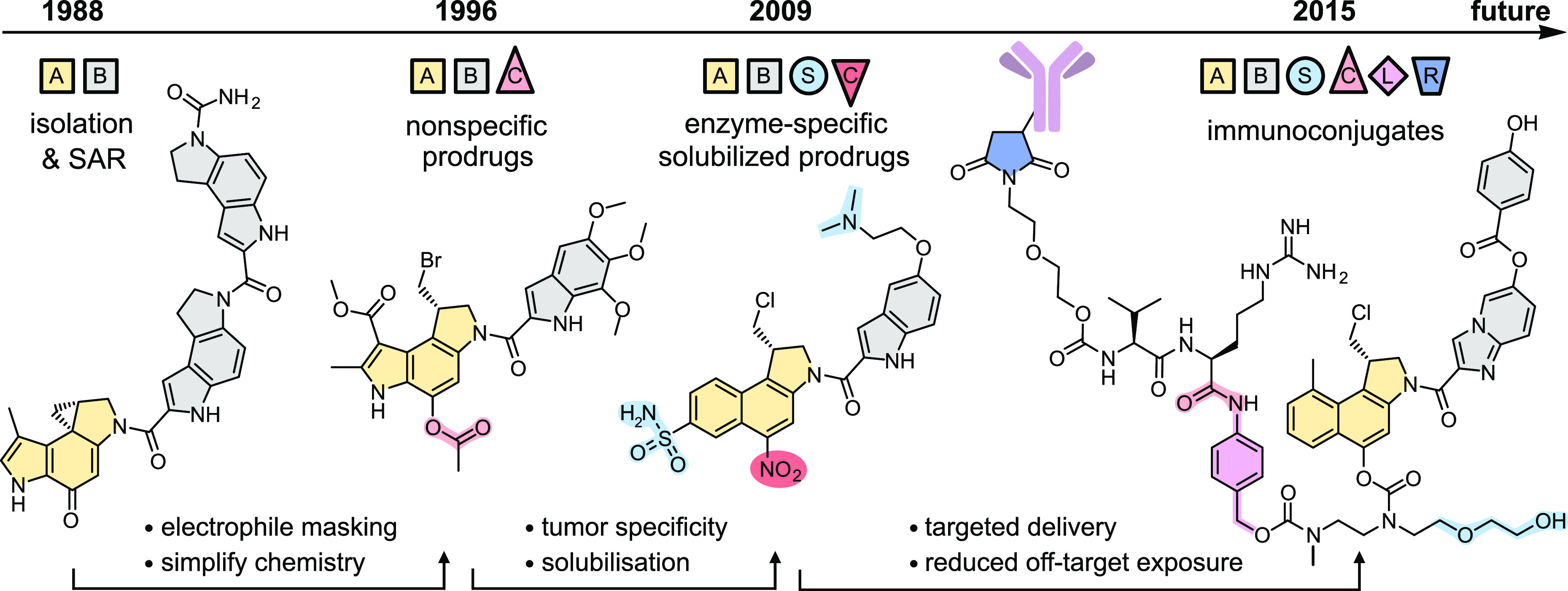
Color-coded highlights of the disruptive chemical steps that have led the duocarmycins from isolation to the clinic (see also Poster S1). (Key: A,B = A-,B-segments. C = intracellular cleavage site. S = solubilizer. L = self-immolative spacer. R = reactive group for antibody conjugation.)

The late-2000s rebirth of preclinical/clinical development in the duocarmycin class has essentially been driven by these ADCs, with a variety of designs achieving *in vivo* efficacy in mouse cancer models.^56,88−92^ Beyond the choice of biomarker and payload, ADC development must balance factors from conjugation site, degree of labeling, and linker nature,^93,94^ through to chemical conjugation method, making refinement of ADCs more complex than that of prodrugs.^95^ Nevertheless, the ADCs SYD985, MGC018, and MDX-1203 all reached clinical trials with promising results and high efficacy.^96−98^ While MDX-1203 was halted due to insufficient improvement of therapeutic benefit compared to alternative therapeutics, SYD985 was recently given fast-track approval as a follow-up or cotreatment for patients with HER2-positive metastatic breast cancer.^99^ This is the first duocarmycin approved for clinical use; its success will spur the developments of the future (Figure 5).

### 3.4 Guiding Principles for Future Developments

Predicting the future of drug development is a challenge, but this structure/function-based Perspective highlights trends that can drive the duocarmycins’ next decades. First, duocarmycins will remain high-value targets in cancer therapy. If their bioactivity can be directed, then their outstanding potency and their binding-triggered covalent-reactive mechanism, promise high efficacy with limited resistance in a broad scope of indications. Second, progress will continue to rely on disruptive innovations in duocarmycin chemistry. Key strategies so far include (i) SAR simplification for synthetic access, (ii) protecting the cyclopropane warhead by forming it *in situ*, (iii) chemical mechanisms for tumor-selective prodrug activation, and (iv) antibody-based mechanisms for tumor-selective prodrug delivery. Solubilization and self-immolative spacers have also proven critical. It is perhaps no accident that the first duocarmycin to be clinically successful had built in nearly all these strategies (Figure 5). We see much potential for new therapeutics that also harness these strategies but tackle other indications with different target expression profiles and biodistribution needs. We also believe that finding sufficiently selective yet sufficiently high-turnover chemical mechanisms for tumor-specific activation would revolutionize both ADC and small molecule prodrug applications, and we await developments in this still-underexplored chemical space.

## 4 Conclusions

Duocarmycins have undergone great efforts toward developing targeted cancer therapeutics. A careful understanding of their unusual mechanism of bioactivity, leveraging spirocyclization and docking for high-potency site-selective DNA alkylation, has enabled many creative approaches using the duocarmycins as a modular bioactive platform. Here we have provided a structured literature review tracking the chemical developments of the last 40 years, that have led from isolation to basic understanding, early trials and setbacks, re-engineering, and ultimately a first clinical anticancer agent.

We hope this concise overview will promote a structure/function-based understanding, allowing rational design and use of duocarmycin-based bioactives. It also follows the didactic tradition of Njardarson’s Posters^100^ with the A0-size Poster S1, that can be printed and hung up in hallways for graphical overview and discussions, or used digitally for easy followup of its 200 embedded key structures (DOI hyperlinks).

The modularity of duocarmycin bioactivity should encourage researchers to design in structural features *à la carte*. A structure-based overview to guide the choice and understanding of these features, with easy direction to the corresponding references, may be very helpful for gaining a *coup d’oeil* when entering new scientific territory: particularly where the frontiers of research are increasingly interdisciplinary. We can still expect much from the duocarmycins; and we hope this Perspective and its Poster bring a graphic understanding of how to design, incorporate, and exploit this powerful molecular class.

## Supporting Information Available

Details of how the SLR has been performed, including grouped lists of the major contributors to SAR/prodrug/ADC research, and containing the hyperlinked full-format bibliography of the 583 references in the SLR, organized by theme (PDF)
Poster S1 (>200 structures with hyperlinked references) (PDF)
ChemDraw file corresponding to the Poster, which contains all the duocarmycin structures without hyperlinks in CDX format for use/reuse of structures by copy-paste, or for mining the structures by SMILES (CDX)
∼600-work literature citation database provided as an RIS that can be imported into any reference manager software with one double-click, to make all the duocarmycin citations available (ZIP)

## Supplementary Material

au2c00448_si_002.pdf

au2c00448_si_003.cdx

## Abbreviations

CBI: cyclopropabenz[*e*]indole
CDPI: 3-carbamoyl-1,2-dihydro-3*H*-pyrrolo[3,2-*e*]indole-7-carboxylate
CPI: cyclopropa[*e*]pyrroloindole
CI: cyclopropaindole
DEI: *N*,*N*-dimethylaminoethoxyindole
TMI: 3,4,5-trimethoxyindole
