# Correction to “40 Years of Duocarmycins: A Graphical Structure/Function Review of Their Chemical Evolution, from SAR to Prodrugs and ADCs”

**DOI:** 10.1021/jacsau.3c00137

**Published:** 2023-04-19

**Authors:** Jan G. Felber, Oliver Thorn-Seshold

In the Introduction and in section 3.3 of the original article, we mistakenly referred to the ADC SYD985 as having been recently FDA-approved. Instead, we should have specified that it was recently granted FDA Fast Track designation (after positive results from a Phase I study with pretreated last-line HER2-positive metastatic breast cancer patients; SYD985.001/NCT02277717; 2018);^1^ and that following positive results of a Phase III TULIP trial (SYD985.002/NCT03262935), Byondis BV had filed the Biologics License Application (BLA) for the use of SYD985 in patients with HER2-positive unresectable locally advanced or metastatic breast cancer at the FDA in July 2022, with an expected decision date at May 12, 2023 (PDUFA action date).^2^

All reported results, statistics, and conclusions of the paper remain unchanged.
