# Supplementary material for: 40 Years of Duocarmycins: A Graphical Structure/Function Review of Their Chemical Evolution, from SAR to Prodrugs and ADCs
Source: JACS Au. 2022 Nov 15;2(12):2636–44. doi: 10.1021/jacsau.2c00448 (PMC9795467; doi:10.1021/jacsau.2c00448)
Supplement: Supplementary file 1 — au2c00448_si_001.pdf [file au2c00448_si_001.pdf]

Supplementary Information to

40 years of duocarmycins: a graphical structure/function review of their chemical evolution, from SAR to prodrugs and ADCs

Jan G. Felber* and Oliver Thorn-Seshold*

Department of Pharmacy, Ludwig Maximilians University of Munich
Butenandtstr. 5-13, D-81377 Munich, Germany.
E-mail: jan.felber@cup.lmu.de, oliver.thorn-seshold@cup.lmu.de

1. Systematic literature review (SLR) – extended 2

2. List of main contributors and their co-authors (≥3 authorships) 3

3. Bibliography 4

(a) molecular mechanism (146 items – 145 journal publications, 1 patent) 4

(b) early clinical trials (49 journal publications) 9

(1) SAR studies (191 items - 182 journal publications, 9 patents) 12

(2) prodrugs/bifunctionals (102 items - 84 journal publications, 18 patents) 20

(3) duocarmycin ADCs (72 items - 39 journal publications, 33 patents) 24

(x) duocarmycin reviews (23 items) 28

*The accompanying hyperlinked **A0 Poster** of the 200 Duocarmycins is actively curated; the current version is downloadable from the authors' website. Please inform us of any errors with the Poster. We are happy to acknowledge substantial suggestions by a cloth print of the updated poster, valued at 2.56 San Serriffe dollars.*

1. Systematic literature review (SLR) – extended

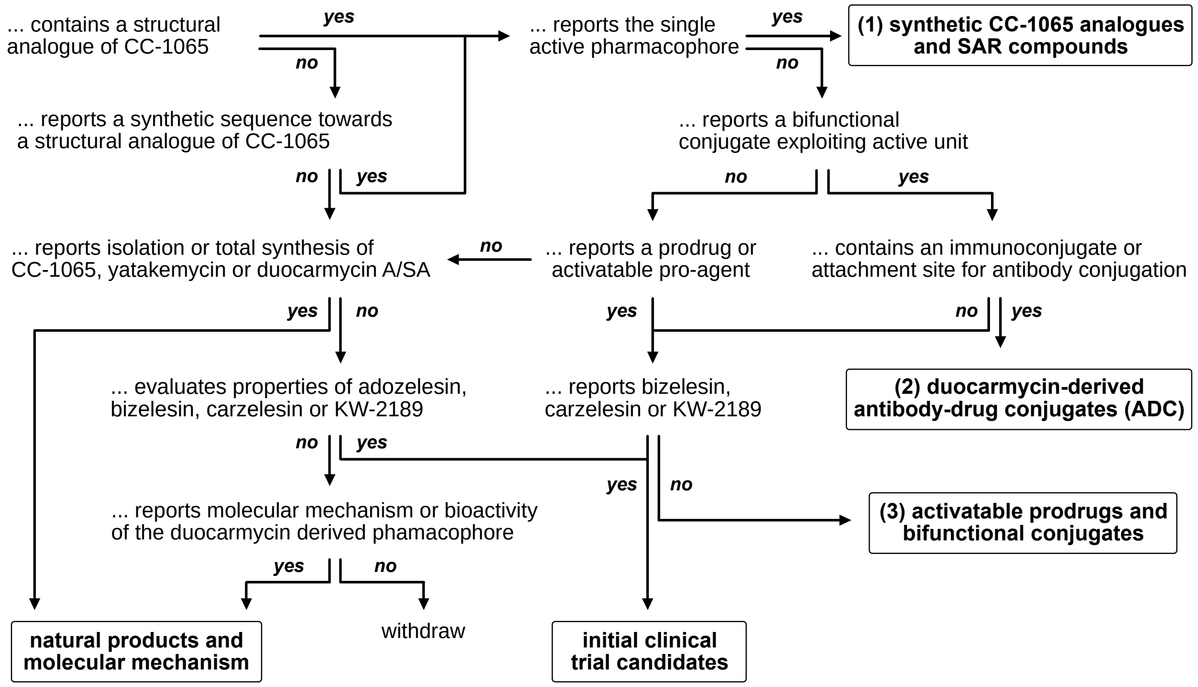

Supplementary Figure S1 Strategy and methods applied to collect and sort primary research items referring to duocarmycins according to systematic literature review (SLR).

The goal of this literature search was to collate the vast majority of references, that (a) report the isolation, molecular mechanism and use of natural products that structurally relate to CC-1065, that (b) report the results of early clinical trial compounds or their synthesis, evaluation or clinical results, that (1) report syntheses and/or evaluation of bioactivity of synthetic CC 1065 analogues or their respective seco-analogues, that (2) report synthesis, evaluation of bioactivity and/or therapeutic use in vivo of activatable proagents or bifunctional conjugates employing a synthetic CC 1065 analogue or its seco-version or that (3) report conjugation/assembly and/or therapeutic efficacy of (multi)functional antibody-drug conjugates incorporating a synthetic CC 1065 analogue or its seco version. This literature collection should be exploited as a database that allows a systematic analysis of both primary reports and metadata to decipher the chemical evolution of structure that the duocarmycin/CC-1065 derived alkylating unit has undergone over the last 40 years.

The search for this systematic literature review (SLR) has gone through many cycles using PubMed, ResearchGate, Google Scholar and Google for journal publications and review articles and FreePatentsOnline and Google Patents for related patents. During a first phase the phrases [“CC 1065” or “duocarmycin”] in combination with [“analogue” or “prodrug” or “derivative” or “review”] have been used and related references have been categorised according to (a1) isolation, early reports or total syntheses of CC-1065 related natural product and (a2) mechanism of action; (b) early small molecule based clinical trial compounds and related articles; (1a) synthetic derivatives that allow to conclude on structure-activity-relationship (SAR) and (1b) reports of synthons that allow are relevant for synthetic duocarmycin-analogues; (2a) functional proagents based on the seco-duocarmycin/cyclobenz[e]indole (CBI) functional unit or (2b) bifunctional conjugates that bear at least one duocarmycin analogue or its seco-version or (3) antibody-drug conjugates that employ a duocarmycin-type alkylating unit as pharmacophore for therapeutic use. More specifically, reports have been collected that refer to the natural products “duocarmycin A”, “duocarmycin SA”, “yatakemycin”, the specific clinical trial compounds “adozelesin”, “bizelesin”, “carzelesin” or “pibrozelesin/KW 2189”, the ADC-related research items [“duocarmyin” or “CBI” or “CPI”] in combination with [“ADC” or “antibody-drug conjugate”] and the clinical trial candidates “SYD985”, “MDX-1302” or “MGC018”.

Secondly, the major driving scientific groups behind each area of research have been identified (L. H. Hurley, D. L. Boger, M. Lee, H. Sugiyama, L. F. Tietze, H. Saito and W. A. Denny/M. Tercel, Byondis BV). For each group all references that report aspects related to the duocarmycin-derived phamacophore have been collected and categorized accordingly. The major scientific research groups or pharmaceutical companies, that drive CBI-based ADCs recently, have been identified (Byondis B.V./Syntarga B.V., Medarex Inc., Genentech Inc., Immunogen Inc. and others) and journal publications have been searched at Google Scholar, whereas patents have been search at FreePatentsOnline and Google Patents. Lastly, recent review articles (Wang 2021, Pors 2020, Lee 2015, Tietze 2011, Pors 2011 and Tietze 2009) have been screened and references have been cross-compared to collate a conclusive library.

2. List of main contributors and their co-authors (≥3 authorships)

(1) Dale Boger: Prof. of Chemistry, The Scripps Research Institute (TSRI), La Jolla:
https://www.scripps.edu/boger/ - 125 Publications:

Paul S. Kitos (14), Douglas S. Johnson (12), Ishizaki Takayoshi (11), Inkyu Hwang (10), Hamideh Zarrinmayeh (10), Robert M. Garbaccio (9), Weiya Yun (9), Christopher W. Boyce (8), Karen MacMillan (8), Stephan A. Munk (8), Qing Jin (7), David B. Kastrinsky (7), James P. Lajiness (7), Jeffrey A. McKie (7), Hui Cai (6), Nianhe Han (6), Michael P. Hedrick (6), Oranart Suntornwat (6), Scott E. Wolkenberg (6), Robert S. Coleman (5), Jay P. Parrish (5), Mark Searcey (5), Mark S. Tichenor (5), George A. Vielhauer (5), Pier G. Baraldi (4), Takahide Nishi (4), Philippe Mesini (4), Thomas J. Rayl (4), William M. Robertson (4), Christine M. Tarby (4), John D. Trzupek (4), Amanda L. Wolfe (4), Bernd Bollinger (3), Kristopher E. Boyle (3), Katherine K. Duncan (3), Joel Goldberg (3), Donald L. Hertzog (3), Nikhil K. Parelkar (3), Winston C. Tse (3), Philip Turnbull (3), Scott J. Weir (3), Ronald Wysocki (3).

(2) Hiroshi Sugiyama: Prof. Chemical Biology, Kyoto University:
http://kuchem.kyoto-u.ac.jp/chembio/ - 58 Publications:

Toshikazu Bando (45), Ken-Ichi Shinohara (18), Masafumi Minoshima (14), Isao Saito (12), Shunta Sasaki (12), Gengo Kashiwaza (10), Kaori Hashiya (9), Akihiko Narita (9), Rhys Dylan Taylor (6), Tsuyoshi Fujiwara (5), Zhi-Fu Tao (5), Jun Fujimoto (4), Hiroki Nagase (4), Tatsuhiko Shimizu (4), Anandhakumar Chandran (3), Yusuke Kawamoto (3), Rina Maeda (3), Gadesh N. Pandian (3), Toshiki Takagaki (3).

(3) Willam A. Denny: Prof. Medicinal Chemistry, Auckland University and Dr. Moana Tercel, Senior Research Fellow, Auckland University:
https://unidirectory.auckland.ac.nz/profile/b-denny; /unidirectory.auckland.ac.nz/profile/m-tercel.
49 publications with authorship of either William A. Denny or Moana Tercel:

Moana Tercel (38), William A. Denny (35), William R. Wilson (24), Graham Atwell (15), Frederik B. Pruijn (15), Ralph J. Stevenson (10), Sarath H. D. Liyanage (8), Thomas H. Pillow (8), Shangjin Yang (7), Jane Botting (6), Ho H. Lee (6), Adam V. Patterson (6), Jared Milbank (6), Amir Ashoorzadeh (4), Sarah P. McManaway (4), Sunali Mehta (4), Robert F. Anderson (3), Michael P. Hay (3), Eileen Smith (3).

(4) Lutz Tietze: Prof. Organic and biomolecular Chemistry, University of Goettingen:
Laboratory Website: http://www.tietze.chemie.uni-goettingen.de/ewelcome.php - 35 Publications:

Ingrid Schuberth (17), Birgit Krewer (13), Felix Major (8), Kianga Schmuck (7), J. Marian von Hof (6), Heiko J. Schuster (6), Frauke Alves (4), Tim Feuerstein (4), Frank Haunert (4), Tobias Herzig (4), Michael Müller (4), Olaf Panknin (3), Stephan A. Sieber (3).

(5) Laurence H. Hurley: Prof. Medicinal Chemistry, Arizona - 28 publications:

Daekyu Sun (11), Chong-Soon Lee (5), Paul A. Aristoff (4), Hsiung Chin Lin (4), Vincent L. Reynolds (4), David H. Swenson (4), Donald R. Needham-VanDev (3), Patrick J. McGovren (3), Gary L. Petzold (3), William C. Krueger (1), Li H. Li (1).

Related researchers and/or groups with independent publications and at least 3 authorships:

William C. Krueger (17), David H. Swenson (13), Mark D. Prairie (12), Gary L. Petzold (12), Patrick J. McGovren (11), Bijoy K. Bhuyan (10), Robert C. Kelly (10), Li H. Li (10), Paul A. Aristoff (7), Martha A. Warpehoski (7), Thomas F. DeKoning (5), Tanya L. Wallace (4), Ilse Gebhard (3).

(6) Moses Lee, Furman University, Greenville/Georgia State University:
https://www.researchgate.net/scientific-contributions/Moses-Lee-38856864 - 25 Publications:

John A. Hartley (14), Konstantinos Kiakos (14), Stephen J. Hudson (7), Carlie A. Price (6), Kaitlin Summerville (6), Heather L. Handl (5), Bethany Purnell (5), Heather Townes (5), Tetsuji Asao (4), Phillip Bowen (4), Brian Lingerfelt (4), Pravil Patil (4), Atsushi Sato (4), Adrienne E. Scott (4), James Toth (4), Tiffany T. Howard (3), LuAnne McNulty (3).

(7) Hiromitsu Saito, Kyowa Hakko Kogyo Co Ltd., Tokyo and Pharm. Res. Laboratories, Shimotogari:
https://www.researchgate.net/scientific-contributions/Hiromitsu-Saito-20136372 - 23 publications:

Satoru Nagamura (19), Katsushige Gomi (14), Ejii Kobayashi (13), Akira Asai (10), Nobuyoshi Amishiro (7), Akihiko Okamoto (7), Masami Okabe (6), Yutaka Kanda (3).

(8) Synthon Biopharmaceuticals/Byondis B.V., Nijmegen: www.byondis.com - 22 publications:

Patrick H. Beusker (13), Franciscus M. H. de Groot (10), Rudy G. E. Coumans (8), Willem Dokter (8), Henri J. Sprijker (8), Albertus F. Joosten (7), Ronald C. Elgersma (6), Miranda van der Lee (6), Wiro M. P. B. Menge (6), David Egging (5), Marco Timmers (5), Peter Goedings (4), Patrick Groothuis (4), Tijl Huijbregts (4), Alessandro D. Santin (4), Ruud Ubink (4), Gijs Verheijden (4), Tanja van Achterberg (3), Masoud Azodi (3), Elena Bonazzoli (3), Stefania Bellone (3), Jonathan Black (3), Natalia Buza (3), Diels van den Dobbelsteen (3), Pei Hui (3), Eline M. Loosveld (3), Gulden Menderes (3), Babak Litkouhi (3), Elena Ratner (3), Dan-Arin Silasi (3), Peter E. Schwartz (3), Monique van der Vleuten (3), Serena Wong (3).

3. Bibliography

(a) molecular mechanism (146 items – 145 journal publications, 1 patent)

(1) Hanka, L. J.; Dietz, A.; Gerpheide, S. A.; Kuentzel, S. L.; Martin, D. G. CC-1065 (NSC-298223), A New Antitumor Antibiotic. Production, in Vitro Biological Activity, Microbiological Assays and Taxonomy of the Producing Microorganism. *J. Antibiot.* **1978**, *31* (12), 1211–1217. https://doi.org/10.7164/antibiotics.31.1211.

(2) Martin, D. G.; Chidester, C. G.; Duchamp, D. J.; Mizsak, S. A. Structure of CC-1065 (NSC-298223), A New Antitumor Antibiotic. *J. Antibiot.* **1980**, *33* (8), 902–903. https://doi.org/10.7164/antibiotics.33.902.

(3) Martin, D. G.; Biles, C.; Gerpheide, S. A.; Hanka, L. J.; Krueger, W. C.; Mcgovren, J. P.; Mizsak, S. A.; Neil, G. L.; Stewart, J. C.; Visser, J. CC-1065 (NSC 298223), a Potent New Antitumor Agent. Improved Production and Isolation, Characterization and Antitumor Activity. *J. Antibiot.* **1981**, *34* (9), 1119–1125. https://doi.org/10.7164/antibiotics.34.1119.

(4) Li, L. H.; Swenson, D. H.; Schpok, S. L. F.; Kuentzel, S. L.; Dayton, B. D.; Krueger, W. C. CC-1065 (NSC-298223), a Novel Antitumor Agent That Interacts Strongly with Double-Stranded DNA. *Cancer Res.* **1982**, *42*, 999–1994.

(5) Swenson, D. H.; Li, L. H.; Hurley, L. H.; Rokem, J. S.; Petzold, G. L.; Dayton, B. D.; Wallace, T. L.; Krueger, W. C. Mechanism of Interaction of CC-1065 (NSC-298223) with DNA. *Cancer Res.* **1982**, *42*, 2821–2828.

(6) Bhuyan, B. K.; Crampton, S. L.; Adams, E. G. Cell Cycle Effects of CC-1065. *Cancer Res.* **1983**, *43*, 4227–4232.

(7) Hurley, L. H.; Rokem, J. S. Biosynthesis of the Antitumor Antibiotic CC-1065 by Streptomyces Zelensis. *J. Antibiot.* **1983**, *36* (4), 383–390. https://doi.org/10.7164/antibiotics.36.383.

(8) Hurley, L. H.; Reynolds, V. L.; Swenson, D. H.; Petzold, G. L.; Scahill, T. A. Reaction of the Antitumor Antibiotic CC-1065 with DNA: Structure of a DNA Adduct with DNA Sequence Specificity. *Science* **1984**, *226* (4676), 843–844. https://doi.org/10.1126/science.6494915.

(9) McGovren, J. P.; Clarke, G. L.; Pratt, E. A.; DeKoning, T. F. Preliminary Toxicity Studies with the DNA-Binding Antibiotic CC-1065. *J. Antibiot.* **1984**, *37* (1), 63–70. https://doi.org/10.7164/antibiotics.37.63.

(10) Krueger, W. C.; Li, L. H.; Moscowitz, A.; Prairie, M. D.; Petzold, G.; Swenson, D. H. Binding of CC-1065 to Poly- and Oligonucleotides. *Biopolymers* **1985**, *24* (8), 1549–1572. https://doi.org/10.1002/bip.360240811.

(11) Martin, D. G.; Mizsak, S. A.; Krueger, W. C. CC-1065 Transformations. *J. Antibiot.* **1985**, *38* (6), 746–752. https://doi.org/10.7164/antibiotics.38.746.

(12) Reynolds, V. L.; Molineux, I. J.; Kaplan, D. J.; Swenson, D. H.; Hurley, L. H. Reaction of the Antitumor Antibiotic CC-1065 with DNA. Location of the Site of Thermally Induced Strand Breakage and Analysis of DNA Sequence Specificity. *Biochemistry* **1985**, *24* (22), 6228–6237. https://doi.org/10.1021/bi00343a029.

(13) Harbach, P. R.; Trzos, R. J.; Zimmer, D. M.; Petzold, G. L.; Bhuyan, B. K. Genotoxicity of the Antitumor Antibiotic CC-1065. *Mutagenesis* **1986**, *1* (6), 407–410. https://doi.org/10.1093/mutage/1.6.407.

(14) Jacobson, M. K.; Twehous, D.; Hurley, L. H. Depletion of Nicotinamide Adenine Dinucleotide in Normal and Xeroderma Pigmentosum Fibroblast Cells by the Antitumor Drug CC-1065. *Biochemistry* **1986**, *25* (20), 5929–5932. https://doi.org/10.1021/bi00368a014.

(15) Krueger, W. C.; Duchamp, D. J.; Li, L. H.; Moscowitz, A.; Petzold, G. L.; Prairie, M. D.; Swenson, D. H. The Binding of CC-1065 to Thymidine and Deoxyadenosine Oligonucleotides and to Poly(DA) · Poly(DT). *Chem.-Biol. Int.* **1986**, *59*, 55–72. https://doi.org/10.1016/S0009-2797(86)80055-2.

(16) Needham-VanDevanter, D. R.; Hurley, L. H. Construction and Characterization of a Site-Directed CC-1065-N3-Adenine Adduct within a 117 Base Pair DNA Restriction Fragment. *Biochemistry* **1986**, *25* (26), 8430–8436. https://doi.org/10.1021/bi00374a016.

(17) Reynolds, V. L.; McGovren, J. P.; Hurley, L. H. The Chemistry, Mechanism of Action and Biological Properties of CC-1065, a Potent Antitumor Antibiotic. *J. Antibiot.* **1986**, *39* (3), 319–333. https://doi.org/10.7164/antibiotics.39.319.

(18) Wierenga, W.; Bhuyan, B. K.; Kelly, R. C.; Krueger, W. C.; Li, L. H.; McGovren, J. P.; Swenson, D. H.; Warpehoski, M. A. Antitumor Activity and Biochemistry of Novel Analogs of the Antibiotic CC-1065. *Adv. Enz. Reg.* **1986**, *25*, 141–155. https://doi.org/10.1016/0065-2571(86)90012-9.

(19) Hurley, L. H.; Needham-Vandevantert, D.; Lee, C.-S. Demonstration of the Asymmetric Effect of CC-1065 on Local DNA Structure Using a Site-Directed Adduct in a 117-Base-Pair Fragment from M13mpl. *Proc. Natl. Acad. Sci. USA* **1987**, *84*, 6412–6416. https://doi.org/10.1073/pnas.84.18.6412.

(20) Krueger, W. C.; Prairie, M. D. A Circular Dichroism Study of the Binding of CC-1065 to B and Z Form Poly(Dl-5BrdC)·poly(Dl-5BrdC). *Chem.-Biol. Int.* **1987**, *62* (3), 281–295. https://doi.org/10.1016/0009-2797(87)90028-7.

(21) Zakrzewska, K.; Randrianarivelo, M.; Pullman, B. Theoretical Study of the Sequence Specificity in the Covalent Binding of the Antitumor Drug CC-1065 to DNA. *Nucl. Acid Res.* **1987**, *15* (14), 5775–5785. https://doi.org/10.1093/nar/15.14.5775.

(22) Adams, E. G.; Badiner, G. J.; Bhuyan, B. K. Effects of U-71,184 and Several Other CC-1065 Analogues on Cell Survival and Cell Cycle of Chinese Hamster Ovary Cells. *Cancer Res.* **1988**, *48*, 109–116.

(23) Boger, D. L.; Coleman, R. S. Total Synthesis of (+)-CC-1065 and Ent-(-)-CC-1065. *J. Am. Chem. Soc.* **1988**, *110* (4), 1321–1323. https://doi.org/10.1021/ja00212a067.

(24) Harbach, P. R.; Zimmer, D. M.; Mazurek, J. H.; Bhuyan, B. K. Mutagenicity of the Antitumor Antibiotic CC-1065 and Its Analogues in Mammalian (V79) Cells and Bacteria. *Cancer Res.* **1988**, *48*, 32–36.

(25) Hurley, L. H.; Lee, C. S.; McGovren, J. P.; Warpehoski, M. A.; Mitchell, M. A.; Kelly, R. C.; Aristoff, P. A. Molecular Basis for Sequence-Specific DNA Alkylation by CC-1065. *Biochemistry* **1988**, *27* (10), 3886–3892. https://doi.org/10.1021/bi00410a054.

(26) Selby, C. P.; Sancar, A. ABC Excinuclease Incises Both 5' and 3' to the CC-1065-DNA Adduct and Its Incision Activity Is Stimulated by DNA Helicase II and DNA Polymerase I. *Biochemistry* **1988**, *27* (19), 7184–7188. https://doi.org/10.1021/bi00419a004.

(27) Swenson, D. H.; Petzold, G. L.; Williams, M. G.; Li, L. H.; Prairie, M. D.; Krueger, W. C. Evaluation of DNA Binding Characteristics of Some CC-1065 Analogs. *Chem.-Biol. Int.* **1988**, *67* (3–4), 199–213. https://doi.org/10.1016/0009-2797(88)90058-0.

(28) Takahashi, I.; Takahashi, K.-I.; Ichimura, M.; Morimoto, M.; Asano, K.; Kawamoto, I.; Tomita, F.; Nakano, H. Duocarmycin A, a New Antitumor Antibiotic from Streptomyces. *J. Antibiot.* **1988**, *41* (12), 1915–1917. https://doi.org/10.7164/antibiotics.41.1915.

(29) Tang, M. S.; Lee, C. S.; Doisy, R.; Ross, L.; Needham-VanDevanter, D. R.; Hurley, L. H. Recognition and Repair of the CC-1065-(N3-Adenine)-DNA Adduct by the UVRABC Nucleases. *Biochemistry* **1988**, *27* (3), 893–901. https://doi.org/10.1021/bi00403a009.

(30) Thériault, N. Y.; Krueger, W. C.; Prairie, M. D. Studies on the Base Pair Binding Specificity of CC-1065 to Oligomer Duplexes. *Chem.-Biol. Int.* **1988**, *65* (2), 187–201. https://doi.org/10.1016/0009-2797(88)90054-3.

(31) Warpehoski, M. A.; Gebhard, I.; Kelly, R. C.; Krueger, W. C.; Li, L. H.; McGovren, J. P.; Prairie, M. D.; Wicnienski, N. A.; Wierenga, W. Stereoelectronic Factors Influencing the Biological Activity and DNA Interaction of Synthetic Antitumor Agents Modeled on CC-1065. *J. Med. Chem.* **1988**, *31* (3), 590–603. https://doi.org/10.1021/jm00398a017.

(32) Moy, B. C.; Prairie, M. D.; Krueger, W. C.; Bhuyan, B. K. Interaction of CC-1065 and Its Analogues with Mouse DNA and Chromatin. *Cancer Res.* **1989**, *49*, 1983–1988.

(33) Boger, D. L.; Invergo, B. J.; Coleman, R. S.; Zarrinmayeh, H.; Kitos, P. A.; Thompson, S. C.; Leong, T.; McLaughlin, L. A Demonstration of the Intrinsic Importance of Stabilizing Hydrophobic Binding and Non-Covalent Van-Der-Waals Contacts Dominant in the Non-Covalent CC-1065/DNA Binding. *Chem.-Biol. Int.* **1990**, *73*, 29–52. https://doi.org/10.1016/0009-2797(90)90107-x.

(34) Boger, D. L.; Zarrinmayeh, H. Regiocontrolled Nucleophilic Addition to Selectively Activated P-Quinone Diimines: Alternative Preparation of a Key Intermediate Employed in the Preparation of the CC-1065 Left-Hand Subunit. *J. Org. Chem.* **1990**, *55* (4), 1379–1390. https://doi.org/10.1021/jo00291a059.

(35) Ichimura, M.; Ogawa, T.; Takahashi, K.-I.; Kobayashi, E.; Kawamoto, I.; Yasuzawa, T.; Takahashi, I.; Nakano, H. Duocarmycin SA, a New Antitumor Antibiotic from Streptomyces Sp. *J. Antibiot.* **1990**, *43* (8), 1037–1038. https://doi.org/10.7164/antibiotics.43.1037.

(36) Moy, B. C.; Petzold, G. L.; Badiner, G. J.; Kelly, R. C.; Aristoff, P. A.; Adams, E. G.; Li, L. H.; Bhuyan, B. K. Characterization of B16 Melanoma Cells Resistant to the CC-1065 Analogue. *Cancer Res.* **1990**, *50*, 2485–2492. https://doi.org/PMID: 2317831.

(37) Nakano, H.; Takahashi, I.; Ichimura, M.; Isao, K.; Asasno, F.; Sano, H.; Yasuzawa, T.; Morimoto, M.; Fujimoto, K. Duocarmycin A Antibiotics Derived from Certain Streptomyces Culture. US004923990 A, May 8, 1990.

(38) Scahill, T. A.; Jensen, R. M.; Swenson, D. H.; Hatzenbuhler, N. T.; Petzold, G.; Wierenga, W.; Brahme, N. D. An NMR Study of the Covalent and Noncovalent Interactions of CC-1065 and DNA. *Biochemistry* **1990**, *29* (11), 2852–2860. https://doi.org/10.1021/bi00463a031.

(39) Sugiyama, H.; Hosoda, M.; Saito, I. Covalent Alkylation of DNA with Duocarmycin A . Identification of Abasic Site Structure. *Tet. Lett.* **1990**, *31* (49), 7197–7200.

(40) Boger, D. L.; Zarrinmayeh, H.; Munk, S. A.; Kitos, P. A.; Suntornwat, O. Demonstration of a Pronounced Effect of Noncovalent Binding Selectivity on the (+)-CC-1065 DNA Alkylation and Identification of the Pharmacophore of the Alkylation Subunit. *Proc. Natl. Acad. Sci. USA* **1991**, *88* (4), 1431–1435. https://doi.org/10.1073/pnas.88.4.1431.

(41) Boger, D. L.; Ishizaki, T.; Zarrinmayeh, H. Isolation and Characterization of the Duocarmycin-Adenine DNA Adduct. *J. Am. Chem. Soc.* **1991**, *113* (17), 6645–6649. https://doi.org/10.1021/ja00017a042.

(42) Ichimura, M.; Ogawa, T.; Katsumata, S.; Takahashi, K.-I.; Takahashi, I.; Nakano, H. Duocarmycins, New Antitumor Antibiotics Produced by Streptomyces; Producing Organisms and Improved Production. *J. Antibiot.* **1991**, *44* (10), 1045–1053. https://doi.org/10.7164/antibiotics.44.1045.

(43) Krueger, W. C.; Hatzenbuhler, N. T.; Prairie, M. D.; Shea, M. H. DNA Sequence Recognition by the Antitumor Antibiotic CC-1065 and Analogs of CC-1065. *Chem.-Biol. Int.* **1991**, *79* (3), 265–286. https://doi.org/10.1016/0009-2797(91)90109-K.

(44) Krueger, W. C.; Prairie, M. D. The Origin of the DNA Induced Circular Dichroism of CC-1065 and Analogs. *Chem.-Biol. Int.* **1991**, *79* (2), 137–149. https://doi.org/10.1016/0009-2797(91)90078-L.

(45) Lee, C. S.; Sun, D.; Kizu, R.; Hurley, L. H. Determination of the Structural Features of (+)-CC-1065 That Are Responsible for Bending and Winding of DNA. *Chem. Res. Toxicol.* **1991**, *4* (2), 203–213. https://doi.org/10.1021/tx00020a013.

(46) Lee, C.-S.; Gibson, N. W. DNA Damage and Differential Cytotoxicity Produced in Human Carcinoma Cells by CC-1065 Analogues, U-73,975 and U-77,779. *Cancer Res.* **1991**, *51*, 6586–6591. https://doi.org/PMID 1742731.

(47) Lin, C. H.; Beale, J. M.; Hurley, L. H. Structure of the (+)-CC-1065-DNA Adduct: Critical Role of Ordered Water Molecules and Implications for Involvement of Phosphate Catalysis in the Covalent Reaction. *Biochemistry* **1991**, *30* (15), 3597–3602. https://doi.org/10.1021/bi00229a002.

(48) Lin, C. H.; Sun, D.; Hurley, L. H. (+)-CC-1065 Produces Bending of DNA That Appears to Resemble Adenine/Thymine Tracts. *Chem. Res. Toxicol.* **1991**, *4*, 21–26. https://doi.org/10.1021/TX00019A003.

(49) Yasuzawa, T.; Saitoh, Y.; Ichimura, M.; Takahashi, I.; Sano, H. Structure of Duocarmycin SA, a Potent Antitumor Antibiotic. *J. Antibiot.* **1991**, *44* (4), 445–447. https://doi.org/10.7164/antibiotics.44.445.

(50) Zsido, T. J.; Woynarowski, J. M.; Baker, R. M.; Gawron, L. S.; Beerman, T. A. Induction of Heat-Labile Sites in DNA of Mammalian Cells by the Antitumor Alkylating Drug CC-1065. *Biochemistry* **1991**, *30* (15), 3733–3738. https://doi.org/10.1021/bi00229a021.

(51) Boger, D. L.; Machiya, K. Total Synthesis of (+)-Duocarmycin SA. *J. Am. Chem. Soc.* **1992**, *114* (25), 10056–10058. https://doi.org/10.1021/ja00051a045.

(52) Boger, D. L.; Munk, S. A. DNA Alkylation Properties of Enhanced Functional Analogs of CC-1065 Incorporating the 1,2,9,9a-Tetrahydrocyclopropa[1,2-c]Benz[1,2-e]Indol-4-One (CBI) Alkylation Subunit. *J. Am. Chem. Soc.* **1992**, *114* (14), 5487–5496. https://doi.org/10.1021/ja00040a001.

(53) Boger, D. L.; Yun, W.; Terashima, S.; Fukuda, Y.; Nakatani, K.; Kitos, P. A.; Jin, Q. DNA Alkylation Properties of the Duocarmycins: (+)-Duocarmycin A, Epi-(+)-

Duocarmycin A, Ent-(-)-Duocarmycin A and E&Ent-(-)-Duocarmycin A. *Bioorg. Med. Chem. Lett.* **1992**, *2* (7), 759–765. https://doi.org/10.1016/S0960-894X(00)80407-2.

(54) Gomi, K.; Kobayashi, E.; Miyoshi, K.; Ashizawa, T.; Okamoto, A.; Ogawa, T.; Katsumata, S.; Mihara, A.; Okabe, M.; Hirata, T. Anticellular and Antitumor Activity of Duocarmycins, Novel Antitumor Antibiotics. *Jpn. J. Cancer Res.* **1992**, *83*, 113–120. https://doi.org/10.1111/j.1349-7006.1992.tb02360.x.

(55) Krueger, W. C.; Prairie, M. D. Calf Thymus DNA Binding/Bonding Properties of CC-1065 and Analogs as Related to Their Biological Activities and Toxicities. *Chem.-Biol. Int.* **1992**, *82* (1), 31–46. https://doi.org/10.1016/0009-2797(92)90012-A.

(56) Lin, C. H.; Patel, D. J. Site-Specific Covalent Duocarmycin A: Intramolecular DNA Triplex Complex. *J. Am. Chem. Soc.* **1992**, *114* (26), 10658–10660. https://doi.org/10.1021/ja00052a085.

(57) Lin, C. H.; Hill, G. C.; Hurley, L. H. Characterization of a 12-Mer Duplex d(GGCGGAGTTAGG).Cntdot.d(CCTAACTCCGCC) Containing a Highly Reactive (+)-CC-1065 Sequence by Proton and Phosphorus-31 NMR, Hydroxyl-Radical Footprinting, and NOESY Restrained Molecular Dynamics Calculations. *Chem. Res. Toxicol.* **1992**, *5* (2), 167–182. https://doi.org/10.1021/tx00026a005.

(58) Maine, I. P.; Sun, D.; Hurley, L. H.; Kodadek, T. The Antitumor Agent CC-1065 Inhibits Helicase-Catalyzed Unwinding of Duplex DNA. *Biochemistry* **1992**, *31* (16), 3968–3975. https://doi.org/10.1021/bi00131a012.

(59) Sun, D.; Hurley, L. H. Effect of the (+)-CC-1065-(N3-Adenine)DNA Adduct on in Vitro DNA Synthesis Mediated by Escherichia Coli DNA Polymerase. *Biochemistry* **1992**, *31* (10), 2822–2829. https://doi.org/10.1021/bi00125a025.

(60) Sun, D.; Hurley, L. H. Structure-Activity Relationships of (+)-CC-1065 Analogs in the Inhibition of Helicase-Catalyzed Unwinding of Duplex DNA. *J. Med. Chem.* **1992**, *35* (10), 1773–1782. https://doi.org/10.1021/jm00088a012.

(61) Warpehoski, M. A.; Harper, D. E.; Mitchell, M. A.; Monroe, T. J. Reversibility of the Covalent Reaction of CC-1065 and Analogs with DNA. *Biochemistry* **1992**, *31* (9), 2502–2508. https://doi.org/10.1021/bi00124a009.

(62) Zsido, T. J.; Beerman, T. A.; Meegan, R. L.; Woynarowski, J. M.; Baker, R. M. Resistance of CHO Cells Expressing P-Glycoprotein to Cyclopropylpyrroloindole (CPI) Alkylating Agents. *Biochem. Pharm.* **1992**, *43* (8), 1817–1822. https://doi.org/10.1016/0006-2952(92)90715-U.

(63) Boger, D. L.; Machiya, K.; Hertzog, D. L.; Kitos, P. A.; Holmes, D. Total Synthesis and Preliminary Evaluation of (+)- and Ent-(-)-Duocarmycin SA. *J. Am. Chem. Soc.* **1993**, *115* (20), 9025–9036. https://doi.org/10.1021/ja00073a019.

(64) Boger, D. L.; Yun, W. Reversibility of the Duocarmycin A and SA DNA Alkylation Reaction. *J. Am. Chem. Soc.* **1993**, *115* (21), 9872–9873. https://doi.org/10.1021/ja00074a093.

(65) Ding, Z.-M.; Harshey, R. M.; Hurley, L. H. (+)-CC-1065 as a Structural Probe of Mu Transposase- Induced Bending of DNA: Overcoming Limitations of Hydroxyl-Radical Footprinting. *Nucl. Acid Res.* **1993**, *21* (18), 4281–4287. https://doi.org/10.1093/nar/21.18.4281.

(66) Sugiyama, H.; Ohmori, K.; Chan, K. L.; Hosoda, M.; Asai, A.; Saito, H.; Saito, I. A Novel Guanine N3 Alkylation by Antitumor Antibiotic Duocarmycin A. *Tet. Lett.* **1993**, *34* (13), 2179–2182.

(67) Sun, D.; Lin, C. H.; Hurley, L. H. A-Tract and (+)-CC-1065-Induced Bending of DNA. Comparison of Structural Features Using Non-Denaturing Gel Analysis, Hydroxyl-Radical Footprinting, and High-Field NMR. *Biochemistry* **1993**, *32* (17), 4487–4495. https://doi.org/10.1021/bi00068a003.

(68) Yamamoto, K.; Sugiyama, H.; Kawanishi, S. Concerted DNA Recognition and Novel Site-Specific Alkylation by Duocarmycin A with Distamycin A. *Biochemistry* **1993**, *32* (4), 1059–1066. https://doi.org/10.1021/bi00055a010.

(69) Asai, A.; Nagamura, S.; Saito, H. A Novel Property of Duocarmycin and Its Analogs for Covalent Reaction with DNA. *J. Am. Chem. Soc.* **1994**, *116* (10), 4171–4177. https://doi.org/10.1021/ja00089a004.

(70) Asai, A.; Nagamura, S.; Saito, H.; Takahashi, I.; Nakano, H. The Reversible DNA-Alkylating Activity of Duocarmycin and Its Analogues. *Nucl. Acid Res.* **1994**, *22* (1), 88–93. https://doi.org/10.1093/nar/22.1.88.

(71) Boger, D. L.; Johnson, D. S.; Yun, W. (+)- and Ent-(-)-Duocarmycin SA and (+)- and Ent-(-)-N-Boc-DSA DNA Alkylation Properties. Alkylation Site Models That Accommodate the Offset AT-Rich Adenine N3 Alkylation Selectivity of the Enantiomeric Agents. *J. Am. Chem. Soc.* **1994**, *116* (5), 1635–1656. https://doi.org/10.1021/ja00084a004.

(72) Boger, D. L.; Johnson, D. S.; Yun, W.; Tarby, C. M. Molecular Basis for Sequence Selective DNA Alkylation by (+)- and Ent-(-)-CC-L065 and Related Agents: Alkylation Site Models That Accommodate the Offset AT-Rich Adenine N3 Alkylation Selectivity. *Bioorg. Med. Chem.* **1994**, *2* (2), 115–135. https://doi.org/10.1016/s0968-0896(00)82007-6.

(73) Chiang, S.-Y.; Welch, J.; Rauscher, F. J.; Beerman, T. A. Effects of Minor Groove Binding Drugs on the Interaction of TATA Box Binding Protein and TFIIA with DNA. *Biochemistry* **1994**, *33* (23), 7033–7040. https://doi.org/10.1021/bi00189a003.

(74) McHugh, M. M.; Woynarowski, J. M.; Mitchell, M. A.; Gawron, L. S.; Weiland, K. L.; Beerman, T. A. CC-1065 Bonding to Intracellular and Purified SV40 DNA: Stie Specificity and Functional Effects. *Biochemistry* **1994**, *33*, 9158–9168. https://doi.org/10.1021/bi00197a019.

(75) Sugiyama, H.; Fujiwara, T.; Ura, A.; Tashiro, T.; Yamamoto, K.; Kawanishi, S.; Saito, I. Chemistry of Thermal Degradation of Abasic Sites in DNA. Mechanistic Investigation on Thermal DNA Strand Cleavage of Alkylated DNA. *Chem. Res. Toxicol.* **1994**, *7* (5), 673–683. https://doi.org/10.1021/tx00041a013.

(76) Tang, M. S.; Qian, M.; Pao, A. Formation and Repair of Antitumor Antibiotic CC-1065-Induced DNA Adducts in the Adenine Phosphoribosyltransferase and Amplified Dihydrofolate Reductase Genes of Chinese Hamster Ovary Cells. *Biochemistry* **1994**, *33* (9), 2726–2732. https://doi.org/10.1021/bi00175a048.

(77) Wrasidlo, W.; Johnson, D. S.; Boger, D. L. Induction of Endonucleolytic DNA Fragmentation and Apoptosis by the Duocarmycins. *Bioorg. Med. Chem. Lett.* **1994**, *4* (4), 631–636. https://doi.org/10.1016/S0960-894X(01)80168-2.

(78) Boger, D. L.; Johnson, D. S. Second Definitive Test of Proposed Models for the Origin of the CC-1065 and Duocarmycin DNA Alkylation Selectivity. *J. Am. Chem.*

Soc. **1995**, *117* (4), 1443–1444. https://doi.org/10.1021/ja00109a035.

(79) Boger, D. L.; Mesini, P. DNA Alkylation Properties of CC-1065 and Duocarmycin Analogs Incorporating the 2,3,10,10a-Tetrahydrocyclopropa[d]Benzo[f]Quinol-5-One Alkylation Subunit: Identification of Subtle Structural Features That Contribute to the Regioselectivity of the Adenine N3 Alkylation Reaction. *J. Am. Chem. Soc.* **1995**, *117* (47), 11647–11655. https://doi.org/10.1021/ja00152a004.

(80) Boger, D. L.; Yun, W.; Han, N.; Johnson, D. S. CC-1065 CBI Analogs: An Example of Enhancement of DNA Alkylation Efficiency through Introduction of Stabilizing Electrostatic Interactions. *Bioorg. Med. Chem.* **1995**, *3* (6), 611–621. https://doi.org/10.1016/0968-0896(95)00048-L.

(81) Kim, D.-Y.; Shih, D. S.; Cho, D.-Y.; Swenson, D. H. Helix-Stabilizing Compounds CC-1065 and U-71,184 Bind to RNA-DNA and DNA-DNA Duplexes Containing Modified Internucleotide Linkages and Stabilize Duplexes Against Thermal Melting. *Antisense Res. Dev.* **1995**, *5* (1), 49–57. https://doi.org/10.1089/ard.1995.5.49.

(82) Kim, D.-Y.; Swenson, D. H.; Cho, D.-Y.; Taylor, H. W.; Shih, D. S. Helix-Stabilizing Agent, CC-1065, Enhances Suppression of Translation by an Antisense Oligodeoxynucleotide. *Antisense Res. Dev.* **1995**, *5* (2), 149–154. https://doi.org/10.1089/ard.1995.5.149.

(83) Linsemann, D. A.; Branstetter, D. G.; Yu, R. L.; Aaron, C. S. Lung Tumor Induction in A/J Mice and Clastogenic Effects in CD_1 Mice of the Sequence-Selective DNA Alkylating Agent (+)-CC-1065 and (-)-CC-1065. *Natural Toxins* **1995**, *3*, 32–40. https://doi.org/10.1002/nt.2620030108.

(84) Muratake, H.; Matsumura, N.; Natsume, M. Total Synthesis of Natural (+)-Duocarmycin SA. *Chem. Pharm. Bull.* **1995**, *43* (6), 1064–1066. https://doi.org/10.1248/cpb.43.1064.

(85) Boger, D. L.; Goldberg, J.; McKie, J. A. A Comparative Study of the Solvolysis Reactivity, Regioselectivity, and Stereochemistry of the Duocarmycin a and Sa Alkylation Subunits. *Bioorg. Med. Chem. Lett.* **1996**, *6* (16), 1955–1960. https://doi.org/10.1016/0960-894X(96)00346-0.

(86) Boger, D. L.; Johnson, D. S. CC-1065 and the Duocarmycins: Understanding Their Biological Function through Mechanistic Studies. *Angew. Chem. Int. Ed.* **1996**, *35* (1314), 1438–1474. https://doi.org/10.1002/anie.199614381.

(87) Boger, D. L.; McKie, J. A.; Nishi, T.; Ogiku, T. Enantioselective Total Synthesis of (+)-Duocarmycin A, *Epi* -(+)-Duocarmycin A, and Their Unnatural Enantiomers. *J. Am. Chem. Soc.* **1996**, *118* (9), 2301–2302. https://doi.org/10.1021/ja953777e.

(88) Boger, D. L.; Zhou, J.; Cai, H. Demonstration and Definition of the Noncovalent Binding Selectivity of Agents Related to CC-1065 by an Affinity Cleavage Agent: Noncovalent Binding Coincidental with Alkylation. *Bioorg. Med. Chem.* **1996**, *4* (6), 859–867. https://doi.org/10.1016/0968-0896(96)00073-9.

(89) Gunz, D.; Hess, M. T.; Naegeli, H. Recognition of DNA Adducts by Human Nucleotide Excision Repair. *J. Biol. Chem.* **1996**, *271* (41), 25089–25098. https://doi.org/10.1074/jbc.271.41.25089.

(90) Gunz, D.; Naegeli, H. A Noncovalent Binding-Translocation Mechanism for Site-Specific CC-1065-DNA Recognition. *Biochem. Pharm.* **1996**, *52* (3), 447–453. https://doi.org/10.1016/0006-2952(96)00247-X.

(91) Henderson, D.; Hurley, L. H. Specific Targeting of Protein-DNA Complexes by DNA-Reactive Drugs (+)-CC-1065 and Pluramycins. *J. Mol. Recog.* **1996**, *9*, 75–87. https://doi.org/10.1002/(sici)1099-1352(199603)9:2<75::aid-jmr247>3.0.co;2-4.

(92) Lukhtanov, E. A.; Podyminogin, M. A.; Kutyavin, I. V.; Meyer, R. B.; Gamper, H. B. Rapid and Efficient Hybridization-Triggered Crosslinking Within a DNA Duplex by an Oligodeoxyribonucleotide Bearing a Conjugated Cyclopropapyrroloindole. *Nucl. Acid Res.* **1996**, *24* (4), 683–687. https://doi.org/10.1093/nar/24.4.683.

(93) Muratake, H.; Abe, I.; Natsume, M. Preparation of Alkyl-Substituted Indoles in the Benzene Portion. Part 14. Synthesis of (+-)-Duocarmycin SA, Natural (+)-Duocarmycin SA and Non-Natural (-)-Duocarmycin SA. *Chem. Pharm. Bull.* **1996**, *44* (1), 67–79. https://doi.org/10.1248/cpb.44.67.

(94) Muratake, H.; Tonegawa, M.; Natsume, M. Alternative Synthesis of Duocarmycin SA Using a Tricyclic Heteroaromatic Intermediate Prepared by Palladium-Catalyzed Coupling Reactions. *Chem. Pharm. Bull.* **1996**, *44* (8), 1631–1633. https://doi.org/10.1248/cpb.44.1631.

(95) Sugiyama, H.; Lian, C.; Isomura, M.; Saito, I.; Wang, A. H.-J. Distamycin A Modulates the Sequence Specificity of DNA Alkylation by Duocarmycin A. *Proc. Natl. Acad. Sci. USA* **1996**, *93* (25), 14405–14410. https://doi.org/10.1073/pnas.93.25.14405.

(96) Sun, D.; Hurley, L. H.; Harshey, R. M. Structural Distortions Induced by Integration Host Factor (IHF) at the H‘ Site of Phage λ Probed by (+)-CC-1065, Pluramycin, and $KMnO_4$ and by DNA Cyclization Studies. *Biochemistry* **1996**, *35* (33), 10815–10827. https://doi.org/10.1021/bi952786x.

(97) Turner, P. R.; Denny, W. A. The Mutagenic Properties of DNA Minor-Groove Binding Ligands. *Mut. Res.* **1996**, *355*, 141–169. https://doi.org/10.1016/0027-5107(96)00027-9.

(98) Boger, D. L.; Boyce, C. W.; Johnson, D. S. PH Dependence of the Rate of DNA Alkylation for (+)-Duocarmycin SA and (+)-CCBI-TMI. *Bioorg. Med. Chem. Lett.* **1997**, *7* (2), 233–238. https://doi.org/10.1016/S0960-894X(96)00605-1.

(99) Boger, D. L.; Garbaccio, R. M. Catalysis of the CC-1065 and Duocarmycin DNA Alkylation Reaction: DNA Binding Induced Conformational Change in the Agent Results in Activation. *Bioorg. Med. Chem.* **1997**, *5* (2), 263–276. https://doi.org/10.1016/S0968-0896(96)00238-6.

(100) Boger, D. L.; McKie, J. A.; Nishi, T.; Ogiku, T. Total Synthesis of (+)-Duocarmycin A, *Epi* -(+)-Duocarmycin A and Their Unnatural Enantiomers: Assessment of Chemical and Biological Properties. *J. Am. Chem. Soc.* **1997**, *119* (2), 311–325. https://doi.org/10.1021/ja962431g.

(101) Eis, P. S.; Smith, J. A.; Rydzewski, J. M.; Case, D. A.; Boger, D. L.; Chazin, W. J. High Resolution Solution Structure of a DNA Duplex Alkylated by the Antitumor Agent Duocarmycin SA. *J. Mol. Biol.* **1997**, *272* (2), 237–252. https://doi.org/10.1006/jmbi.1997.1223.

(102) Park, H.-J.; Hurley, L. H. Covalent Modification of N3 of Guanine by (+)-CC-1065 Results in Protonation of the Cross-Strand Cytosine. *J. Am. Chem. Soc.* **1997**, *119* (3), 629–630. https://doi.org/10.1021/ja9632264.

(103) Hurley, L. H.; Lee, C. S.; McGovren, J. P.; Warpehoski, M. A.; Mitchell, M. A.; Kelly, R. C.; Aristoff, P. A. Reaction of CC-1065 and Select Synthetic Analogs with

DNA. *Biochem. Pharm.* **1998**, *37* (9), 1795–1796. https://doi.org/10.1016/0006-2952(88)90450-9.

(104) Asai, A.; Yano, K.; Mizukami, T.; Nakano, H. Characterization of a Duocarmycin-DNA Adduct-Recognizing Protein in Cancer Cells. *Cancer Res.* **1999**, *59*, 5417–5420. https://doi.org/PMID 10554008.

(105) Boger, D. L.; Garbaccio, R. M. Are the Duocarmycin and CC-1065 DNA Alkylation Reactions Acid-Catalyzed? Solvolysis PH−Rate Profiles Suggest They Are Not. *J. Org. Chem.* **1999**, *64* (15), 5666–5669. https://doi.org/10.1021/jo990762g.

(106) Fujiwara, T.; Tao, Z.-F.; Ozeki, Y.; Saito, I.; Wang, H.-J.; Lee, M.; Sugiyama, H. Modulation of Sequence Specificity of Duocarmycin-Dependent DNA Alkylation by Pyrrole−Imidazole Triamides. *J. Am. Chem. Soc.* **1999**, *121* (33), 7706–7707. https://doi.org/10.1021/ja991331i.

(107) Muratake, H.; Matsumura, N.; Natsume, M. Preparation of Alkyl-Substituted Indoles in the Benzene Portion. Part 15. Asymmetric Synthesis of (+)-Duocarmycin SA Using Novel Procedure for Preparation of Hydroxyindoles. *Chem. Pharm. Bull.* **1999**, *46* (4), 559–571. https://doi.org/10.1248/cpb.46.559.

(108) Reddy, B. S. P.; Sondhi, S. M.; Lown, J. W. Synthetic DNA Minor Groove-Binding Drugs. *Pharm. & Ther.* **1999**, *84*, 1–111. https://doi.org/10.1016/s0163-7258(99)00021-2.

(109) Schnell, J. R.; Ketchem, R. R.; Boger, D. L.; Chazin, W. J. Binding-Induced Activation of DNA Alkylation by Duocarmycin SA: Insights from the Structure of an Indole Derivative−DNA Adduct. *J. Am. Chem. Soc.* **1999**, *121* (24), 5645–5652. https://doi.org/10.1021/ja983556j.

(110) Tercel, M.; Gieseg, M. A.; Milbank, J. B.; Boyd, M.; Fan, J.-Y.; Tan, L. K.; Wilson, W. R.; Denny, W. A. Cytotoxicity and DNA Interaction of the Enantiomers of 6-Amino-3-(Chloromethyl)-1-[(5,6,7-Trimethoxyindol-2-Yl)Carbonyl]Indoline (Amino- *Seco* -CI-TMI). *Chem. Res. Toxicol.* **1999**, *12* (8), 700–706. https://doi.org/10.1021/tx990069o.

(111) Tokoro, Y.; Isoe, T.; Shindo, K. Gilvusmycin, a New Antitumor Antibiotic Related to CC-1065. *J. Antibiot.* **1999**, *52* (3), 263–268. https://doi.org/10.7164/antibiotics.52.263.

(112) Kirschner, K. N.; Lee, M.; Stanley, R. C.; Bowen, J. P. Density Functional and Ab Initio Studies on N -Acetyl-Duocarmycin SA: Insight into Its DNA Interaction Properties. *Bioorg. Med. Chem.* **2000**, *8* (2), 329–335. https://doi.org/10.1016/S0968-0896(99)00278-3.

(113) Nazimiec, M.; Lee, C.-S.; Tang, Y.-L.; Ye, X.; Case, R.; Tang, M. Sequence-Dependent Interactions of Two Forms of UvrC with DNA Helix-Stabilizing CC-1065−N3-Adenine Adducts. *Biochemistry* **2001**, *40* (37), 11073–11081. https://doi.org/10.1021/bi010953p.

(114) Skladanowski, A.; Koba, M.; Konopa, J. Does the Antitumor Cyclopropylpyrroloindole Antibiotic CC-1065 Cross-Link DNA in Tumor Cells? *Biochem. Pharm.* **2001**, *61* (1), 67–72. https://doi.org/10.1016/S0006-2952(00)00528-1.

(115) Ambroise, Y.; Boger, D. L. The DNA Phosphate Backbone Is Not Involved in Catalysis of the Duocarmycin and CC-1065 DNA Alkylation Reaction. *Bioorg. Med. Chem. Lett.* **2002**, *12* (3), 303–306. https://doi.org/10.1016/S0960-894X(01)00740-5.

(116) Kiakos, K.; Howard, T. T.; Lee, M.; Hartley, J. A.; McHugh, P. J. Saccharomyces Cerevisiae RAD5 Influences the Excision Repair of DNA Minor Groove Adducts. *J. Biol. Chem.* **2002**, *277* (46), 44576–44581. https://doi.org/10.1074/jbc.M208169200.

(117) Park, H.-J. Evidence for a Common Molecular Basis for Sequence Recognition of N3-Guanine and N3-Adenine DNA Adducts Involving the Covalent Bonding Reaction of (+)-CC-1065. *Arch. Pharm. Res.* **2002**, *25* (1), 11–24. https://doi.org/10.1007/BF02975255.

(118) Igarashi, Y.; Futamata, K.; Fujita, T.; Sekine, A.; Senda, H.; Naoki, H.; Furumai, T. Yatakemycin, a Novel Antifungal Antibiotic Produced by Streptomyces Sp. TP-A0356. *J. Antibiot.* **2003**, *56* (2), 107–113. https://doi.org/10.7164/antibiotics.56.107.

(119) Kuwabara, T.; Noda, T.; Ohtake, H.; Ohtake, T.; Toyama, S.; Ikariyama, Y. Classification of DNA-Binding Mode of Antitumor and Antiviral Agents by the Electrochemiluminescence of Ruthenium Complex. *Anal. Biochem.* **2003**, *314* (1), 30–37. https://doi.org/10.1016/S0003-2697(02)00651-6.

(120) Parrish, J. P.; Kastrinsky, D. B.; Wolkenberg, S. E.; Igarashi, Y.; Boger, D. L. DNA Alkylation Properties of Yatakemycin. *J. Am. Chem. Soc.* **2003**, *125* (36), 10971–10976. https://doi.org/10.1021/ja035984h.

(121) Tietze, L. F.; Haunert, F.; Feuerstein, T.; Herzig, T. A Concise and Efficient Synthesis of Seco-Duocarmycin SA. *Eur. J. Org. Chem.* **2003**, *2003* (3), 562–566. https://doi.org/10.1002/ejoc.200390094.

(122) LaBarbera, D. V.; Skibo, E. B. Solution Kinetics of CC-1065 A-Ring Opening: Substituent Effects and General Acid/Base Catalysis. *J. Am. Chem. Soc.* **2006**, *128* (11), 3722–3727. https://doi.org/10.1021/ja057289a.

(123) Okano, K.; Tokuyama, H.; Fukuyama, T. Total Synthesis of (+)-Yatakemycin. *J. Am. Chem. Soc.* **2006**, *128* (22), 7136–7137. https://doi.org/10.1021/ja0619455.

(124) Spiegel, K.; Rothlisberger, U.; Carloni, P. Duocarmycins Binding to DNA Investigated by Molecular Simulation. *J. Phys. Chem. B* **2006**, *110* (8), 3647–3660. https://doi.org/10.1021/jp0548265.

(125) Tichenor, M. S.; Trzupek, J. D.; Kastrinsky, D. B.; Shiga, F.; Hwang, I.; Boger, D. L. Asymmetric Total Synthesis of (+)- and *Ent* -(−)-Yatakemycin and Duocarmycin SA: Evaluation of Yatakemycin Key Partial Structures and Its Unnatural Enantiomer. *J. Am. Chem. Soc.* **2006**, *128* (49), 15683–15696. https://doi.org/10.1021/ja064228j.

(126) Trzupek, J. D.; Gottesfeld, J. M.; Boger, D. L. Alkylation of Duplex DNA in Nucleosome Core Particles by Duocarmycin SA and Yatakemycin. *Nat. Chem. Biol.* **2006**, *2* (2), 79–82. https://doi.org/10.1038/nchembio761.

(127) Hirota, M.; Fujiwara, T.; Mineshita, S.; Sugiyama, H.; Teraoka, H. Distamycin A Enhances the Cytotoxicity of Duocarmycin A and Suppresses Duocarmycin A-Induced Apoptosis in Human Lung Carcinoma Cells. *Int. J. Biochem. Cell Biol.* **2007**, *39* (5), 988–996. https://doi.org/10.1016/j.biocel.2007.01.019.

(128) MacMillan, K. S.; Boger, D. L. An Additional Spirocyclization for Duocarmycin SA. *J. Am. Chem. Soc.* **2008**, *130* (49), 16521–16523. https://doi.org/10.1021/ja806593w.

(129) Tichenor, M. S.; Boger, D. L. Yatakemycin: Total Synthesis, DNA Alkylation, and Biological Properties. *Nat. Prod. Rep.* **2008**, *25* (2), 220–226. https://doi.org/10.1039/B705665F.

(130) Zhong, H.; Kirschner, K. N.; Lee, M.; Bowen, J. P. Binding Free Energy Calculation for Duocarmycin/DNA Complex Based on the QPLD-Derived Partial Charge Model. *Bioorg. Med. Chem. Lett.* **2008**, *18* (2), 542–545. https://doi.org/10.1016/j.bmcl.2007.11.090.

(131) MacMillan, K. S.; Boger, D. L. Fundamental Relationships between Structure, Reactivity, and Biological Activity for the Duocarmycins and CC-1065. *J. Med. Chem.* **2009**, *52* (19), 5771–5780. https://doi.org/10.1021/jm9006214.

(132) Huang, W.; Xu, H.; Li, Y.; Zhang, F.; Chen, X.-Y.; He, Q.-L.; Igarashi, Y.; Tang, G.-L. Characterization of Yatakemycin Gene Cluster Revealing a Radical *S* -Adenosylmethionine Dependent Methyltransferase and Highlighting Spirocyclopropane Biosynthesis. *J. Am. Chem. Soc.* **2012**, *134* (21), 8831–8840. https://doi.org/10.1021/ja211098r.

(133) Yoshidome, T.; Endo, M.; Kashiwazaki, G.; Hidaka, K.; Bando, T.; Sugiyama, H. Sequence-Selective Single-Molecule Alkylation with a Pyrrole–Imidazole Polyamide Visualized in a DNA Nanoscaffold. *J. Am. Chem. Soc.* **2012**, *134* (10), 4654–4660. https://doi.org/10.1021/ja209023u.

(134) Tercel, M.; McManaway, S. P.; Leung, E.; Liyanage, H. D. S.; Lu, G.-L.; Pruijn, F. B. The Cytotoxicity of Duocarmycin Analogues Is Mediated through Alkylation of DNA, Not Aldehyde Dehydrogenase 1: A Comment. *Angew. Chem. Int. Ed.* **2013**, *52* (21), 5442–5446. https://doi.org/10.1002/anie.201208373.

(135) Chandran, A.; Syed, J.; Taylor, R. D.; Kashiwazaki, G.; Sato, S.; Hashiya, K.; Bando, T.; Sugiyama, H. Deciphering the Genomic Targets of Alkylating Polyamide Conjugates Using High-Throughput Sequencing. *Nucl. Acid Res.* **2016**, *44* (9), 4014–4024. https://doi.org/10.1093/nar/gkw283.

(136) Lin, J.; Hiraoka, K.; Watanabe, T.; Kuo, T.; Shinozaki, Y.; Takatori, A.; Koshikawa, N.; Chandran, A.; Otsuki, J.; Sugiyama, H.; Horton, P.; Nagase, H. Identification of Binding Targets of a Pyrrole-Imidazole Polyamide KR12 in the LS180 Colorectal Cancer Genome. *PLoS ONE* **2016**, *11* (10), e0165581. https://doi.org/10.1371/journal.pone.0165581.

(137) Zou, T.; Kizaki, S.; Pandian, G. N.; Sugiyama, H. Nucleosome Assembly Alters the Accessibility of the Antitumor Agent Duocarmycin B $_2$ to Duplex DNA. *Chem. Eur. J.* **2016**, *22* (26), 8756–8758. https://doi.org/10.1002/chem.201600950.

(138) Mullins, E. A.; Shi, R.; Eichman, B. F. Toxicity and Repair of DNA Adducts Produced by the Natural Product Yatakemycin. *Nat. Chem. Biol.* **2017**, *13* (9), 1002–1008. https://doi.org/10.1038/nchembio.2439.

(139) Wu, S.; Jian, X.-H.; Yuan, H.; Jin, W.-B.; Yin, Y.; Wang, L.-Y.; Zhao, J.; Tang, G.-L. Unified Biosynthetic Origin of the Benzopyrrole Subunit in CC-1065. *ACS Chem. Biol.* **2017**, *12*, 1603–1610. https://doi.org/10.1021/acschembio.7b00302.

(140) Yuan, H.; Zhang, J.; Cai, Y.; Wu, S.; Yang, K.; Chan, H. C. S.; Huang, W.; Jin, W.-B.; Li, Y.; Yin, Y.; Igarashi, Y.; Yuan, S.; Zhou, J.; Tang, G.-L. GyrI-like Proteins Catalyze Cyclopropanoid Hydrolysis to Confer Cellular Protection. *Nat. Comm.* **2017**, *8* (1), 1485. https://doi.org/10.1038/s41467-017-01508-1.

(141) Boyle, K. E.; Boger, D. L.; Wroe, A.; Vazquez, M. Duocarmycin SA, a Potent Antitumor Antibiotic, Sensitizes Glioblastoma Cells to Proton Radiation. *Bioorg. Med. Chem. Lett.* **2018**, *28* (16), 2688–2692. https://doi.org/10.1016/j.bmcl.2018.04.008.

(142) Jin, W.-B.; Wu, S.; Jian, X.-H.; Yuan, H.; Tang, G.-L. A Radical S-Adenosyl-L-Methionine Enzyme and a Methyltransferase Catalyze Cyclopropane Formation in Natural Product Biosynthesis. *Nat. Comm.* **2018**, *9* (1), 2771. https://doi.org/10.1038/s41467-018-05217-1.

(143) Kashiwazaki, G.; Maeda, R.; Kawase, T.; Hashiya, K.; Bando, T.; Sugiyama, H. Evaluation of Alkylating Pyrrole-Imidazole Polyamide Conjugates by a Novel Method for High-Throughput Sequencer. *Bioorg. Med. Chem.* **2018**, *26* (1), 1–7. https://doi.org/10.1016/j.bmc.2017.08.057.

(144) Schmidt, M. A.; Simmons, E. M.; Wei, C. S.; Park, H.; Eastgate, M. D. An Enantioselective Total Synthesis of (+)-Duocarmycin SA. *J. Org. Chem.* **2018**, *83* (7), 3928–3940. https://doi.org/10.1021/acs.joc.8b00285.

(145) Wang, X.; Wu, S.; Jin, W.; Xu, B.; Tang, G.; Yuan, H. Bioinformatics-Guided Connection of a Biosynthetic Gene Cluster to the Antitumor Antibiotic Gilvusmycin. *Acta Biochim. Biophys. Sin.* **2018**, *50* (5), 516–518. https://doi.org/10.1093/abbs/gmy030.

(146) Imaizumi, T.; Yamashita, Y.; Nakazawa, Y.; Okano, K.; Sakata, J.; Tokuyama, H. Total Synthesis of (+)-CC-1065 Utilizing Ring Expansion Reaction of Benzocyclobutenone Oxime Sulfonate. *Org. Lett.* **2019**, *21* (16), 6185–6189. https://doi.org/10.1021/acs.orglett.9b01690.

(b) early clinical trials (49 journal publications)

(1) Li, L. H.; Kelly, RobertC.; Warpehoski, MarthaA.; McGovren, J. P.; Gebhard, I.; DeKoning, ThomasF. Adozelesin, a Selected Lead among Cyclopropylpyrroloindole Analogs of the DNA-Binding Antibiotic, CC-1065. *Invest. New Drugs* **1991**, *9* (2). https://doi.org/10.1007/BF00175081.

(2) Mitchell, M. A.; Kelly, R. C.; Wicnienski, N. A.; Hatzenbuhler, N. T.; Williams, M. G.; Petzold, G. L.; Slightom, J. L.; Siemieniak, D. R. Synthesis and DNA Crosslinking by a Rigid CPI Dimer. *J. Am. Chem. Soc.* **1991**, *113* (23), 8994–8995. https://doi.org/10.1021/ja00023a085.

(3) Weiland, K. L.; Dooley, T. P. In Vitro and in Vivo DNA Bonding by the CC-1065 Analog U-73975. *Biochemistry* **1991**, *30* (30), 7559–7565. https://doi.org/10.1021/bi00244a027.

(4) Bhuyan, B. K.; Smith, K. S.; Adams, E. G.; Petzold, G. L.; McGovren, J. P. Lethality, DNA Alkylation, and Cell Cycle Effects of Adozelesin (U-73975) on Rodent and Human Cells. *Cancer Res.* **1992**, *52*, 5687–5692.

(5) Bhuyan, B. K.; Smith, K. S.; Adams, E. G.; Wallace, T. L.; Von Hoff, D. D.; Li, L. H. Adozelesin, a Potent New Alkylating Agent: Cell-Killing Kinetics and Cell-Cycle Effects. *Cancer Chemother. Pharmacol.* **1992**, *30* (5), 348–354. https://doi.org/10.1007/BF00689961.

(6) Li, L. H.; DeKoning, T. F.; Kelly, R. C.; Krueger, W. C.; McGovren, J. P.; Padbury, G. E.; Petzold, G. L.; Wallace, T. L.; Ouding, R. J.; Prairie, M. D.; Gebhard, I. Cytotoxicity and Antitumor Activity of Carzelesin, a Prodrug Cyclopropylpyrroloindole Analogue. *Cancer Res.* **1992**, *52*, 4904–4913.

(7) Nguyen, H. N.; Sevin, B.-U.; Averette, H.; Perras, J.; Ramos, R.; Donato, D. Spectrum of Cell-Cycle Kinetics of Alkylating Agent Adolezesin in Gynecological Cancer Cell Lines: Correlation with Drug-Induced Cytotoxicity. *J. Cancer Res. Clin. Oncol.* **1992**, *118* (7), 515–522. https://doi.org/10.1007/BF01225266.

(8) Hightower, R. D.; Sevin, B.-U.; Perras, J.; Nguyen, H.; Angioli, R.; Untch, M.; Averette, H. In Vitro Evaluation of the Novel Chemotherapeutic Agents U-73, 975, U-77, 779, and U-80, 244 in Gynecologic Cancer Cell Lines. *Cancer Invest.* **1993**, *11* (3), 276–282. https://doi.org/10.3109/07357909309024852.

(9) Monroe, T. J.; Mitchell, M. A. In Vivo Mutagenesis Induced by CC-1065 and Adozelesin DNA Alkylation in a Transgenic Mouse Model. *Cancer Res.* **1993**, *53*, 5690–5696.

(10) Sun, D.; Park, H. J.; Hurley, L. H. Alkylation of Guanine and Cytosine in DNA by Bizelesin. Evidence for a Covalent Immobilization Leading to a Proximity-Driven Alkylation of Normally Unreactive Bases by a (+)-CC-1065 Cross-Linking Compound. *Chem. Res. Toxicol.* **1993**, *6* (6), 889–894. https://doi.org/10.1021/tx00036a020.

(11) Fleming, G. F.; Ratain, M. J.; O'Brien, S. M.; Schilsky, R. L.; Hoffman, P. C.; Richards, J. M.; Vogelzang, N. J.; Kasunic, D. A.; Earhart, R. H. Phase I Study of Adozelesin Administered by 24-Hour Continuous Intravenous Infusion. *J. Natl. Cancer Inst.* **1994**, *86* (5), 368–372. https://doi.org/10.1093/jnci/86.5.368.

(12) Kobayashi, E.; Okamoto, A.; Asada, M.; Okabe, M.; Nagamura, S.; Asai, A.; Saito, H.; Gomi, K.; Hirata, T. Characteristics of Antitumor Activity of KW-2189, a Novel Water-Soluble Derivative of Duocarmycin, against Murine and Human Tumors. *Cancer Res.* **1994**, *54*, 2404–2410.

(13) Ogasawara, H.; Nishio, K.; Takeda, Y.; Ohmori, T.; Kubota, N.; Funayama, Y.; Ohira, T.; Kuraishi, Y.; Isogai, Y.; Saijo, N. A Novel Antitumor Antibiotic, KW-2189 Is Activated by Carboxyl Esterase and Induces DNA Strand Breaks in Human Small Cell Lung Cancer Cells. *Jpn. J. Cancer Res.* **1994**, *85*, 418–425. https://doi.org/10.1111/j.1349-7006.1994.tb02375.x.

(14) Okamoto, A.; Asai, A.; Saito, H.; Okabe, M.; Gomi, K. Differential Effect of Duocarmycin A and Its Novel Derivative DU-86 on DNA Strand Breaks in HeLa S3 Cells. *Jpn. J. Cancer Res.* **1994**, *85*, 1304–1311. https://doi.org/10.1111/j.1349-7006.1994.tb02944.x.

(15) Shamdas, G. J.; Alberts, D. S.; Modiano, M.; Wiggins, C.; Power, J.; Kasunic, D. A.; Elfring, G. L.; Earhart, R. H. Phase I Study of Adozelesin (U-73,975) in Patients with Solid Tumors. *Anti-Cancer Drugs* **1994**, *5*, 10–14. https://doi.org/10.1097/00001813-199402000-00002.

(16) van Tellingen, O.; Pels, E. M.; Henrar, R. E. C.; Schaaf, L. J.; Padbury, G. E.; Beijnen, J. H.; Nooijen, W. J. Fully Automated High-Performance Liquid Chromatographic Method for the Determination of Carzelesin (U-80,244) and Metabolites (U-76,073 and U-76,074) in Human Plasma. *J. Chromat. B: Biomed. Sci. Appl.* **1994**, *652* (1), 51–58. https://doi.org/10.1016/0378-4347(93)E0377-3.

(17) Walker, D. L.; Reid, J. M.; Ames, M. M. Preclinical Pharmacology of Bizelesin, a Potent Bifunctional Analog of the DNA-Binding Antibiotic CC-1065. *Cancer Chemother. Pharmacol.* **1994**, *34*, 317–322.

(18) Houghton, P. J.; Cheshire, P. J.; Hallman II, J. D.; Houghton, J. A. Therapeutic Efficacy of the Cyclopropylpyrroloindole, Carzelesin, against Xenografts Derived from Adult and Childhood Solid Tumors. *Cancer Chemother. Pharmacol.* **1995**, *36*, 45–52. https://doi.org/10.1007/BF00685731.

(19) Ogasawara, H.; Nishio, K.; Kanzawa, F.; Lee, Y.-S.; Funayama, Y.; Ohira, T.; Kuraishi, Y.; Isogai, Y.; Saijo, N. Intracellular Carboxyl Esterase Activity Is a Determinant of Cellular Sensitivity to the Antineoplastic Agent KW-2189 in Cell Lines Resistant to Cisplatin and CPT-11. *Jpn. J. Cancer Res.* **1995**, *86*, 124–129. https://doi.org/10.1111/j.1349-7006.1995.tb02997.x.

(20) Smith, K. S.; Folz, B. A.; Adams, E. G.; Bhuyan, B. K. Synergistic and Additive Combinations of Several Antitumor Drugs and Other Agents with the Potent Alkylating Agent Adozelesin. *Cancer Chemother. Pharmacol.* **1995**, *35*, 471–482.

(21) Carter, C. A.; Waud, W. R.; Li, L. H.; DeKoning, T. F.; McGovren, J. P.; Plowman, J. Preclinical Antitumor Activity of Bizelesin in Mice. *Clin. Cancer Res.* **1996**, *2*, 1143–1149.

(22) Foster, B. J.; LoRusso, P. M.; Poplin, E.; Zalupski, M.; Valdivieso, M.; Wozniak, A.; Flaherty, L.; Kasunic, D. A.; Earhart, R. H.; Baker, L. H. Phase I Trial of Adozelesin Using the Treatment Schedule of Daily x 5 Every 3 Weeks. *Invest. New Drugs* **1996**, *13*, 321–326. https://doi.org/10.1007/BF00873138.

(23) Jonkman-De Vries, J. D.; Doppenberg, W. G.; Henrar, R. E. C.; Bult, A.; Beijnen, J. H. Systematic Study on the Chemical Stability of the Prodrug Antitumor Agent Carzelesin (U-80,244). *J. Pharm. Sci.* **1996**, *85* (11), 1227–1233. https://doi.org/10.1021/js960005n.

(24) Nagamura, S.; Kobayashi, E.; Gomi, K.; Saito, H. Studies on the Active Metabolite (DU-86) of KW-2189, a Novel Derivative of Duocarmycin. *Bioorg. Med. Chem. Lett.* **1996**, *6* (17), 2147–2150. https://doi.org/10.1016/0960-894X(96)00388-5.

(25) Volpe, D. A.; Tomaszewski, J. E.; Parchment, R. E.; Garg, A.; Flora, K. P.; Murphy, M. J.; Grieshaber, C. K. Myelotoxic Effects of the Bifunctional Alkylating Agent Bizelesin on Human, Canine and Murine Myeloid Progenitor Cells. *Cancer Chemother. Pharmacol.* **1996**, *39* (1–2), 143–149. https://doi.org/10.1007/s002800050550.

(26) Wolff, I.; Bench, K.; Beijnen, J. H.; Bruntsch, U.; Cavalli, F.; de Jong, J.; Groot, Y.; von Tellingen, O.; Wanders, J.; Sessa, C. Phase I Clinical and Pharmacokinetic Study of Carzelesin (U-80244) Fiven Daily for Five Consecutive Days. *Clin. Cancer Res.* **1996**, *2*, 1717–1723.

(27) Burris, H. A.; Dieras, V. C.; Tunca, M.; Earhart, R. H.; Eckardt, J. R.; Rodriguey, G. I.; Shaffer, D. S.; Fields, S. M.; Campbell, E.; Schaaf, L.; Kasunic, D. A.; Von Hoff, D. D. Phase I Study with the DNA Sequence-Specific Agent Adozelesin. *Anti-Cancer Drugs* **1997**, *8*, 588–596. https://doi.org/10.1097/00001813-199707000-00006.

(28) Nagamura, S.; Kinugawa, M.; Ogasa, T.; Saito, H. The Synthesis of [3H]KW-2189, a Novel Antitumor Antibiotic. *J. Lab. Comp. Radiopharm.* **1997**, *39* (6), 471–477.

(29) Ogasawara, H.; Nishio, K.; Ishida, T.; Arioka, H.; Fukuoka, K.; Saijo, N. In Vitro Enhancement of Antitumor Activity of a Water-Soluble Duocarmycin Derivative, KW-2189, by Caffeine-Mediated DNA-Repair Inhibition in Human Lung Cancer Cells. *Jpn. J. Cancer Res.* **1997**, *88*, 1033–1037. https://doi.org/10.1111/j.1349-7006.1997.tb00326.x.

(30) Alberts, S. R.; Erlichman, C.; Reid, J. M.; Sloan, J. A.; Ames, M. M.; Richardson, R. L.; Goldberg, R. M. Phase I Study of the Duocarmycin Semisynthetic Derivative KW-

2189 given Daily for Five Days Every Six Weeks. *Clin. Cancer Res.* **1998**, *4*, 2111–2117.

(31) Cristofanilli, M.; Bryan, W. J.; Miller, L. L.; Chang, A. Y.-C.; Gradishar, W. J.; Kufe, D. W.; Hortobagyi, G. N. Phase II Study of Adozelesin in Untreated Metastatic Breast Cancer. *Anti-Cancer Drugs* **1998**, *9*, 779–782. https://doi.org/10.1097/00001813-199810000-00006.

(32) Kinugawa, M.; Nagamura, S.; Sakaguchi, A.; Masuda, Y.; Saito, H.; Ogasa, T.; Kasai, M. Practical Synthesis of the High-Quality Antitumor Agent KW-2189 from Duocarmycin B2 Using a Facile One-Pot Synthesis of an Intermediate. *Org. Process Res. Dev.* **1998**, *2* (6), 344–350. https://doi.org/10.1021/op980038k.

(33) van Tellingen, O.; Punt, C. J. A.; Groot, Y.; Henrar, R. E. C.; Awada, A.; Wagener, D. J. T.; Piccart, M. J.; Schaaf, L. J.; Nooijen, W. J.; Beijnen, J. H. A Clinical Pharmacokinetics Study of Carzelesin given by Short-Term Intravenous Infusion in a Phase I Study. *Cancer Chemother. Pharmacol.* **1998**, *41* (5), 377–384. https://doi.org/10.1007/s002800050754.

(34) van Tellingen, O.; Nooijen, W. J.; Schaaf, L. J.; Henrar, R. E. C.; Beijnen, J. H. Comparative Pharmacology of the Novel Cyclopropylpyrroloindole-Prodrug Carzelesin in Mice, Rats, and Humans. *Cancer Res.* **1998**, *58*, 2410–2416.

(35) Awada, A.; Punt, C. J. A.; Piccart, M. J.; Tellingen, O. V.; Manen, L. V.; Kerger, J.; Groot, Y.; Wanders, J.; Verweij, J.; Wagener, D. J. T. Phase I Study of Carzelesin (U-80,244) given (4-Weekly) by Intravenous Bolus Schedule. *Br. J. Cancer* **1999**, *79* (9–10), 1454–1461. https://doi.org/10.1038/sj.bjc.6690232.

(36) Hidalgo, M.; Izbicka, E.; Cerna, C.; Gomez, L.; Rowinsky, E. K.; Weitman, S. D.; Von Hoff, D. D. Comparative Activity of the Cyclopropylpyrroloindole Compounds Adozelesin, Bizelein and Carzelesin in a Human Tumor Colony-Forming Assay. *Anti-Cancer Drugs* **1999**, *10*, 295–302. https://doi.org/Comparative Activity of the Cyclopropylpyrroloindole Compounds Adozelesin, Bizelein and Carzelesin in a human tumor colony-forming assay.

(37) Cameron, L.; Thompson, A. S. Determination of the Structural Role of the Linking Moieties in the DNA Binding of Adozelesin. *Biochemistry* **2000**, *39* (17), 5004–5012. https://doi.org/10.1021/bi9926532.

(38) Liu, J.-S.; Kuo, S.-R.; McHugh, M. M.; Beerman, T. A.; Melendy, T. Adozelesin Triggers DNA Damage Response Pathways and Arrests SV40 DNA Replication through Replication Protein A Inactivation. *J. Biol. Chem.* **2000**, *275* (2), 1391–1397. https://doi.org/10.1074/jbc.275.2.1391.

(39) Pavlidis, N.; Aamdal, S.; Awada, A.; Calvert, H.; Fumoleau, P.; Sorio, R.; Punt, C.; Verweij, J.; van Oosterom, A.; Morant, R.; Wanders, J.; Hanauske, A.-R. Carzelesin Phase II Study in Advanced Breast, Ovarian, Colorectal, Gastric, Head and Neck Cancer, Non-Hodgkin's Lymphoma and Malignant Melanoma: A Study of the EORTC Early Clinical Studies Group (ECSG). *Cancer Chemother. Pharmacol.* **2000**, *46* (2), 167–171. https://doi.org/10.1007/s002800000134.

(40) Small, E. J.; Figlin, R.; Petrylak, D.; Vaughn, D. J.; Sartor, O.; Horak, I.; Pincus, R.; Kremer, A.; Bowden, C. A Phase II Pilot Study of KW-2189 in Patients with Advanced Renal Cell Carcinoma. *Invest. New Drugs* **2000**, *18*, 193–197.

(41) Park, H.-J. DNA Structural Perturbation Induced by the CPI-Derived DNA Interstrand Cross-Linker: Molecular Mechanisms for the Sequence Specific Recognition. *Arch. Pharm. Res.* **2001**, *24* (5), 455–465. https://doi.org/10.1007/BF02975194.

(42) Markovic, S. N.; Suman, V. J.; Vukov, A. M.; Fitch, T. R.; Hillman, D. W.; Adjei, A. A.; Alberts, S. R.; Kaur, J. S.; Braich, T. A.; Leitch, J. M.; Creagan, E. T. Phase II Trial of KW2189 in Patients With Advanced Malignant Melanoma: *Am. J. Clin. Oncol.* **2002**, *25* (3), 308–312. https://doi.org/10.1097/00000421-200206000-00022.

(43) Pitot, H. C.; Reid, J. M.; Sloan, J. A.; Ames, M. M.; Adjei, A. A.; Rubin, J.; Bagniewski, P. G.; Atherton, P.; Rayson, D.; Goldberg, R. M.; Erlichman, C. A Phase I Study of Bizelesin (NSC 615291) in Patients with Advanced Solid Tumors. *Clin. Cancer Res.* **2002**, *8*, 712–717.

(44) Cao, P.; McHugh, M. M.; Melendy, T.; Beerman, T. The DNA Minor Groove-Alkylating Cyclopropylpyrroloindole Drugs Adozelesin and Bizelesin Induce Different DNA Damage Response Pathways in Human Colon Carcinoma HCT116 Cells. *Mol. Cancer Ther.* **2003**, *2*, 651–659.

(45) Schwartz, G. H.; Patnaik, A.; Hammond, L. A.; Rizzo, J.; Berg, K.; Von Hoff, D. D.; Rowinsky, E. K. A Phase I Study of Bizelesin, a Highly Potent and Selective DNA-Interactive Agent, in Patients with Advanced Solid Malignancies. *Ann. Oncol.* **2003**, *14* (5), 775–782. https://doi.org/10.1093/annonc/mdg215.

(46) Taguchi, F.; Kusaba, H.; Asai, A.; Iwamoto, Y.; Yano, K.; Nakano, H.; Mizukami, T.; Saijo, N.; Kato, H.; Nishio, K. HnRNP L Enhances Sensitivity of the Cells to KW-2189. *Int. J. Cancer* **2004**, *108* (5), 679–685. https://doi.org/10.1002/ijc.11616.

(47) Yanow, S. K.; Purcell, L. A.; Spithill, T. W. The A/T-Specific DNA Alkylating Agent Adozelesin Inhibits Plasmodium Falciparum Growth in Vitro and Protects Mice against Plasmodium Chabaudi Adami Infection. *Mol. Biochem. Parasit.* **2006**, *148* (1), 52–59. https://doi.org/10.1016/j.molbiopara.2006.02.019.

(48) Alberts, S. R.; Suman, V. J.; Pitot, H. C.; Camoriano, J. K.; Rubin, J. Use of KW-2189, a DNA Minor Groove-Binding Agent, in Patients with Hepatocellular Carcinoma: A North Central Cancer Treatment Group (NCCTG) Phase II Clinical Trial. *J. Gastrointest. Cancer* **2007**, *38* (1), 10–14. https://doi.org/10.1007/s12029-007-9007-6.

(49) Lee, S.-Y.; Pfeifer, G. P.; Lee, C.-S. Mutation Spectra Induced by Adozelesin in the SupF Gene of Human XP-A Fibroblasts. *Arch. Pharm. Res.* **2010**, *33* (4), 633–636. https://doi.org/10.1007/s12272-010-0419-7.

(1) SAR studies (191 items - 182 journal publications, 9 patents)

(1) Sundberg, R. J.; Nishiguchi, T. Synthesis and Intramolecular Photoaddition of an Indole Quinonediazide. *Tet. Lett.* **1983**, *24* (44), 4773–4776. https://doi.org/10.1016/S0040-4039(00)94004-8.

(2) Boger, D. L.; Coleman, R. S.; Invergo, B. J. Studies on the Total Synthesis of CC-1065: Preparation of a Synthetic Simplified 3-Carbamoyl-1,2-Dihydro-3H-Pyrrolo[3,2-e]Indole Dimer/Trimer/Tetramer (CDPI Dimer/Trimer/Tetramer) and Development of Methodology for PDE-I Dimer Methyl Ester Formation. *J. Org. Chem.* **1987**, *52* (8), 1521–1530. https://doi.org/10.1021/jo00384a026.

(3) Li, L. H.; Wallace, T. L.; DeKoning, T. F.; Warpehoski, M. A.; Kelly, R. C.; Prairie, M. D.; Krueger, W. C. Structure and Activity Relationship of Several Novel CC-1065 Analogs. *Invest. New Drugs* **1987**, *5* (4). https://doi.org/10.1007/BF00169971.

(4) Boger, D. L.; Coleman, R. S. Total Synthesis of (.+-.)-N2-(Phenylsulfonyl)-CPI, (.+-.)-CC-1065, (+)-CC-1065, Ent-(-)-CC-1065, and the Precise, Functional Agents (.+-.)-CPI-CDPI2, (+)-CPI-CDPI2, and (-)-CPI-CDPI2 [(.+-.)-(3bR*,4aS*)-, (+)-(3bR,4aS)-, and (-)-(3bS,4aR)-Deoxy-CC-1065]. *J. Am. Chem. Soc.* **1988**, *110* (14), 4796–4807. https://doi.org/10.1021/ja00222a043.

(5) Boger, D. L.; Ishizaki, T.; Wysocki, R. J.; Munk, S. A.; Kitos, P. A.; Suntornwat, O. Total Synthesis and Evaluation of (.+-.)-N-(Tert-Butoxycarbonyl)-CBI, (.+-.)-CBI-CDPI1, and (.+-.)-CBI-CDPI2: CC-1065 Functional Agents Incorporating the Equivalent 1,2,9,9a-Tetrahydrocyclopropa[1,2-c]Benz[1,2-e]Indol-4-One (CBI) Left-Hand Subunit. *J. Am. Chem. Soc.* **1989**, *111* (16), 6461–6463. https://doi.org/10.1021/ja00198a089.

(6) Boger, D. L.; Wysocki, R. J. Total Synthesis of (.+-.)-N-(Phenylsulfonyl)- and (.+-.)-N-(Tert-Butyloxycarbonyl)-CI, (.+-.)-CI-CDPI1, and (.+-.)-CI-CDPI2: CC-1065 Functional Analogs Incorporating the Parent 1,2,7,7a-Tetrahydrocycloprop[1,2-c]Indol-4-One (CI) Left-Hand Subunit. *J. Org. Chem.* **1989**, *54* (6), 1238–1240. https://doi.org/10.1021/jo00267a004.

(7) Boger, D. L.; Ishizaki, T. Resolution of a CBI Precursor and Incorporation into the Synthesis of (+)-Cbi, (+)-CBI-CDPI1, (+)-CBI-CDPI2: Enhanced Functional Analogs of (+)-CC-1065. A Critical Appraisal of a Proposed Relationship between Electrophile Reactivity, DNA Binding Properties, and Cytotoxic Potency. *Tet. Lett.* **1990**, *31* (6), 793–796. https://doi.org/10.1016/s0040-4039(00)94629-x.

(8) Boger, D. L.; Coleman, R. S.; Invergo, B. J.; Sakya, S. M.; Ishizaki, T.; Munk, S. A.; Zarrinmayeh, H.; Kitos, P. A.; Thompson, S. C. Synthesis and Evaluation of Aborted and Extended CC-1065 Functional Analogs: (+)- and (-)-CPI-PDE-I1, (+)- and (-)-CPI-CDPI1, and (.+-.)-, (+)-, and (-)-CPI-CDPI3. Preparation of Key Partial Structures and Definition of an Additional Functional Role of the CC-1065 Central and Right-Hand Subunits. *J. Am. Chem. Soc.* **1990**, *112* (12), 4623–4632. https://doi.org/10.1021/ja00168a002.

(9) Boger, D. L.; Ishizaki, T.; Kitos, P. A.; Suntornwat, O. Synthesis of N-(Tert-Butyloxycarbonyl)-CBI, CBI, CBI-CDPI1, and CBI-CDPI2: Enhanced Functional Analogs of CC-1065 Incorporating the 1,2,9,9a-Tetrahydrocyclopropa[c]Benz[e]Indol-4-One (CBI) Left-Hand Subunit. *J. Org. Chem.* **1990**, *55* (23), 5823–5832. https://doi.org/10.1021/jo00310a013.

(10) Boger, D. L.; Ishizaki, T.; Zarrinmayeh, H.; Munk, S. A.; Kitos, P. A.; Suntornwat, O. Duocarmycin-Pyrindamycin DNA Alkylation Properties and Identification, Synthesis, and Evaluation of Agents Incorporating the Pharmacophore of the Duocarmycin-Pyrindamycin Alkylation Subunit. Identification of the CC-1065 Duocarmycin Common Pharmacophore. *J. Am. Chem. Soc.* **1990**, *112* (24), 8961–8971. https://doi.org/10.1021/ja00180a048.

(11) Boger, D. L.; Ishizaki, T.; Zarrinmayeh, H.; Kitos, P. A.; Suntornwat, O. Synthesis and Preliminary Evaluation of Agents Incorporating the Pharmacophore of the Duocarmycin/Pyrindamycin Alkylation Subunit: Identification of the CC-1065/Duocarmycin Common Pharmacophore. *J. Org. Chem.* **1990**, *55* (15), 4499–4502. https://doi.org/10.1021/jo00302a002.

(12) Boger, D. L.; Wysocki, R. J.; Ishizaki, T. Synthesis of N-(Phenylsulfonyl)-CI, N-((Tert-Butyloxy)Carbonyl)-CI, CI-CDPI1, and CI-CDPI2: CC-1065 Functional Analog Incorporating the Parent 1,2,7,71-Tetrahydrocycloprop[1,2-c]Indol-4-One (CI) Left-Hand Subunit. *J. Am. Chem. Soc.* **1990**, *112* (13), 5230–5240. https://doi.org/10.1021/ja00169a034.

(13) Boger, D. L.; Ishizaki, T.; Sakya, S. M.; Munk, S. A. Synthesis and Preliminary Evaluation of (+)-CBI-Indole2; An Enhanced Functional Analog of (+)-CC-1065. *Bioorg. Med. Chem. Lett.* **1991**, *1* (2), 115–120. https://doi.org/10.1016/s0960-894x(00)80243-7.

(14) Boger, D. L.; Ishizaki, T.; Zarrinmayeh, H.; Kitos, P. A.; Suntornwat, O. A Potent, Simple Derivative of an Analog of the CC-1065 Alkylation Subunit. *Bioorg. Med. Chem. Lett.* **1991**, *1* (1), 55–58. https://doi.org/10.1016/S0960-894X(01)81090-8.

(15) Boger, D. L.; Munk, S. A.; Ishizaki, T. (+)-CC-1065 DNA Alkylation: Observation of an Unexpected Relationship between Cyclopropane Electrophile Reactivity and the Intensity of DNA Alkylation. *J. Am. Chem. Soc.* **1991**, *113* (7), 2779–2780. https://doi.org/10.1021/ja00007a077.

(16) Boger, D. L.; Munk, S. A.; Zarrinmayeh, H. (+)-CC-1065 DNA Alkylation: Key Studies Demonstrating a Noncovalent Binding Selectivity Contribution to the Alkylation Selectivity. *J. Am. Chem. Soc.* **1991**, *113* (10), 3980–3983. https://doi.org/10.1021/ja00010a046.

(17) Aristoff, P. A.; Johnson, P. D. Synthesis of CBI-PDE-I-Dimer, the Benzannelated Analog of CC-1065. *J. Org. Chem.* **1992**, *57* (23), 6234–6239. https://doi.org/10.1021/jo00049a035.

(18) Boger, D. L.; Sakya, S. M. CC-1065 Partial Structures: Enhancement of Noncovalent Affinity for DNA Minor Groove Binding through Introduction of Stabilizing Electrostatic Interactions. *J. Org. Chem.* **1992**, *57* (4), 1277–1284. https://doi.org/10.1021/jo00030a042.

(19) Boger, D. L.; Yun, W.; Teegarden, B. R. An Improved Synthesis of 1,2,9,9a-Tetrahydrocyclopropa[c]Benz[e]Indol-4-One (CBI): A Simplified Analog of the CC-1065 Alkylation Subunit. *J. Org. Chem.* **1992**, *57* (10), 2873–2876. https://doi.org/10.1021/jo00036a023.

(20) Aristoff, P. A.; Johnson, P. D.; Sun, D.; Hurley, L. H. Synthesis and Biochemical Evaluation of the CBI-PDE-I-Dimer, a Benzannelated Analog of (+)-CC-1065 That Also Produces Delayed Toxicity in Mice. *J. Med. Chem.* **1993**, *36* (14), 1956–1963. https://doi.org/10.1021/jm00066a004.

(21) Boger, D. L.; Johnson, D. S.; Palanki, M. S. S.; Kitos, P. A.; Chang, J.; Dowell, P. Evaluation of Functional Analogs of CC-1065 and the Duocarmycins Incorporating the Cross-Linking 9a-Chloromethyl-1,2,9,9a-

Tetrahydrocyclopropa[c]Benz[e]Indol-4-One (C2BI) Alkylation Subunit. *Bioorg. Med. Chem.* **1993**, *1* (1), 27–38. https://doi.org/10.1016/S0968-0896(00)82100-8.

(22) Wang, Y.; Gupta, R.; Huang, L.; Lown, J. W. CC-1065 Functional Analogs Possessing Different Electron-Withdrawing Substituents and Leaving Groups: Synthesis, Kinetics, and Sequence Specificity of Reaction with DNA and Biological Evaluation. *J. Med. Chem.* **1993**, *36* (26), 4172–4182. https://doi.org/10.1021/jm00078a005.

(23) Boger, D. L.; Mesini, P. Design, Synthesis, and Evaluation of CC-1065 and Duocarmycin Analogs Incorporating the 2,3,10,10a-Tetrahydro-1H-Cyclopropa[d]Benzo[f]Quinol-5-One (CBQ) Alkylation Subunit: Identification and Structural Origin of Subtle Stereoelectronic Features That Govern Reactivity and Regioselectivity. *J. Am. Chem. Soc.* **1994**, *116* (25), 11335–11348. https://doi.org/10.1021/ja00104a013.

(24) Boger, D. L.; Mesini, P.; Tarby, C. M. Chemical and Structural Comparison of N-BOC-CBQ and N-BOC-CBI: Identification and Structural Origin of an Unappreciated but Productive Stability of the CC-1065 and Duocarmycin SA Alkylation Subunits. *J. Am. Chem. Soc.* **1994**, *116* (14), 6461–6462. https://doi.org/10.1021/ja00093a067.

(25) Boger, D. L.; Nishi, T.; Teegarden, B. R. P-Quinonemethide Analog of the CC-1065 and Duocarmycin Alkylation Subunits. *J. Org. Chem.* **1994**, *59* (17), 4943–4949. https://doi.org/10.1021/jo00096a043.

(26) Boger, D. L.; Yun, W. CBI-TMI: Synthesis and Evaluation of a Key Analog of the Duocarmycins. Validation of a Direct Relationship between Chemical Solvolytic Stability and Cytotoxic Potency and Confirmation of the Structural Features Responsible for the Distinguishing Behavior of Enantiomeric Pairs of Agents. *J. Am. Chem. Soc.* **1994**, *116* (18), 7996–8006. https://doi.org/10.1021/ja00097a006.

(27) Mohamadi, F.; Spees, M. M.; Staten, G. S.; Marder, P.; Kipka, J. K.; Johnson, D. A.; Boger, D. L.; Zarrinmayeh, H. Total Synthesis and Biological Properties of Novel Antineoplastic (Chloromethyl)Furanoindolines: An Asymmetric Hydroboration Mediated Synthesis of the Alkylation Subunits. *J. Med. Chem.* **1994**, *37* (2), 232–239. https://doi.org/10.1021/jm00028a005.

(28) Boger, D. L.; Johnson, D. S. CC-1065 and the Duocarmycins: Unraveling the Keys to a New Class of Naturally Derived DNA Alkylating Agents. *Proc. Natl. Acad. Sci. USA* **1995**, *92* (9), 3642–3649. https://doi.org/10.1073/pnas.92.9.3642.

(29) Boger, D. L.; Yun, W.; Cai, H.; Han, N. CBI-CDPBO and CBI-CDPBII: CC-1065 Analogs Containing Deep-Seated Modifications in the DNA Binding Subunit. *Bioorg. Med. Chem.* **1995**, *3* (6), 761–767. https://doi.org/10.1016/0968-0896(95)00066-p.

(30) Boger, D. L.; McKie, J. A. An Efficient Synthesis of 1,2,9,9a-Tetrahydrocyclopropa[c]Benz[e]Indol-4-One CBI: An Enhanced and Simplified Analog of the CC-1065 and Duocarmycin Alkylation Subunits. *J. Org. Chem.* **1995**, *60* (5), 1271–1275. https://doi.org/10.1021/jo00110a034.

(31) Boger, D. L.; Nishi, T. Diastereoselective Dieckmann Condensation Suitable for Introduction of the Duocarmycin A C6 Center: Development of a Divergent Strategy for the Total Synthesis of Duocarmycins A and SA. *Bioorg. Med. Chem.* **1995**, *3* (1), 67–77. https://doi.org/10.1016/0968-0896(94)00147-U.

(32) Boger, D. L.; Yun, W.; Han, N. 1,2,9,9a-Tetrahydrocyclopropa[c]Benz[e]Indol-4-One (CBI) Analogs of CC-1065 and the Duocarmycins: Synthesis and Evaluation. *Bioorg. Med. Chem.* **1995**, *3* (11), 1429–1453. https://doi.org/10.1016/0968-0896(95)00130-9.

(33) Fregeau, N. L.; Wang, Y.; Pon, R. T.; Wylie, W. A.; Lown, J. W. Characterization of a CPI-Lexitropsin Conjugate-Oligonucleotide Covalent Complex by 1H NMR and Restrained Molecular Dynamics Simulation. *J. Am. Chem. Soc.* **1995**, *117* (35), 8917–8925. https://doi.org/10.1021/ja00140a004.

(34) Nagamura, S.; Kanda, Y.; Kobayashi, E.; Gomi, K.; Saito, H. Synthesis and Antitumor Activity of Duocarmycin Derivatives. *Chem. Pharm. Bull.* **1995**, *49* (9), 1530–1535.

(35) Boger, D. L.; Bollinger, B.; Johnson, D. S. Examination of the Role of the Duocarmycin SA Methoxy Substituents: Identification of the Minimum, Fully Potent DNA Binding Subunit. *Bioorg. Med. Chem. Lett.* **1996**, *6* (18), 2207–2210. https://doi.org/10.1016/0960-894X(96)00401-5.

(36) Boger, D. L.; Han, N.; Tarby, C. M.; Boyce, C. W.; Cai, H.; Jin, Q.; Kitos, P. A. Synthesis, Chemical Properties, and Preliminary Evaluation of Substituted CBI Analogs of CC-1065 and the Duocarmycins Incorporating the 7-Cyano-1,2,9,9a-Tetrahydrocyclopropa[*c*]Benz[*e*]Indol-4-One Alkylation Subunit: Hammett Quantitation of the Magnitude of Electronic Effects on Functional Reactivity. *J. Org. Chem.* **1996**, *61* (15), 4894–4912. https://doi.org/10.1021/jo9605298.

(37) Boger, D. L.; Jenkins, T. J. Synthesis, X-Ray Structure, and Properties of Fluorocyclopropane Analogs of the Duocarmycins Incorporating the 9,9-Difluoro-1,2,9,9a-Tetrahydrocyclopropa[*c*]Benzo[*e*]Indol-4-One (F_2 CBI) Alkylation Subunit. *J. Am. Chem. Soc.* **1996**, *118* (37), 8860–8870. https://doi.org/10.1021/ja961888n.

(38) Boger, D. L.; McKie, J. A.; Cai, H.; Cacciari, B.; Baraldi, P. G. Synthesis and Properties of Substituted CBI Analogs of CC-1065 and the Duocarmycins Incorporating the 7-Methoxy-1,2,9,9a-Tetrahydrocyclopropa[*c*]Benz[*e*]Indol-4-One (MCBI) Alkylation Subunit: Magnitude of Electronic Effects on the Functional Reactivity. *J. Org. Chem.* **1996**, *61* (5), 1710–1729. https://doi.org/10.1021/jo952033g.

(39) Boger, D. L.; McKie, J. A.; Han, N.; Tarby, C. M.; Riggs, H. W.; Kitos, P. A. A Hammett Correlation for CC-1065 and Duocarmycin Analogs: Magnitude of Substituent Electronic Effects on Functional Reactivity. *Bioorg. Med. Chem. Lett.* **1996**, *6* (6), 659–664. https://doi.org/10.1016/0960-894X(96)00093-5.

(40) Nagamura, S.; Kanda, Y.; Asai, A.; Kobayashi, E.; Gomi, K.; Saito, H. Wagner-Meerwein Rearrangement of Duocarmycins. *Chem. Pharm. Bull.* **1996**, *44* (5), 933–939.

(41) Tercel, M.; Denny, W. A.; Wilson, W. R. Nitrogen and Sulfur Analogues of the SecoCI Alkylating Agent: Synthesis and Cytotoxicity. *Bioorg. Med. Chem. Lett.* **1996**, *6* (22), 2735–2740. https://doi.org/10.1016/S0960-894X(96)00507-0.

(42) Atwell, G. J.; Wilson, W. R.; Denny, W. A. Synthesis and Cytotoxicity of Amino Analogues of the Potent DNA Alkylating Agent Seco-CBI-TMI. *Bioorg. Med. Chem. Lett.* **1997**, *7* (12), 1493–1496. https://doi.org/10.1021/jm990136b.

(43) Boger, D. L.; Bollinger, B.; Hertzog, D. L.; Johnson, D. S.; Cai, H.; Mesini, P.; Garbaccio, R. M.; Jin, Q.; Kitos, P. A. Reversed and Sandwiched Analogs of Duocarmycin SA: Establishment of the Origin of the Sequence-Selective Alkylation of DNA and New Insights into the Source of Catalysis. *J. Am. Chem. Soc.* **1997**, *119* (21), 4987–4998. https://doi.org/10.1021/ja9637210.

(44) Boger, D. L.; Garbaccio, R. M.; Jin, Q. Synthesis and Evaluation of CC-1065 and Duocarmycin Analogues Incorporating the Iso-CI and Iso-CBI Alkylation Subunits: Impact of Relocation of the C-4 Carbonyl. *J. Org. Chem.* **1997**, *62* (25), 8875–8891. https://doi.org/10.1021/jo971686p.

(45) Boger, D. L.; Hertzog, D. L.; Bollinger, B.; Johnson, D. S.; Cai, H.; Goldberg, J.; Turnbull, P. Duocarmycin SA Shortened, Simplified, and Extended Agents: A Systematic Examination of the Role of the DNA Binding Subunit. *J. Am. Chem. Soc.* **1997**, *119* (21), 4977–4986. https://doi.org/10.1021/ja9637208.

(46) Boger, D. L.; McKie, J. A.; Boyce, C. W. Asymmetric Synthesis of the CBI Alkylation Subunit of the CC-1065 and Duocarmycin' Analogs. *Synlett* **1997**, 515–517. https://doi.org/10.1055/s-1997-6110.

(47) Boger, D. L.; Turnbull, P. Synthesis and Evaluation of CC-1065 and Duocarmycin Analogs Incorporating the 1,2,3,4,11,11a-Hexahydrocyclopropa[*c*]Naphtho[2,1- *b*]Azepin-6-One (CNA) Alkylation Subunit: Structural Features That Govern Reactivity and Reaction Regioselectivity. *J. Org. Chem.* **1997**, *62* (17), 5849–5863. https://doi.org/10.1021/jo9707085.

(48) Muratake, H.; Hayakawa, A.; Natsume, M. A Novel Phenol-Forming Reaction for Preparation of Benzene, Furan, and Thiophene Analogs of CC-1065/Duocarmycin Pharmacophores. *Tet. Lett.* **1997**, *38* (43), 7577–7580. https://doi.org/10.1016/S0040-4039(97)01786-3.

(49) Shishido, K.; Haruna, S.; Yamamura, C.; Iitsuka, H.; Nemoto, H.; Shinohara, Y.; Shibuya, M. Synthesis and Evaluation of the Hybrid Molecules Possessing DNA-Cleaving Activity. *Bioorg. Med. Chem. Lett.* **1997**, *7* (20), 2617–2622. https://doi.org/10.1016/S0960-894X(97)10030-0.

(50) Atwell, G. J.; Tercel, M.; Boyd, M.; Wilson, W. R.; Denny, W. A. Synthesis and Cytotoxicity of 5-Amino-1-(Chloromethyl)-3-[(5,6,7-Trimethoxyindol-2-Yl)Carbonyl]-1,2- Dihydro-3 *H* -Benz[*e*]Indole (Amino- *s Eco* -CBI-TMI) and Related 5-Alkylamino Analogues: New DNA Minor Groove Alkylating Agents. *J. Org. Chem.* **1998**, *63* (25), 9414–9420. https://doi.org/10.1021/jo981395w.

(51) Boger, D. L.; Boyce, C. W.; Garbaccio, R. M.; Searcey, M. Synthesis of CC-1065 and Duocarmycin Analogs via Intramolecular Aryl Radical Cyclization of a Tethered Vinyl Chloride. *Tet. Lett.* **1998**, *39* (16), 2227–2230. https://doi.org/10.1016/S0040-4039(98)00232-9.

(52) Boger, D. L.; Santillán, A.; Searcey, M.; Jin, Q. Critical Role of the Linking Amide in CC-1065 and the Duocarmycins: Implications on the Source of DNA Alkylation Catalysis. *J. Am. Chem. Soc.* **1998**, *120* (45), 11554–11557. https://doi.org/10.1021/ja9818093.

(53) Boger, D. L.; Turnbull, P. Synthesis and Evaluation of a Carbocyclic Analogue of the CC-1065 and Duocarmycin Alkylation Subunits: Role of the Vinylogous Amide and Implications on DNA Alkylation Catalysis. *J. Org. Chem.* **1998**, *63* (22), 8004–8011. https://doi.org/10.1021/jo981698q.

(54) Fukuda, Y.; Furuta, H.; Kusama, Y.; Ebisu, H.; Oomori, Y.; Terashima, S. The Novel Cyclopropapyrroloindole(CPI) Bisalkylators Bearing Methoxycarbonyl and Trifluoromethyl Groups. *Bioorg. Med. Chem. Lett.* **1998**, *8* (11), 1387–1390. https://doi.org/10.1016/S0960-894X(98)00235-2.

(55) Fukuda, Y.; Seto, S.; Furuta, H.; Ebisu, H.; Oomori, Y.; Terashima, S. The Novel Cyclopropapyrroloindole(CPI) Bisalkylators Bearing 3,3′-(1,4-Phenylene)Diacryloyl Group as a Linker. *Bioorg. Med. Chem. Lett.* **1998**, *8* (15), 2003–2004. https://doi.org/10.1016/S0960-894X(98)00346-1.

(56) Nagamura, S.; Saito, H. Antitumor Antibiotics: Duocarmycins. *Chem. Heterocycl. Comp.* **1998**, *34* (12), 1386–1405. https://doi.org/10.1007/BF02317808.

(57) Tercel, M.; Denny, W. A. Synthesis of Nitrogen and Sulfur Analogues of the Seco-CI Alkylating Agent. *J. Chem. Soc. Perkin Trans. 1* **1998**, No. 3, 509–520. https://doi.org/10.1039/a706165j.

(58) Amishiro, N.; Nagamura, S.; Kobayashi, E.; Okamoto, A.; Gomi, K.; Saito, H. Synthesis and Antitumor Activity of Duocarmycin Derivatives: A-Ring Pyrrole Compounds Bearing 5-Membered Hereoarylacryloyl Groups. *Chem. Pharm. Bull.* **1999**, *47* (10), 1393–1403.

(59) Amishiro, N.; Okamoto, A.; Murakata, C.; Tamaoki, T.; Okabe, M.; Saito, H. Synthesis and Antitumor Activity of Duocarmycin Derivatives: Modification of Segment-A of A-Ring Pyrrole Compounds. *J. Med. Chem.* **1999**, *42* (15), 2946–2960. https://doi.org/10.1021/jm990094r.

(60) Atwell, G. J.; Milbank, J. J. B.; Wilson, W. R.; Hogg, A.; Denny, W. A. 5-Amino-1-(Chloromethyl)-1,2-Dihydro-3 *H* -Benz[*e*]Indoles: Relationships between Structure and Cytotoxicity for Analogues Bearing Different DNA Minor Groove Binding Subunits. *J. Med. Chem.* **1999**, *42* (17), 3400–3411. https://doi.org/10.1021/jm990136b.

(61) Baraldi, P. G.; Cacciari, B.; Boyce, C. W.; Boger, D. L. Resolution of a CPzI Precursor, Synthesis and Biological Evaluation of (+) and (-)-N-Boc-CPzI: A Further Validation of the Relationship between Chemical Solvolytic Stability and Cytotoxicity. *Bioorg. Med. Chem. Lett.* **1999**, *9* (21), 3087–3092. https://doi.org/10.1016/s0960-894x(99)00533-8.

(62) Boger, D. L. CBI Prodrug Analogs of CC-1065 and the Duocarmycins. *Synthesis* **1999**, *1999* (S1), 1505–1509. https://doi.org/10.1055/s-1999-3658.

(63) Boger, D. L. Iso-CBI and Iso-CI Analogs of CC-1065 and the Duocarmycins. WO 99/19298, April 22, 1999.

(64) Boger, D. L.; Garbaccio, R. M. A Novel Class of CC-1065 and Duocarmycin Analogues Subject to Mitomycin-Related Reductive Activation. *J. Org. Chem.* **1999**, *64* (22), 8350–8362. https://doi.org/10.1021/jo991301y.

(65) Boger, D. L.; Garbaccio, R. M. Shape-Dependent Catalysis: Insights into the Source of Catalysis for the CC-1065 and Duocarmycin DNA Alkylation Reaction. *Acc. Chem. Res.* **1999**, *32* (12), 1043–1052. https://doi.org/10.1021/ar9800946.

(66) Fukuda, Y.; Furuta, H.; Kusama, Y.; Ebisu, H.; Oomori, Y.; Terashima, S. Novel Cyclopropapyrroloindole Derivative (AT-3510) Bearing Methoxycarbonyl and Trifluoromethyl Groups. *J. Med. Chem.* **1999**, *42* (8), 1448–1458. https://doi.org/10.1021/jm980668c.

(67) Jia, G.; Iida, H.; William Lown, J. Synthesis of an Unsymmetrical Bis-Lexitropsin-1,2,9,9a–Tetrahydrocyclo-Propa[c]Benzo[e]Indol-4-One (CBI) Conjugate. *Chem. Commun.* **1999**, No. 2, 119–120. https://doi.org/10.1039/a807884j.

(68) Milbank, J. B. J.; Tercel, M.; Atwell, G. J.; Wilson, W. R.; Hogg, A.; Denny, W. A. Synthesis of 1-Substituted 3-(Chloromethyl)-6-Aminoindoline (6-Amino- *Seco* -CI) DNA Minor Groove Alkylating Agents and Structure−Activity Relationships for Their Cytotoxicity. *J. Med. Chem.* **1999**, *42* (4), 649–658. https://doi.org/10.1021/jm980545s.

(69) Tao, Z.-F.; Fujiwara, T.; Saito, I.; Sugiyama, H. Rational Design of Sequence-Specific DNA Alkylating Agents Based on Duocarmycin A and Pyrrole−Imidazole Hairpin Polyamides. *J. Am. Chem. Soc.* **1999**, *121* (21), 4961–4967. https://doi.org/10.1021/ja983398w.

(70) Tao, Z.-F.; Fujiwara, T.; Saito, I.; Sugiyama, H. Sequence-Specific DNA Alkylation by Hybrid Molecules between Segment A of Duocarmycin A and Pyrrole/Imidazole Diamide. *Angew. Chem. Int. Ed.* **1999**, *38* (5), 650–653. https://doi.org/10.1002/(SICI)1521-3773(19990301)38:5<650::AID-ANIE650>3.0.CO;2-O.

(71) Amishiro, N.; Nagamura, S.; Kobayashi, E.; Okamoto, A.; Gomi, K.; Okabe, M.; Saito, H. Synthesis and Antitumor Activity of Duocarmycin Derivatives: A-Ring Pyrrole Compounds Bearing ß-(5H,6H,7H-Trimethoxy-2H-Indolyl)Acryloyl Group. *Bioorg. Med. Chem.* **2000**, *8*, 1637–1643. https://doi.org/10.1016/s0968-0896(00)00086-9.

(72) Amishiro, N.; Nagamura, S.; Murakata, C.; Okamoto, A.; Kobayashi, E.; Asada, M.; Gomi, K.; Tamaoki, T.; Okabe, M.; Yamaguchi, N.; Yamaguchi, K.; Saito, H. Synthesis and Antitumor Activity of Duocarmycin Derivatives: Modification at C-8 Position of A-Ring Pyrrole Compounds Bearing the Simplified DNA-Binding Groups. *Bioorg. Med. Chem.* **2000**, *8* (2), 381–391. https://doi.org/10.1016/s0968-0896(99)00293-x.

(73) Amishiro, N.; Okamoto, A.; Okabe, M.; Saito, H. Synthesis and Antitumor Activity of Duocarmycin Derivatives: Modification at the C-7 Position of Segment-A of A-Ring Pyrrole Compounds. *Bioorg. Med. Chem.* **2000**, *8*, 1195–1201.

(74) Boger, D. L.; Boyce, C. W. Selective Metal Cation Activation of a DNA Alkylating Agent: Synthesis and Evaluation of Methyl 1,2,9,9a-Tetrahydrocyclopropa[*c*]Pyrido[3,2- *e*]Indol-4-One-7-Carboxylate (CPyI). *J. Org. Chem.* **2000**, *65* (13), 4088–4100. https://doi.org/10.1021/jo000177b.

(75) Boger, D. L.; Fink, B. E.; Hedrick, M. P. A New Class of Highly Cytotoxic Diketopiperazines. *Bioorg. Med. Chem. Lett.* **2000**, *10* (10), 1019–1020. https://doi.org/10.1016/S0960-894X(00)00152-9.

(76) Boger, D. L.; Santillán, A.; Searcey, M.; Brunette, S. R.; Wolkenberg, S. E.; Hedrick, M. P.; Jin, Q. Synthesis and Evaluation of 1,2,8,8a-Tetrahydrocyclopropa[*c*]Pyrrolo[3,2- *e*]Indol-4(5 *H*)-One, the Parent Alkylation Subunit of CC-1065 and the Duocarmycins: Impact of the Alkylation Subunit Substituents and Its Implications for DNA Alkylation Catalysis. *J. Org. Chem.* **2000**, *65* (13), 4101–4111. https://doi.org/10.1021/jo000297j.

(77) Boger, D. L.; Searcey, M.; Tse, W. C.; Jin, Q. Bifunctional Alkylating Agents Derived from Duocarmycin SA: Potent Antitumor Activity with Altered Sequence Selectivity. *Bioorg. Med. Chem. Lett.* **2000**, *10* (5), 495–498. https://doi.org/10.1016/S0960-894X(00)00042-1.

(78) Boger, D. L.; Wolkenberg, S. E.; Boyce, C. W. A New Method of in Situ Activation for a Novel Class of DNA Alkylating Agents: Tunable Metal Cation Complexation and Activation. *J. Am. Chem. Soc.* **2000**, *122* (26), 6325–6326. https://doi.org/10.1021/ja000653f.

(79) Chang, A. Y.; Dervan, P. B. Strand Selective Cleavage of DNA by Diastereomers of Hairpin Polyamide- *Seco* -CBI Conjugates. *J. Am. Chem. Soc.* **2000**, *122* (20), 4856–4864. https://doi.org/10.1021/ja994345x.

(80) Fukuda, Y.; Terashima, S. Intermediates for the Preparation of Duocarmycin SA and Derivatives Thereof, and Process for the Production of the Intermediates. US006066742A, May 23, 2000.

(81) Jia, G.; Iida, H.; Lown, J. W. Solid-Phase Synthesis of 1-Chloromethyl-1,2-Dihydro-3 *H* -Benz[*e*]Indole (*Seco* -CBI) and a Polyamide Conjugate. *Synlett* **2000**, *2000* (05), 0603–0606. https://doi.org/10.1055/s-2000-6631.

(82) Jia, G.; Lown, J. W. Design, Synthesis and Cytotoxicity Evaluation of 1-Chloromethyl-5-Hydroxy-1,2-Dihydro-3H -Benz[e]Indole (Seco-CBI) Dimers. *Bioorg. Med. Chem.* **2000**, *8* (7), 1607–1617. https://doi.org/10.1016/S0968-0896(00)00088-2.

(83) Muratake, H.; Hayakawa, A.; Natsume, M. Preparation of Benzene, Furan, and Thiophene Analogs of Duocarmycin SA Employing a Newly-Devised Phenol-Forming Reaction. *Chem. Pharm. Bull.* **2000**, *48* (10), 1558–1566. https://doi.org/10.1248/cpb.48.1558.

(84) Tao, Z.-F.; Saito, I.; Sugiyama, H. Highly Cooperative DNA Dialkylation by the Homodimer of Imidazole−Pyrrole Diamide−CPI Conjugate with Vinyl Linker. *J. Am. Chem. Soc.* **2000**, *122* (8), 1602–1608. https://doi.org/10.1021/ja9926212.

(85) Wang, Y.; Yuan, H.; Ye, W.; Wright, S. C.; Wang, H.; Larrick, J. W. Synthesis and Preliminary Biological Evaluations of CC-1065 Analogues: Effects of Different Linkers and Terminal Amides on Biological Activity. *J. Med. Chem.* **2000**, *43* (8), 1541–1549. https://doi.org/10.1021/jm990514c.

(86) Bando, T.; Iida, H.; Saito, I.; Sugiyama, H. Sequence-Specific DNA Interstrand Cross-Linking by Imidazole−Pyrrole CPI Conjugate. *J. Am. Chem. Soc.* **2001**, *123* (21), 5158–5159. https://doi.org/10.1021/ja003660c.

(87) Baraldi, P. G.; Balboni, G.; Pavani, M. G.; Spalluto, G.; Tabrizi, M. A.; Clercq, E. D.; Balzarini, J.; Bando, T.; Sugiyama, H.; Romagnoli, R. Design, Synthesis, DNA Binding, and Biological Evaluation of Water-Soluble Hybrid Molecules Containing Two Pyrazole Analogues of the Alkylating Cyclopropylpyrroloindole (CPI) Subunit of the Antitumor Agent CC-1065 and Polypyrrole Minor Groove Binders. *J. Med. Chem.* **2001**, *44* (16), 2536–2543. https://doi.org/10.1021/jm0108404.

(88) Boger, D. L.; Brunette, S. R.; Garbaccio, R. M. Synthesis and Evaluation of a Series of C3-Substituted CBI Analogues of CC-1065 and the Duocarmycins. *J. Org. Chem.* **2001**, *66* (15), 5163–5173. https://doi.org/10.1021/jo010309g.

(89) Boger, D. L.; Hughes, T. V.; Hedrick, M. P. Synthesis, Chemical Properties, and Biological Evaluation of CC-1065 and Duocarmycin Analogues Incorporating the 5-Methoxycarbonyl-1,2,9,9a-Tetrahydrocyclopropa[*c*]Benz[*e*]Indol-4-One Alkylation Subunit. *J. Org. Chem.* **2001**, *66* (7), 2207–2216. https://doi.org/10.1021/jo001772g.

(90) Boger, D. L.; Schmitt, H. W.; Fink, B. E.; Hedrick, M. P. Parallel Synthesis and Evaluation of 132 (+)-1,2,9,9a-Tetrahydrocyclopropa[*c*]Benz[*e*]Indol-4-One (CBI) Analogues of CC-1065 and the Duocarmycins Defining the Contribution of the DNA-Binding Domain. *J. Org. Chem.* **2001**, *66* (20), 6654–6661. https://doi.org/10.1021/jo010454u.

(91) Boger, D. L.; Stauffer, F.; Hedrick, M. P. Substituent Effects within the DNA Binding Subunit of CBI Analogues of the Duocarmycins and CC-1065. *Bioorg. Med. Chem. Lett.* **2001**, *11* (15), 2021–2024. https://doi.org/10.1016/S0960-894X(01)00372-9.

(92) Castedo, L.; Delamano, J.; Enjo, J.; Fernández, J.; Grávalos, D. G.; Leis, R.; López, C.; Marcos, C. F.; Ríos, A.; Tojo, G. Derivatives of Methyl 5-Methyl-4-Oxo-1,2,4,5,8,8a- Hexahydrocyclopropa[*c*]-Pyrrolo[3,2- *e*

]Indole-7- Carboxylate: A Case of Inverse Electronic Effects on the Reactivity of CC-1065 Derivatives. *J. Am. Chem. Soc.* **2001**, *123* (21), 5102–5103. https://doi.org/10.1021/ja005704n.

(93) Ellis, D. A.; Wolkenberg, S. E.; Boger, D. L. Metal Cation Complexation and Activation of Reversed CPyI Analogues of CC-1065 and Duocarmycin SA: Partitioning the Effects of Binding and Catalysis. *J. Am. Chem. Soc.* **2001**, *123* (38), 9299–9306. https://doi.org/10.1021/ja010769r.

(94) Fukuda, Y.; Seto, S.; Furuta, H.; Ebisu, H.; Oomori, Y.; Terashima, S. Novel Seco Cyclopropa[c]Pyrrolo[3,2-e]Indole Bisalkylaotrs Bearing a 3,3'-Arylenebisacryloyl Group as a Linker. *J. Med. Chem.* **2001**, *44*, 1396–1406. https://doi.org/10.1021/jm000107x.

(95) Jennings, S. A.; Toth, J. L.; Roller, S. G.; Brooks, N.; O'Hare, C.; Kiakos, K.; Hartley, J. A.; Burke, P. J.; Lee, M. Efficient Synthesis of (+-)-Seco-Cyclopropaneindoline Analogs of CC-1065. *Heterocyc. Commun.* **2001**, *7* (1). https://doi.org/10.1515/HC.2001.7.1.7.

(96) Bando, T.; Narita, A.; Saito, I.; Sugiyama, H. Molecular Design of a Pyrrole–Imidazole Hairpin Polyamides for Effective DNA Alkylation. *Chem. Eur. J.* **2002**, *8* (20), 4781–4790. https://doi.org/10.1002/1521-3765(20021018)8:20<4781::AID-CHEM4781>3.0.CO;2-J.

(97) Boger, D. L. Synthesis of CC-1065/Duocarmycin Analogs. US 2002/0082424 A1, 2002.

(98) Howard, T. T.; Lingerfelt, B. M.; Purnell, B. L.; Scott, A. E.; Price, C. A.; Townes, H. M.; McNulty, L.; Handl, H. L.; Summerville, K.; Hudson, S. J.; Bowen, J. P.; Kiakos, K.; Hartley, J. A.; Lee, M. Novel Furano Analogues of Duocarmycin C1 and C2: Design, Synthesis, and Biological Evaluation of Seco-Iso-Cyclopropylfurano[2,3-e]Indoline (Seco-Iso-CFI) and Seco-Cyclopropyltetrahydrofurano[2,3-f]Quinoline (Seco-CFQ) Analogues. *Bioorg. Med. Chem.* **2002**, *10* (9), 2941–2952. https://doi.org/10.1016/S0968-0896(02)00157-8.

(99) Kumar, R.; Lown, J. W. Design and Synthesis of Bis 1-Chloromethyl-5-Hydroxy-1,2- Dihydro-3 *H* -Benz[*e*]Indole (*s Eco* -CBI)-Pyrrole Polyamide Conjugates. *Org. Lett.* **2002**, *4* (11), 1851–1854. https://doi.org/10.1021/ol020047k.

(100) Toth, J. L.; Price, C. A.; Madsen, E. C.; Handl, H. L.; Hudson, S. J.; Hubbard, III, R. B.; Bowen, J. P.; Kiakos, K.; Hartley, J. A.; Lee, M. Sequence Selective Recognition of DNA by Hairpin Conjugates of a Racemic Seco-Cyclopropaneindoline-2-Benzofurancarboxamide and Polyamides. *Bioorg. Med. Chem. Lett.* **2002**, *12*, 2245–2248. https://doi.org/10.1016/s0960-894x(02)00341-4.

(101) Yang, S.; Denny, W. A. A New Short Synthesis of 3-Substituted 5-Amino-1-(Chloromethyl)-1,2-Dihydro-3 *H* -Benzo[*e*]Indoles (Amino-CBIs). *J. Org. Chem.* **2002**, *67* (25), 8958–8961. https://doi.org/10.1021/jo0263115.

(102) Bando, T.; Iida, H.; Tao, Z.-F.; Narita, A.; Fukuda, N.; Yamori, T.; Sugiyama, H. Sequence Specificity, Reactivity, and Antitumor Activity of DNA-Alkylating Pyrrole-Imidazole Diamides. *Chem. & Biol.* **2003**, *10* (8), 751–758. https://doi.org/10.1016/S1074-5521(03)00160-1.

(103) Bando, T.; Narita, A.; Saito, I.; Sugiyama, H. Highly Efficient Sequence-Specific DNA Interstrand Cross-Linking by Pyrrole/Imidazole CPI Conjugates. *J. Am. Chem. Soc.* **2003**, *125* (12), 3471–3485. https://doi.org/10.1021/ja028459b.

(104) Kumar, R.; Lown, J. W. Synthesis and Cytotoxicity Evaluation of Novel C7–C7, C7–N3 and N3–N3 Dimers of 1-Chloromethyl-5-Hydroxy-1,2-Dihydro-3H-Benzo[e]Indole (Seco-CBI) with Pyrrole and Imidazole Polyamide Conjugates. *Org. Biomol. Chem.* **2003**, *1* (15), 2630–2647. https://doi.org/10.1039/B303650M.

(105) Lee, M. Compositions and Methods of the Use Thereof Achiral Analogues of CC-1065 and the Duocarmycins. US20030073731A1, April 17, 2003.

(106) Narita, A.; Bando, T.; Sugiyama, H. Molecular Design of Hairpin Pyrrole-Imidazole Polyamides Possessing Sequence Specific DNA Alkylating Moiety. *Nucl. Acid. Symp. Ser.* **2003**, *3* (1), 119–120. https://doi.org/10.1093/nass/3.1.119.

(107) Oyoshi, T.; Kawakami, W.; Narita, A.; Bando, T.; Sugiyama, H. Inhibition of Transcription at a Coding Sequence by Alkylating Polyamide. *J. Am. Chem. Soc.* **2003**, *125* (16), 4752–4754. https://doi.org/10.1021/ja029196o.

(108) Parrish, J. P.; Kastrinsky, D. B.; Hwang, I.; Boger, D. L. Synthesis and Evaluation of Duocarmycin and CC-1065 Analogues Incorporating the 1,2,9,9a-Tetrahydrocyclopropa[*c*]Benz[*e*]-3-Azaindol-4-One (CBA) Alkylation Subunit. *J. Org. Chem.* **2003**, *68* (23), 8984–8990. https://doi.org/10.1021/jo035119f.

(109) Parrish, J. P.; Kastrinsky, D. B.; Stauffer, F.; Hedrick, M. P.; Hwang, I.; Boger, D. L. Establishment of Substituent Effects in the DNA Binding Subunit of CBI Analogues of the Duocarmycins and CC-1065. *Bioorg. Med. Chem.* **2003**, *11* (17), 3815–3838. https://doi.org/10.1016/S0968-0896(03)00194-9.

(110) Tietze, L. F.; Looft, J.; Feuerstein, T. Synthesis of Ring Size Seco-Analogs of the Antitumor Antibiotic CC-1065 by Two Consecutive Transition Metal-Initiated Transformations. *Eur. J. Org. Chem.* **2003**, *2003* (15), 2749–2755. https://doi.org/10.1002/ejoc.200300077.

(111) Wang, Y.; Li, L.; Ye, W.; Tian, Z.; Jiang, W.; Wang, H.; Wright, S. C.; Larrick, J. W. CC-1065 Analogues Bearing Different DNA-Binding Subunits: Synthesis, Antitumor Activity, and Preliminary Toxicity Study. *J. Med. Chem.* **2003**, *46*, 634–637. https://doi.org/10.1021/jm0203433.

(112) Bando, T.; Narita, A.; Asada, K.; Ayame, H.; Sugiyama, H. Enantioselective DNA Alkylation by a Pyrrole−Imidazole *S* -CBI Conjugate. *J. Am. Chem. Soc.* **2004**, *126* (29), 8948–8955. https://doi.org/10.1021/ja049398f.

(113) Bando, T.; Narita, A.; Iwai, A.; Kihara, K.; Sugiyama, H. C−H to N Substitution Dramatically Alters the Sequence-Specific DNA Alkylation, Cytotoxicity, and Expression of Human Cancer Cell Lines. *J. Am. Chem. Soc.* **2004**, *126* (11), 3406–3407. https://doi.org/10.1021/ja0387103.

(114) Boger, D. L. CBI Analogues of the Duocarmycins and CC-1065. WO 2004/101767 A2, 2004.

(115) Cimino, P.; Improta, R.; Bifulco, G.; Riccio, R.; Gomez-Paloma, L.; Barone, V. Nucleophilic Cyclopropane Ring Opening in Duocarmycin SA Derivatives by Methanol under Acid Conditions: A Quantum Mechanical Study in the Gas-Phase and in Solution. *J. Org. Chem.* **2004**, *69* (8), 2816–2824. https://doi.org/10.1021/jo0303517.

(116) Ham, Y.-W.; Boger, D. L. A Powerful Selection Assay for Mixture Libraries of DNA Alkylating Agents. *J. Am. Chem. Soc.* **2004**, *126* (30), 9194–9195. https://doi.org/10.1021/ja0477930.

(117) Hiroya, K.; Matsumoto, S.; Sakamoto, T. New Synthetic Method for Indole-2-Carboxylate and Its

Application to the Total Synthesis of Duocarmycin SA. *Org. Lett.* **2004**, *6* (17), 2953–2956. https://doi.org/10.1021/ol0489548.

(118) Kastrinsky, D. B.; Boger, D. L. Effective Asymmetric Synthesis of 1,2,9,9a-Tetrahydrocyclopropa[*c*]Benzo[*e*]Indol-4-One (CBI). *J. Org. Chem.* **2004**, *69* (7), 2284–2289. https://doi.org/10.1021/jo035465x.

(119) Kupchinsky, S.; Centioni, S.; Howard, T.; Trzupek, J.; Roller, S.; Carnahan, V.; Townes, H.; Purnell, B.; Price, C.; Handl, H.; Summerville, K.; Johnson, K.; Toth, J.; Hudson, S.; Kiakos, K.; Hartley, J. A.; Lee, M. A Novel Class of Achiral Seco-Analogs of CC-1065 and the Duocarmycins: Design, Synthesis, DNA Binding, and Anticancer Properties. *Bioorg. Med. Chem.* **2004**, *12* (23), 6221–6236. https://doi.org/10.1016/j.bmc.2004.08.051.

(120) Pati, H.; Forrest, L.; Townes, H.; Lingerfelt, B.; McNulty, L.; Lee, M. Unexpected Syntheses of Seco-Cyclopropyltetrahydroquinolines >From a Radical 5-Exo-Trig Cyclization Reaction: Analogs of CC-1065 and the Duocarmycins. *Molecules* **2004**, *9* (3), 125–133. https://doi.org/10.3390/90300125.

(121) Sugiyama, H.; Bando, T.; Saito, I. Novel Hairpin Polyamide. EP1491534A1, December 29, 2004.

(122) Tichenor, M. S.; Kastrinsky, D. B.; Boger, D. L. Total Synthesis, Structure Revision, and Absolute Configuration of (+)-Yatakemycin. *J. Am. Chem. Soc.* **2004**, *126* (27), 8396–8398. https://doi.org/10.1021/ja0472735.

(123) Al-Said, N.; Shawakfeh, K.; Abdullah, W. Cyclization of Free Radicals at the C-7 Position of Ethyl Indole–2-Carboxylate Derivatives: An Entry to a New Class of Duocarmycin Analogues. *Molecules* **2005**, *10* (12), 1446–1457. https://doi.org/10.3390/10121446.

(124) Bando, T.; Narita, A.; Sasaki, S.; Sugiyama, H. Specific Adenine Alkylation by Pyrrole−Imidazole CBI Conjugates. *J. Am. Chem. Soc.* **2005**, *127* (40), 13890–13895. https://doi.org/10.1021/ja052412j.

(125) Boger, D. L. CBI Analogues of CC-1065 and the Duocarmycins. US 20050014700A1, January 20, 2005.

(126) Daniell, K.; Stewart, M.; Madsen, E.; Le, M.; Handl, H.; Brooks, N.; Kiakos, K.; Hartley, J. A.; Lee, M. Design, Synthesis, and Biological Evaluation of Achiral Analogs of Duocarmycin SA. *Bioorg. Med. Chem. Lett.* **2005**, *15* (1), 177–180. https://doi.org/10.1016/j.bmcl.2004.10.021.

(127) Nelson, S. M.; Ferguson, L. R.; Denny, W. A. Demonstration by Real-Time Polymerase Chain Reaction That Cellular DNA Alkylation by Novel Aminoindoline Compounds Affects Expression of the Protooncogene c-Myc. *Chem. Res. Toxicol.* **2005**, *18* (2), 239–248. https://doi.org/10.1021/tx049852t.

(128) Price, C. A.; Lingerfelt, B. M.; Handl, H. L.; Kiakos, K.; Hartley, J. A.; Lee, M. Sequence Specific Recognition of DNA by Tailor-Made Hairpin Conjugates of Achiral Seco-Cyclopropaneindoline-2-Benzofurancarboxamide and Pyrrole–Imidazole Polyamides. *Bioorg. Med. Chem. Lett.* **2005**, *15* (12), 3151–3156. https://doi.org/10.1016/j.bmcl.2005.04.006.

(129) Sato, A.; McNulty, L.; Cox, K.; Kim, S.; Scott, A.; Daniell, K.; Summerville, K.; Price, C.; Hudson, S.; Kiakos, K.; Hartley, J. A.; Asao, T.; Lee, M. A Novel Class of in Vivo Active Anticancer Agents: Achiral *Seco* -Amino- and *Seco* -Hydroxycyclopropylbenz[*e*]Indolone (*Seco* -CBI) Analogues of the Duocarmycins and CC-1065. *J. Med. Chem.* **2005**, *48* (11), 3903–3918. https://doi.org/10.1021/jm050179u.

(130) Shinohara, K.-I.; Sasaki, S.; Bando, T.; Sugiyama, H. Sequence-Specific Gene Silencing by Alkylating Py-Im Polyamide. *Nucl. Acid. Symp. Ser.* **2005**, *49*, 75–76.

(131) Toth, J.; Trzupek, J.; Flores, L.; Kiakos, K.; Hartley, J. A.; Pennington, W.; Lee, M. A Novel Achiral Seco-Amino-Cyclopropylindoline (CI) Analog of CC-1065 and the Duocarmycins: Design, Synthesis and Biological Studies. *Med. Chem.* **2005**, *1* (1), 13–19. https://doi.org/10.2174/1573406053402523.

(132) Bando, T.; Sasaki, S.; Minoshima, M.; Dohno, C.; Shinohara, K.; Narita, A.; Sugiyama, H. Efficient DNA Alkylation by a Pyrrole-Imidazole CBI Conjugate with an Indole Linker: Sequence-Specific Alkylation with Nine-Base-Pair Recognition. *Bioconjugate Chem.* **2006**, *17* (3), 715–720. https://doi.org/10.1021/bc060022w.

(133) Hartley, J.; Lee, M.; Kiakos, K.; Hudson, S.; Townes, H.; Summerville, K.; Scott, A.; Lingerfelt, B.; Purnell, B. Novel (S)-(-)- and R-(+)-Seco-Iso-Cyclopropylfurano[e]Indoline-5,6,7- Trimethoxyindole-2-Carboxamide (Iso-CFI) Analogs of Duocarmycin C2: Synthesis and Biological Evaluation. *Med. Chem.* **2006**, *2* (2), 139–146. https://doi.org/10.2174/157340606776056188.

(134) Minoshima, M.; Sasaki, S.; Shinohara, K.; Shimizu, T.; Bando, T.; Sugiyama, H. Molecular Design of DNA Alkylating Pyrrole-Imidazole Polyamides with Longer Recognition Sequence. *Nucl. Acid. Symp. Ser.* **2006**, *50* (1), 165–166. https://doi.org/10.1093/nass/nrl082.

(135) Purnell, B.; Sato, A.; O'Kelley, A.; Price, C.; Summerville, K.; Hudson, S.; O'Hare, C.; Kiakos, K.; Asao, T.; Lee, M.; Hartley, J. A. DNA Interstrand Crosslinking Agents: Synthesis, DNA Interactions, and Cytotoxicity of Dimeric Achiral Seco-Amino-CBI and Conjugates of Achiral Seco-Amino-CBI with Pyrrolobenzodiazepine (PBD). *Bioorg. Med. Chem. Lett.* **2006**, *16* (21), 5677–5681. https://doi.org/10.1016/j.bmcl.2006.08.005.

(136) Sasaki, S.; Bando, T.; Minoshima, M.; Shimizu, T.; Shinohara, K.; Takaoka, T.; Sugiyama, H. Sequence-Specific Alkylation of Double-Strand Human Telomere Repeat Sequence by Pyrrole-Imidazole Polyamides with Indole Linkers. *J. Am. Chem. Soc.* **2006**, *128* (37), 12162–12168. https://doi.org/10.1021/ja0626584.

(137) Sato, A.; Scott, A.; Asao, T.; Lee, M. Efficient Synthesis of Achiral *s Eco* -Cyclopropylbenz[2,3- *e*]Indoline Analogues: [4-Amino-2-(5,6,7-Trimethoxyindole-2-Carboxamido)Naphthalen-1-Yl]Ethyl Chloride and [4-Hydroxy-2-(5,6,7-Trimethoxyindole-2-Carboxamido)Naphthalen-1-Yl]Ethyl Chloride. *J. Org. Chem.* **2006**, *71* (12), 4692–4695. https://doi.org/10.1021/jo060501o.

(138) Shimizu, T.; Sasaki, S.; Minoshima, M.; Shinohara, K.; Bando, T.; Sugiyama, H. Synthesis and Evaluation of Sequence-Specific DNA Alkylating Agents: Effect of Alkylation Subunits. *Nucl. Acid. Symp. Ser.* **2006**, *50* (1), 155–156. https://doi.org/10.1093/nass/nrl077.

(139) Shinohara, K. -i. Alkylation of Template Strand of Coding Region Causes Effective Gene Silencing. *Nucl. Acid Res.* **2006**, *34* (4), 1189–1195. https://doi.org/10.1093/nar/gkl005.

(140) Shinohara, K.; Bando, T.; Sasaki, S.; Sakakibara, Y.; Minoshima, M.; Sugiyama, H. Antitumor Activity of Sequence-Specific Alkylating Agents: Pyrolle-Imidazole CBI Conjugates with Indole Linker. *Cancer Sci.* **2006**, *97* (3), 219–225. https://doi.org/10.1111/j.1349-7006.2006.00158.x.

(141) Tietze, L. F.; Major, F. Synthesis of New Water-Soluble DNA-Binding Subunits for Analogues of the Cytotoxic Antibiotic CC-1065 and Their Prodrugs. *Eur. J. Org. Chem.* **2006**, *2006* (10), 2314–2321. https://doi.org/10.1002/ejoc.200500060.

(142) Ganton, M. D.; Kerr, M. A. Aryl Amidation Routes to Dihydropyrrolo[3,2- *e*]Indoles and Pyrrolo[3,2- *f*]Tetrahydroquinolines: Total Synthesis of the (±)-CC-1065 CPI Subunit. *J. Org. Chem.* **2007**, *72* (2), 574–582. https://doi.org/10.1021/jo062064j.

(143) Kiakos, K.; Sato, A.; Asao, T.; McHugh, M. M.; Lee, M.; Hartley, J. A. DNA Sequence Selective Adenine Alkylation, Mechanism of Adduct Repair, and in Vivo Antitumor Activity of the Novel Achiral Seco-Amino-Cyclopropylbenz[e]Indolone Analogue of Duocarmycin AS-I-145. *Mol. Cancer Ther.* **2007**, *6* (10), 2708–2718. https://doi.org/10.1158/1535-7163.MCT-07-0294.

(144) Minoshima, M.; Sasaki, S.; Fujimoto, J.; Shinohara, K. -i.; Bando, T.; Sugiyama, H. Synthesis and Biological Properties of Pyrrole-Imidazole Polyamide Conjugates. *Nucl. Acid. Symp. Ser.* **2007**, *51* (1), 35–36. https://doi.org/10.1093/nass/nrm018.

(145) Minoshima, M.; Bando, T.; Sasaki, S.; Shinohara, K.; Shimizu, T.; Fujimoto, J.; Sugiyama, H. DNA Alkylation by Pyrrole−Imidazole *Seco* -CBI Conjugates with an Indole Linker: Sequence-Specific DNA Alkylation with 10-Base-Pair Recognition through Heterodimer Formation. *J. Am. Chem. Soc.* **2007**, *129* (17), 5384–5390. https://doi.org/10.1021/ja065235a.

(146) Sasaki, S.; Minoshima, M.; Fujimoto, J.; Shinohara, K. -i.; Bando, T.; Sugiyama, H. Sequence-Specific Alkylation by a Tandem Motif of Pyrrole-Imidazole CBI Conjugate. *Nucl. Acid. Symp. Ser.* **2007**, *51* (1), 265–266. https://doi.org/10.1093/nass/nrm133.

(147) Tichenor, M. S.; MacMillan, K. S.; Stover, J. S.; Wolkenberg, S. E.; Pavani, M. G.; Zanella, L.; Zaid, A. N.; Spalluto, G.; Rayl, T. J.; Hwang, I.; Baraldi, P. G.; Boger, D. L. Rational Design, Synthesis, and Evaluation of Key Analogues of CC-1065 and the Duocarmycins. *J. Am. Chem. Soc.* **2007**, *129* (45), 14092–14099. https://doi.org/10.1021/ja073989z.

(148) Tichenor, M. S.; MacMillan, K. S.; Trzupek, J. D.; Rayl, T. J.; Hwang, I.; Boger, D. L. Systematic Exploration of the Structural Features of Yatakemycin Impacting DNA Alkylation and Biological Activity. *J. Am. Chem. Soc.* **2007**, *129* (35), 10858–10869. https://doi.org/10.1021/ja072777z.

(149) Bando, T.; Minoshima, M.; Kashiwazaki, G.; Shinohara, K.; Sasaki, S.; Fujimoto, J.; Ohtsuki, A.; Murakami, M.; Nakazono, S.; Sugiyama, H. Requirement of β-Alanine Components in Sequence-Specific DNA Alkylation by Pyrrole–Imidazole Conjugates with Seven-Base Pair Recognition. *Bioorg. Med. Chem.* **2008**, *16* (5), 2286–2291. https://doi.org/10.1016/j.bmc.2007.11.064.

(150) Kashiwazaki, G.; Bando, T.; Sugiyama, H. Sequence-Specific Alkylation of DNA by Pyrrole-Imiclazole Polyamides through Cooperative Interaction. *Nucl. Acid. Symp. Ser.* **2008**, *52* (1), 365–366. https://doi.org/10.1093/nass/nrn184.

(151) Minoshima, M.; Chou, J.; Lefebvre, S.; Bando, T.; Shinohara, K. -i.; Gottesfeld, J. M.; Sugiyama, H. Targeting Specific Gene by Alkylating Pyrrole-Imidazole Polyamides. *Nucl. Acid. Symp. Ser.* **2008**, *52* (1), 363–364. https://doi.org/10.1093/nass/nrn183.

(152) Sasaki, S.; Bando, T.; Minoshima, M.; Shinohara, K.; Sugiyama, H. Sequence-Specific Alkylation by Y-Shaped and Tandem Hairpin Pyrrole–Imidazole Polyamides. *Chem. Eur. J.* **2008**, *14* (3), 864–870. https://doi.org/10.1002/chem.200700571.

(153) Tietze, L. F.; Schuster, H. J.; Hampel, S. M.; Rühl, S.; Pfoh, R. Enantio- and Diastereoselective Synthesis of Duocarmycine-Based Prodrugs for a Selective Treatment of Cancer by Epoxide Opening. *Chem. Eur. J.* **2008**, *14* (3), 895–901. https://doi.org/10.1002/chem.200700988.

(154) Gauss, C. M.; Hamasaki, A.; Parrish, J. P.; MacMillan, K. S.; Rayl, T. J.; Hwang, I.; Boger, D. L. Synthesis and Preliminary Evaluation of Duocarmycin Analogues Incorporating the 1,2,11,11a-Tetrahydrocyclopropa[c]Naphtho[2,3-e]Indol-4-One (CNI) and 1,2,11,11a-Tetrahydrocyclopropa[c]Naphtho[1,2-e]Indol-4-One (Iso-CNI) Alkylation Subunits. *Tetrahedron* **2009**, *65* (33), 6591–6599. https://doi.org/10.1016/j.tet.2009.02.065.

(155) MacMillan, K. S.; Lajiness, J. P.; Cara, C. L.; Romagnoli, R.; Robertson, W. M.; Hwang, I.; Baraldi, P. G.; Boger, D. L. Synthesis and Evaluation of a Thio Analogue of Duocarmycin SA. *Bioorg. Med. Chem. Lett.* **2009**, *19* (24), 6962–6965. https://doi.org/10.1016/j.bmcl.2009.10.063.

(156) MacMillan, K. S.; Nguyen, T.; Hwang, I.; Boger, D. L. Total Synthesis and Evaluation of *Iso* -Duocarmycin SA and *Iso* -Yatakemycin. *J. Am. Chem. Soc.* **2009**, *131* (3), 1187–1194. https://doi.org/10.1021/ja808108q.

(157) Minoshima, M.; Bando, T.; Shinohara, K. -i.; Sugiyama, H. Molecular Design of Sequence Specific DNA Alkylating Agents. *Nucl. Acid. Symp. Ser.* **2009**, *53* (1), 69–70. https://doi.org/10.1093/nass/nrp035.

(158) Neo, A. G.; Pérez, A.; López, C.; Castedo, L.; Tojo, G. Photocyclization of Tosylstilbenes as a Key Reaction in the Preparation of an Analogue of the Antitumor Agent CC-1065. *J. Org. Chem.* **2009**, *74* (8), 3203–3206. https://doi.org/10.1021/jo900140t.

(159) Boyle, K. E.; MacMillan, K. S.; Ellis, D. A.; Lajiness, J. P.; Robertson, W. M.; Boger, D. L. Synthesis and Evaluation of Duocarmycin SA Analogs Incorporating the Methyl 1,2,8,8a-Tetrahydrocyclopropa[c]Oxazolo[2,3-e]Indol-4-One-6-Carboxylate (COI) Alkylation Subunit. *Bioorg. Med. Chem. Lett.* **2010**, *20* (6), 1854–1857. https://doi.org/10.1016/j.bmcl.2010.01.145.

(160) Chavda, S.; Babu, B.; Yanow, S. K.; Jardim, A.; Spithill, T. W.; Kiakos, K.; Kluza, J.; Hartley, J. A.; Lee, M. A Novel Achiral Seco-Cyclopropylpyrido[e]Indolone (CPyI) Analog of CC-1065 and the Duocarmycins: Synthesis, DNA Interactions, in Vivo Anticancer and Anti-Parasitic Evaluation. *Bioorg. Med. Chem.* **2010**, *18* (14), 5016–5024. https://doi.org/10.1016/j.bmc.2010.05.078.

(161) Frecentese, F.; Fiorino, F.; Perissutti, E.; Severino, B.; Magli, E.; Esposito, A.; De Angelis, F.; Massarelli, P.; Nencini, C.; Viti, B.; Santagada, V.; Caliendo, G. Efficient Microwave Combinatorial Synthesis of Novel Indolic Arylpiperazine Derivatives as Serotoninergic Ligands. *Eur. J. Med. Chem.* **2010**, *45* (2), 752–759. https://doi.org/10.1016/j.ejmech.2009.11.023.

(162) Lajiness, J. P.; Boger, D. L. Synthesis and Characterization of a Cyclobutane Duocarmycin Derivative Incorporating the 1,2,10,11-Tetrahydro-9 *H* -Cyclobuta[*c*]Benzo[*e*]Indol-4-One (CbBI) Alkylation Subunit. *J. Am. Chem. Soc.* **2010**, *132* (39), 13936–13940. https://doi.org/10.1021/ja106986f.

(163) Minoshima, M.; Bando, T.; Shinohara, K.; Kashiwazaki, G.; Nishijima, S.; Sugiyama, H. Comparative Analysis of DNA Alkylation by Conjugates between Pyrrole–Imidazole Hairpin Polyamides and Chlorambucil or

Seco-CBI. *Bioorg. Med. Chem.* **2010**, *18* (3), 1236–1243. https://doi.org/10.1016/j.bmc.2009.12.033.

(164) Minoshima, M.; Chou, J. C.; Lefebvre, S.; Bando, T.; Shinohara, K.; Gottesfeld, J. M.; Sugiyama, H. Potent Activity against K562 Cells by Polyamide–Seco-CBI Conjugates Targeting Histone H4 Genes. *Bioorg. Med. Chem.* **2010**, *18* (1), 168–174. https://doi.org/10.1016/j.bmc.2009.11.005.

(165) Robertson, W. M.; Kastrinsky, D. B.; Hwang, I.; Boger, D. L. Synthesis and Evaluation of a Series of C5′-Substituted Duocarmycin SA Analogs. *Bioorg. Med. Chem. Lett.* **2010**, *20* (9), 2722–2725. https://doi.org/10.1016/j.bmcl.2010.03.078.

(166) Shinohara, K.-I.; Bando, T.; Sugiyama, H. Anticancer Activities of Alkylating Pyrrole–Imidazole Polyamides with Specific Sequence Recognition. *Anti-Cancer Drugs* **2010**, *21* (3), 228–242. https://doi.org/10.1097/CAD.0b013e328334d8f9.

(167) Sugiyama, H.; Bando, T. Indole Derivative for Alkylating Specific Base Sequence of DNA and Alkylating Agent and Drug Containing the Derivative. US007745473B2, June 29, 2010.

(168) Heinrich, D. M.; Youte, J.-J.; Denny, W. A.; Tercel, M. A New Enantioselective Approach to the Core Structure of Hypoxia Selective Prodrugs Related to the Duocarmycins. *Tet. Lett.* **2011**, *52* (51), 7000–7003. https://doi.org/10.1016/j.tetlet.2011.10.105.

(169) Lajiness, J. P.; Boger, D. L. Asymmetric Synthesis of 1,2,9,9a-Tetrahydrocyclopropa[*c*]Benzo[*e*]Indol-4-One (CBI). *J. Org. Chem.* **2011**, *76* (2), 583–587. https://doi.org/10.1021/jo102136w.

(170) Kashiwazaki, G.; Bando, T.; Yoshidome, T.; Masui, S.; Takagaki, T.; Hashiya, K.; Pandian, G. N.; Yasuoka, J.; Akiyoshi, K.; Sugiyama, H. Synthesis and Biological Properties of Highly Sequence-Specific-Alkylating *N* -Methylpyrrole– *N* -Methylimidazole Polyamide Conjugates. *J. Med. Chem.* **2012**, *55* (5), 2057–2066. https://doi.org/10.1021/jm201225z.

(171) Rayburn, E.; Wang, W.; Li, M.; Zhang, X.; Xu, H.; Li, H.; Qin, J.-J.; Jia, L.; Covey, J.; Lee, M.; Zhang, R. Preclinical Pharmacology of Novel Indolecarboxamide ML-970, an Investigative Anticancer Agent. *Cancer Chemother. Pharmacol.* **2012**, *69* (6), 1423–1431. https://doi.org/10.1007/s00280-012-1851-9.

(172) Takagaki, T.; Bando, T.; Sugiyama, H. Synthesis of Pyrrole−Imidazole Polyamide Seco-1-Chloromethyl-5-Hydroxy-1,2-Dihydro-3H-benz[e]Indole Conjugates with a Vinyl Linker Recognizing a 7 Bp DNA Sequence. *J. Am. Chem. Soc.* **2012**, *134*, 13074–13081. https://doi.org/10.1021/ja3044294.

(173) Neo, A. G.; López, C.; López, A.; Castedo, L.; Tojo, G. Studies on the Synthesis of a Hindered Analogue of the Antitumour Agent CC-1065. *Tetrahedron* **2013**, *69* (51), 11010–11016. https://doi.org/10.1016/j.tet.2013.10.012.

(174) Patil, P.; Cousins, K.; Smith, M.; Wieskamp, S.; Ferrara, M.; Bruce, C. D.; Lee, M. Controlling the Radical 5-Exo-Trig Cyclization, and Selective Synthesis of Seco-Iso-Cyclopropylfurano[e]Indoline (Seco-Iso-CFI) and Seco-Cyclopropylthiophene[e]Indoline (Seco-CTI) DNA Alkylating Subunit of the Duocarmycins. *Tet. Lett.* **2013**, *54* (35), 4756–4759. https://doi.org/10.1016/j.tetlet.2013.06.116.

(175) Wolfe, A. L.; Duncan, K. K.; Lajiness, J. P.; Zhu, K.; Duerfeldt, A. S.; Boger, D. L. A Fundamental Relationship between Hydrophobic Properties and Biological Activity for the Duocarmycin Class of DNA-Alkylating Antitumor Drugs: Hydrophobic-Binding-Driven Bonding. *J. Med. Chem.* **2013**, *56* (17), 6845–6857. https://doi.org/10.1021/jm400665c.

(176) Patil, P. C.; Lee, M. An Efficient Synthesis of Furano Analogs of Duocarmycin C1 and C2: Seco-Iso-Cyclopropylfurano[e]Indoline-Trimethoxyindole and Seco-Cyclopropylfurano[f]Quinoline-Trimethoxyindole. *Tet. Lett.* **2014**, *55* (21), 3283–3285. https://doi.org/10.1016/j.tetlet.2014.04.062.

(177) Taylor, R. D.; Asamitsu, S.; Takenaka, T.; Yamamoto, M.; Hashiya, K.; Kawamoto, Y.; Bando, T.; Nagase, H.; Sugiyama, H. Sequence-Specific DNA Alkylation Targeting for Kras Codon 13 Mutation by Pyrrole-Imidazole Polyamide *Seco* -CBI Conjugates. *Chem. Eur. J.* **2014**, *20* (5), 1310–1317. https://doi.org/10.1002/chem.201303295.

(178) Taylor, R. D.; Kawamoto, Y.; Hashiya, K.; Bando, T.; Sugiyama, H. Sequence-Specific DNA Alkylation by Tandem Py-Im Polyamide Conjugates. *Chem. Asian J.* **2014**, *9* (9), 2527–2533. https://doi.org/10.1002/asia.201402331.

(179) Tercel, M.; Pruijn, F. B.; O'Connor, P. D.; Liyanage, H. D. S.; Atwell, G. J.; Alix, S. M. Mechanism of Action of AminoCBIs: Highly Reactive but Highly Cytotoxic Analogues of the Duocarmycins. *ChemBioChem* **2014**, *15* (13), 1998–2006. https://doi.org/10.1002/cbic.201402256.

(180) Yamamoto, M.; Bando, T.; Kawamoto, Y.; Taylor, R. D.; Hashiya, K.; Sugiyama, H. Specific Alkylation of Human Telomere Repeat Sequences by a Tandem-Hairpin Motif of Pyrrole–Imidazole Polyamides with Indole- *Seco* -CBI. *Bioconjugate Chem.* **2014**, *25* (3), 552–559. https://doi.org/10.1021/bc400567m.

(181) Hiraoka, K.; Inoue, T.; Taylor, R. D.; Watanabe, T.; Koshikawa, N.; Yoda, H.; Shinohara, K.; Takatori, A.; Sugimoto, H.; Maru, Y.; Denda, T.; Fujiwara, K.; Balmain, A.; Ozaki, T.; Bando, T.; Sugiyama, H.; Nagase, H. Inhibition of KRAS Codon 12 Mutants Using a Novel DNA-Alkylating Pyrrole–Imidazole Polyamide Conjugate. *Nat. Comm.* **2015**, *6* (1), 6706. https://doi.org/10.1038/ncomms7706.

(182) Stephenson, M. J.; Howell, L. A.; O'Connell, M. A.; Fox, K. R.; Adcock, C.; Kingston, J.; Sheldrake, H.; Pors, K.; Collingwood, S. P.; Searcey, M. Solid-Phase Synthesis of Duocarmycin Analogues and the Effect of C-Terminal Substitution on Biological Activity. *J. Org. Chem.* **2015**, *80* (19), 9454–9467. https://doi.org/10.1021/acs.joc.5b01373.

(183) Taylor, R. D.; Chandran, A.; Kashiwazaki, G.; Hashiya, K.; Bando, T.; Nagase, H.; Sugiyama, H. Selective Targeting of the KRAS Codon 12 Mutation Sequence by Pyrrole-Imidazole Polyamide *Seco* -CBI Conjugates. *Chem. Eur. J.* **2015**, *21* (42), 14996–15003. https://doi.org/10.1002/chem.201501870.

(184) Twum, E. A.; Nathubhai, A.; Wood, P. J.; Lloyd, M. D.; Thompson, A. S.; Threadgill, M. D. Initial Development of a Cytotoxic Amino-Seco-CBI Warhead for Delivery by Prodrug Systems. *Bioorg. Med. Chem.* **2015**, *23* (13), 3481–3489. https://doi.org/10.1016/j.bmc.2015.04.034.

(185) Chanda, P. B.; Boyle, K. E.; Brody, D. M.; Shukla, V.; Boger, D. L. Synthesis and Evaluation of Duocarmycin SA Analogs Incorporating the Methyl 1,2,8,8a-Tetrahydrocyclopropa[c]Imidazolo[4,5-e]Indol-4-One-6-Carboxylate (CImI) Alkylation Subunit. *Bioorg. Med. Chem.* **2016**, *24* (20), 4779–4786. https://doi.org/10.1016/j.bmc.2016.04.050.

(186) Guo, C.; Asamitsu, S.; Kashiwazaki, G.; Sato, S.; Bando, T.; Sugiyama, H. DNA Interstrand Crosslinks by H-

Pin Polyamide (*S*) -*Seco* -CBI Conjugates. *ChemBioChem* **2017**, *18* (2), 166–170. https://doi.org/10.1002/cbic.201600425.

(187) Stephenson, M. J.; Howell, L. A.; Searcey, M. Synthesis of Duocarmycin Analogues. US009765077B2, September 19, 2017.

(188) Kiakos, K.; Englinger, B.; Yanow, S. K.; Wernitznig, D.; Jakupec, M. A.; Berger, W.; Keppler, B. K.; Hartley, J. A.; Lee, M.; Patil, P. C. Design, Synthesis, Nuclear Localization, and Biological Activity of a Fluorescent Duocarmycin Analog, HxTfA. *Bioorg. Med. Chem. Lett.* **2018**, *28* (8), 1342–1347. https://doi.org/10.1016/j.bmcl.2018.03.016.

(189) Wang, S.; Chen, B.; Dragovich, P.; Pillow, T.; Staben, L.; Guo, J.; Su, D.; Zhang, C.; Bobba, S.; Ma, Y.; Wang, J.; Sangaraju, D.; Wei, B.; Phillips, G. L.; Khojasteh, C.; Zhang, D. A Novel Depurination Methodology to Assess DNA Alkylation of Chloro-Bis-Seco-Cyclopropylbenzoindoles Allowed for Comparison of Minor-Groove Reactivity. *Drug Metab Dispos* **2019**, *47* (5), 547–555. https://doi.org/10.1124/dmd.118.085209.

(190) Maeda, R.; Ito, S.; Hashiya, K.; Bando, T.; Sugiyama, H. DNA Alkylation of the RUNX-Binding Sequence by CBI–PI Polyamide Conjugates**. *Chem. Eur. J.* **2020**, *26* (64), 14639–14644. https://doi.org/10.1002/chem.202002166.

(191) Maeda, R.; Bando, T.; Sugiyama, H. Application of DNA-Alkylating Pyrrole-Imidazole Polyamides for Cancer Treatment. *ChemBioChem* **2021**, *22* (9), 1538–1545. https://doi.org/10.1002/cbic.202000752

(2) prodrugs/bifunctionals (102 items - 84 journal publications, 18 patents)

(1) Nagamura, S.; Asai, A.; Kanda, Y.; Kobayashi, E.; Gomi, K.; Saito, H. Synthesis and Antitumor Activity of Duocarmycin Derivatives: Modification of Segment A of Duocarmycin B2. *Chem. Pharm. Bull.* **1996**, *44* (9), 1723–1730. https://doi.org/10.1248/cpb.44.1723.

(2) Nagamura, S.; Kobayashi, E.; Gomi, K.; Saito, H. Synthesis and Antitumor Activity of Duocarmycin Derivatives: A-Ring Pyrrole Analogues of Duocarmycin B2. *Bioorg. Med. Chem.* **1996**, *4* (8), 1379–1391. https://doi.org/10.1016/0968-0896(96)00132-0.

(3) Boger, D. L.; Han, N. CC-1065/Duocarmycin and Bleomycin A2 Hybrid Agents: Lack of Enhancement of DNA Alkylation by Attachment to Noncomplementary DNA Binding Subunits. *Bioorg. Med. Chem.* **1997**, *5* (2), 233–243. https://doi.org/10.1016/S0968-0896(96)00237-4.

(4) Nagamura, S.; Asai, A.; Amishiro, N.; Kobayashi, E.; Gomi, K.; Saito, H. Synthesis and Antitumor Activity of Duocarmycin Derivatives: A-Ring Pyrrole Compounds Bearing Cinnamoyl Groups. *J. Med. Chem.* **1997**, *40* (6), 972–979. https://doi.org/10.1021/jm9606094.

(5) Nagamura, S.; Asai, A.; Kobayashi, E.; Gomi, K.; Saito, H. Studies on Duocarmycin SA and Its Derivatives. *Bioorg. Med. Chem.* **1997**, *5* (3), 623–630. https://doi.org/10.1016/S0968-0896(96)00276-3.

(6) Powell, M. J. Cyclopropylindole Prodrugs. US005646298A, July 8, 1997.

(7) Denny, W. A.; Tercel, M.; Atwell, G. J.; Milbank, J. B. J. Precursors of Cyclopropylindols and Their Use as Prodrugs. AU199854192B2, March 9, 1998.

(8) Larrick, J. W.; Wang, Y.; Wright, S. C. DNA-Binding Indole Derivatives, Their Prodrugs and Immunoconjugates as Anticancer Agents. US995843937A, December 1, 1998.

(9) Amishiro, N.; Nagamura, S.; Kobayashi, E.; Gomi, K.; Saito, H. New Water-Soluble Duocarmycin Derivatives: Synthesis and Antitumor Activity of A-Ring Pyrrole Compounds Bearing β-Heteroarylacryloyl Groups. *J. Med. Chem.* **1999**, *42* (4), 669–676. https://doi.org/10.1021/jm980559y.

(10) Asai, A.; Nagamura, S.; Kobayashi, E.; Gomi, K.; Saito, H. Synthesis and Antitumor Activity of Water-Soluble Duocarmycin B1 Prodrugs. *Bioorg. Med. Chem. Lett.* **1999**, *9* (20), 2995–2998. https://doi.org/10.1016/S0960-894X(99)00518-1.

(11) Denny, W. A.; Tercel, M. Cyclopropylindoles and Their Seco Precursors, and Their Use as Prodrugs. US005985909A, November 16, 1999.

(12) Denny, W. A.; Tercel, M.; Atwell, G. J. Cyclopropylindole Compounds and Their Use as Prodrugs. EP0938474B1, September 1, 1999.

(13) Hay, M. P.; Sykes, B. M.; Denny, W. A.; Wilson, W. R. A 2-Nitroimiazole Carbamate Prodrug of 5-Amino-1-(Chloromethyl)-3-[(5,6,7-Trimethoxyindol-2-Yl)Carbonyl]-1,2-Dihydro-3H-Benz[e]Indole (Amino-Seco-CBI-TMI) for Use with ADEPT and GDEPT. *Bioorg. Med. Chem. Lett.* **1999**, *9*, 2237–2242. https://doi.org/10.1016/s0960-894x(99)00381-9.

(14) Tercel, M.; Gieseg, M. A.; Denny, W. A.; Wilson, W. R. Synthesis and Cytotoxicity of Amino-Seco-DSA: An Amino Analogue of the DNA Alkylating Agent Duocarmycin SA. *J. Org. Chem.* **1999**, *64*, 5946–5953. https://doi.org/10.1021/jo990464j.

(15) Denny, W. A.; Tercel, M.; Atwell, G. J. Condensed N-Acylindoles as Antitumor Agents. US006130237A, Oc 2000.

(16) Denny, W. A.; Tercel, M.; Atwell, G. J.; Milbank, J. J. B. Seco Precursors of Cyclopropylindolines and Their Use as Prodrugs. US006251933B1, June 26, 2001.

(17) Tietze, L. F.; Herzig, T.; Fecher, A.; Haunert, F.; Schuberth, I. Highly Selective Glycosylated Prodrugs of Cytostatic CC-1065 Analogues for Antibody-Directed Enzyme Tumor Therapy. *ChemBioChem* **2001**, *2* (10), 758. https://doi.org/10.1002/1439-7633(20011001)2:10<758::AID-CBIC758>3.0.CO;2-G.

(18) Tietze, L. F.; Lieb, M.; Herzig, T.; Haunert, F.; Schuberth, I. A Strategy for Tumor-Selective Chemotherapy by Enzymatic Liberation of Seco-Duocarmycin SA-Derivatives from Nontoxic Prodrugs. *Bioorg. Med. Chem.* **2001**, *9* (7), 1929–1939. https://doi.org/10.1016/S0968-0896(01)00098-0.

(19) Wang, Y.; Yuan, H.; Wright, S. C.; Wang, H.; Larrick, J. W. Synthesis and Preliminary Cytotoxicity Study of a Cephalosporin-CC-1065 Analogue Prodrug. *BMC Chem. Bio.* **2001**, *1*, 4–8.

(20) Zhou, Q.; Duan, W.; Simmons, D.; Shayo, Y.; Raymond, M. A.; Dorr, R. T.; Hurley, L. H. Design and Synthesis of a Novel DNA−DNA Interstrand Adenine−Guanine Cross-Linking Agent. *J. Am. Chem. Soc.*

2001, *123* (20), 4865–4866. https://doi.org/10.1021/ja005658r.

(21) Tietze, L. F.; Feuerstein, T.; Fecher, A.; Haunert, F.; Panknin, O.; Borchers, U.; Schuberth, I.; Alves, F. Proof of Principle in the Selective Treatment of Cancer by Antibody-Directed Enzyme Prodrug Therapy: The Development of a Highly Potent Prodrug. *Angew. Chem. Int. Ed.* **2002**, *41* (5), 759. https://doi.org/10.1002/1521-3773(20020301)41:5<759::AID-ANIE759>3.0.CO;2-7.

(22) Tietze, L. F.; Herzig, T.; Feuerstein, T.; Schuberth, I. Synthesis and Biological Evaluation of Novel Analogues and Prodrugs of the Cytotoxic Antibiotic CC-1065 for Selective Cancer Therapy. *Eur. J. Org. Chem.* **2002**, *2002* (10), 1634–1645. https://doi.org/10.1002/1099-0690(200205)2002:10<1634::AID-EJOC1634>3.0.CO;2-Y.

(23) Townes, H.; Summerville, K.; Purnell, B. L.; Hooker, M.; Madsen, E. C.; Hudson, S.; Lee, M. Investigation of a Novel Reductively-Activatable Anticancer Prodrug of Seco -CBI-TMI, an Analog of Duocarmycin SA. *Med. Chem. Res.* **2002**.

(24) Wang, Y.-D.; Dziegielewski, J.; Chang, A. Y.; Dervan, P. B.; Beerman, T. A. Cell-Free and Cellular Activities of a DNA Sequence Selective Hairpin Polyamide-CBI Conjugate. *Journal of Biological Chemistry* **2002**, *277* (45), 42431–42437. https://doi.org/10.1074/jbc.M207179200.

(30) Yongxin, R.; Chari, R. V. J. CC-1065 Analogs Synthesis. US006534660B1, March 18, 2003.

(31) Zhao, R. Y.; Chari, R. V. J. Prodrugs of CC-1065 Analogs. US007655660B2, June 29, 2004.

(32) Ahn, G.-O.; Botting, K. J.; Patterson, A. V.; Ware, D. C.; Tercel, M.; Wilson, W. R. Radiolytic and Cellular Reduction of a Novel Hypoxia-Activated Cobalt(III) Prodrug of a Chloromethylbenzindoline DNA Minor Groove Alkylator. *Biochem. Pharm.* **2006**, *71* (12), 1683–1694. https://doi.org/10.1016/j.bcp.2006.03.007.

(33) Tietze, L. F.; Krewer, B.; Frauendorf, H.; Major, F.; Schuberth, I. Investigation of Reactivity and Selectivity of DNA-Alkylating Duocarmycin Analogues by High-Resolution Mass Spectrometry. *Angew. Chem. Int. Ed.* **2006**, *45* (39), 6570–6574. https://doi.org/10.1002/anie.200600935.

(34) Tietze, L. F.; Major, F.; Schuberth, I. Antitumor Agents: Development of Highly Potent Glycosidic Duocarmycin Analogues for Selective Cancer Therapy. *Angew. Chem. Int. Ed.* **2006**, *45* (39), 6574–6577. https://doi.org/10.1002/anie.200600936.

(35) Wang, Y.; Li, L.; Tian, Z.; Jiang, W.; Larrick, J. W. Synthesis and Antitumor Activity of CBI-Bearing Ester and Carbamate Prodrugs of CC-1065 Analogue. *Bioorg. Med. Chem.* **2006**, *14* (23), 7854–7861. https://doi.org/10.1016/j.bmc.2006.07.062.

(36) Jin, W.; Trzupek, J. D.; Rayl, T. J.; Broward, M. A.; Vielhauer, G. A.; Weir, S. J.; Hwang, I.; Boger, D. L. A Unique Class of Duocarmycin and CC-1065 Analogues Subject to Reductive Activation. *J. Am. Chem. Soc.* **2007**, *129* (49), 15391–15397. https://doi.org/10.1021/ja075398e.

(37) Tietze, L. F.; Major, F.; Schuberth, I.; Spiegl, D. A.; Krewer, B.; Maksimenka, K.; Bringmann, G.; Magull, J. Selective Treatment of Cancer: Synthesis, Biological Evaluation and Structural Elucidation of Novel Analogues of the Antibiotic CC-1065 and the Duocarmycins. *Chem.*

(25) Wang, Y.; Yuan, H.; Wright, S. C.; Wang, H.; Larrick, J. W. Synthesis and Cytotoxicity of a Biotinylated CC-1065 Analogue. *BMC Chem. Bio.* **2002**, *2*, 1–4. https://doi.org/10.1186/1472-6769-2-1.

(26) Hay, M. P.; Anderson, R. F.; Ferry, D. M.; Wilson, W. R.; Denny, W. A. Synthesis and Evaluation of Nitroheterocyclic Carbamate Prodrugs for Use with Nitroreductase-Mediated Gene-Directed Enzyme Prodrug Therapy. *J. Med. Chem.* **2003**, *46* (25), 5533–5545. https://doi.org/10.1021/jm030308b.

(27) Hay, M. P.; Atwell, G. J.; Wilson, W. R.; Pullen, S. M.; Denny, W. A. Structure−Activity Relationships for 4-Nitrobenzyl Carbamates of 5-Aminobenz[*e*]Indoline Minor Groove Alkylating Agents as Prodrugs for GDEPT in Conjunction with *E. c Oli* Nitroreductase. *J. Med. Chem.* **2003**, *46* (12), 2456–2466. https://doi.org/10.1021/jm0205191.

(28) Tercel, M.; Stribbling, S. M.; Sheppard, H.; Siim, B. G.; Wu, K.; Pullen, S. M.; Botting, K. J.; Wilson, W. R.; Denny, W. A. Unsymmetrical DNA Cross-Linking Agents: Combination of the CBI and PBD Pharmacophores. *J. Med. Chem.* **2003**, *46* (11), 2132–2151. https://doi.org/10.1021/jm020526p.

(29) Wang, Y.; Yuan, H.; Wright, S. C.; Wang, H.; Larrick, J. W. Synthesis and Preliminary Cytotoxicity Study of Glucuronide Derivatives of CC-1065 Analogues. *Bioorg. Med. Chem.* **2003**, *11* (7), 1569–1575. https://doi.org/10.1016/S0968-0896(02)00603-X.

Eur. J. **2007**, *13* (16), 4396–4409. https://doi.org/10.1002/chem.200700113.

(38) Gangwar, S.; Thang, Q. Methods and Compounds for Preparing CC-1065 Analogs. US 20080281102A1, November 13, 2008.

(39) Tietze, L. F.; Panknin, O.; Major, F.; Krewer, B. Synthesis of a Novel Pentagastrin-Drug Conjugate for a Targeted Tumor Therapy. *Chem. Eur. J.* **2008**, *14* (9), 2811–2818. https://doi.org/10.1002/chem.200701521.

(40) Tietze, L. F.; Schuster, H. J.; Schmuck, K.; Schuberth, I.; Alves, F. Duocarmycin-Based Prodrugs for Cancer Prodrug Monotherapy. *Bioorg. Med. Chem.* **2008**, *16* (12), 6312–6318. https://doi.org/10.1016/j.bmc.2008.05.009.

(41) Tietze, L. F.; von Hof, J. M.; Krewer, B.; Müller, M.; Major, F.; Schuster, H. J.; Schuberth, I.; Alves, F. Asymmetric Synthesis and Biological Evaluation of Glycosidic Prodrugs for a Selective Cancer Therapy. *ChemMedChem* **2008**, *3* (12), 1946–1955. https://doi.org/10.1002/cmdc.200800250.

(42) Tietze, L.; Panknin, O.; Krewer, B.; Major, F.; Schuberth, I. Synthesis and Biological Evaluation of a Novel Pentagastrin- Toxin Conjugate Designed for a Targeted Prodrug Monotherapy of Cancer. *Int. J. Mol. Sci.* **2008**, *9* (5), 821–837. https://doi.org/10.3390/ijms9050821.

(43) Wang, Y.; Jiang, J.; Jiang, X.; Cai, S.; Han, H.; Li, L.; Tian, Z.; Jiang, W.; Zhang, Z.; Xiao, Y.; Wright, S. C.; Larrick, J. W. Synthesis and Antitumor Activity Evaluations of Albumin-Binding Prodrugs of CC-1065 Analog. *Bioorg. Med. Chem.* **2008**, *16* (13), 6552–6559. https://doi.org/10.1016/j.bmc.2008.05.025.

(44) Milbank, J. B. J.; Stevenson, R. J.; Ware, D. C.; Chang, J. Y. C.; Tercel, M.; Ahn, G.-O.; Wilson, W. R.; Denny, W. A. Synthesis and Evaluation of Stable Bidentate Transition Metal Complexes of 1-(Chloromethyl)-5-Hydroxy-3-(5,6,7-Trimethoxyindol-2-Ylcarbonyl)-2,3-Dihydro-1 *H* -Pyrrolo[3,2- *f*]Quinoline (*Seco* -6-AzaCBI-TMI) as Hypoxia

Selective Cytotoxins. *J. Med. Chem.* **2009**, *52* (21), 6822–6834. https://doi.org/10.1021/jm9008746.

(45) Tercel, M.; Atwell, G. J.; Yang, S.; Stevenson, R. J.; Botting, K. J.; Boyd, M.; Smith, E.; Anderson, R. F.; Denny, W. A.; Wilson, W. R.; Pruijn, F. B. Hypoxia-Activated Prodrugs: Substituent Effects on the Properties of Nitro *Seco* -1,2,9,9a-Tetrahydrocyclopropa[*c*]Benz[*e*]Indol-4-One (NitroCBI) Prodrugs of DNA Minor Groove Alkylating Agents. *J. Med. Chem.* **2009**, *52* (22), 7258–7272. https://doi.org/10.1021/jm901202b.

(46) Tietze, L. F.; Krewer, B. Novel Analogues of CC-1065 and the Duocarmycins for the Use in Targeted Tumour Therapies. *Anti-Cancer Agents in Med. Chem.* **2009**, *9* (3), 304–325. https://doi.org/10.2174/1871520610909030304.

(47) Tietze, L. F.; Schuster, H. J.; Krewer, B.; Schuberth, I. Synthesis and Biological Studies of Different Duocarmycin Based Glycosidic Prodrugs for Their Use in the Antibody-Directed Enzyme Prodrug Therapy. *J. Med. Chem.* **2009**, *52* (2), 537–543. https://doi.org/10.1021/jm8009102.

(48) Tietze, L.; Krewer, B.; Von Hof, J. M.; Frauendorf, H.; Schuberth, I. Determination of the Biological Activity and Structure Activity Relationships of Drugs Based on the Highly Cytotoxic Duocarmycins and CC-1065. *Toxins* **2009**, *1* (2), 134–150. https://doi.org/10.3390/toxins1020134.

(49) Wilson, W. R.; Stribbling, S. M.; Pruijn, F. B.; Syddall, S. P.; Patterson, A. V.; Liyanage, H. D. S.; Smith, E.; Botting, K. J.; Tercel, M. Nitro-Chloromethylbenzindolines: Hypoxia-Activated Prodrugs of Potent Adenine *N* 3 DNA Minor Groove Alkylators. *Mol. Cancer Ther.* **2009**, *8* (10), 2903–2913. https://doi.org/10.1158/1535-7163.MCT-09-0571.

(50) Denny, W. A.; Wilson, W. R.; Stevenson, R. J.; Tercel, M.; Atwell, G. J.; Yang, S.; Patterson, A. V.; Pruijn, F. B. Nitrobenzindoles and Their Use in Cancer Therapy. US007718688B2, May 18, 2010.

(51) Lajiness, J. P.; Robertson, W. M.; Dunwiddie, I.; Broward, M. A.; Vielhauer, G. A.; Weir, S. J.; Boger, D. L. Design, Synthesis, and Evaluation of Duocarmycin *O* -Amino Phenol Prodrugs Subject to Tunable Reductive Activation. *J. Med. Chem.* **2010**, *53* (21), 7731–7738. https://doi.org/10.1021/jm1010397.

(52) Schuster, H. J.; Krewer, B.; von Hof, J. M.; Schmuck, K.; Schuberth, I.; Alves, F.; Tietze, L. F. Synthesis of the First Spacer Containing Prodrug of a Duocarmycin Analogue and Determination of Its Biological Activity. *Org. Biomol. Chem.* **2010**, *8* (8), 1833. https://doi.org/10.1039/b925070k.

(53) Tercel, M.; Yang, S.; Atwell, G. J.; Smith, E.; Gu, Y.; Anderson, R. F.; Denny, W. A.; Wilson, W. R.; Pruijn, F. B. Hypoxic Selectivity and Solubility—Investigating the Properties of A-Ring Substituted Nitro Seco-1,2,9,9a-Tetrahydrocyclopropa[c]Benz[e]Indol-4-Ones (NitroCBIs) as Hypoxia-Activated Prodrugs for Antitumor Therapy. *Bioorg. Med. Chem.* **2010**, *18* (14), 4997–5006. https://doi.org/10.1016/j.bmc.2010.06.001.

(54) Tietze, L. F.; Behrendt, F.; Major, F.; Krewer, B.; von Hof, J. M. Synthesis of Fluorescence-Labelled Glycosidic Prodrugs Based on the Cytotoxic Antibiotic Duocarmycin. *Eur. J. Org. Chem.* **2010**, *2010* (36), 6909–6921. https://doi.org/10.1002/ejoc.201000966.

(55) Tietze, L. F.; von Hof, J. M.; Müller, M.; Krewer, B.; Schuberth, I. Glycosidic Prodrugs of Highly Potent Bifunctional Duocarmycin Derivatives for Selective Treatment of Cancer. *Angew. Chem. Int. Ed.* **2010**, *49* (40), 7336–7339. https://doi.org/10.1002/anie.201002502.

(56) Ashoorzadeh, A.; Atwell, G. J.; Pruijn, F. B.; Wilson, W. R.; Tercel, M.; Denny, W. A.; Stevenson, R. J. The Effect of Sulfonate Leaving Groups on the Hypoxia-Selective Toxicity of Nitro Analogs of the Duocarmycins. *Bioorg. Med. Chem.* **2011**, *19* (16), 4851–4860. https://doi.org/10.1016/j.bmc.2011.06.073.

(57) Boger, D. L. CBI Derivatives Subject to Reductive Activation. US 20110112163A1, May 12, 2011.

(58) Lu, G.-L.; Stevenson, R. J.; Chang, J. Y.-C.; Brothers, P. J.; Ware, D. C.; Wilson, W. R.; Denny, W. A.; Tercel, M. N-Alkylated Cyclen Cobalt(III) Complexes of 1-(Chloromethyl)-3-(5,6,7-Trimethoxyindol-2-Ylcarbonyl)-2,3-Dihydro-1H-Pyrrolo[3,2-f]Quinolin-5-Ol DNA Alkylating Agent as Hypoxia-Activated Prodrugs. *Bioorg. Med. Chem.* **2011**, *19* (16), 4861–4867. https://doi.org/10.1016/j.bmc.2011.06.076.

(59) Park, S.; Bando, T.; Shinohara, K.; Nishijima, S.; Sugiyama, H. Photocontrollable Sequence-Specific DNA Alkylation by a Pyrrole−Imidazole Polyamide *Seco* -CBI Conjugate. *Bioconjugate Chem.* **2011**, *22* (2), 120–124. https://doi.org/10.1021/bc100352y.

(60) Pors, K.; Loadman, P. M.; Shnyder, S. D.; Sutherland, M.; Sheldrake, H. M.; Guino, M.; Kiakos, K.; Hartley, J. A.; Searcey, M.; Patterson, L. H. Modification of the Duocarmycin Pharmacophore Enables CYP1A1 Targeting for Biological Activity. *Chem. Commun.* **2011**, *47* (44), 12062. https://doi.org/10.1039/c1cc15638a.

(61) Seubert, C. M.; Stritzker, J.; Hess, M.; Donat, U.; Sturm, J. B.; Chen, N.; Von Hof, J. M.; Krewer, B.; Tietze, L. F.; Gentschev, I.; Szalay, A. A. Enhanced Tumor Therapy Using Vaccinia Virus Strain GLV-1h68 in Combination with a β-Galactosidase-Activatable Prodrug Seco-Analog of Duocarmycin SA. *Cancer Gene Ther.* **2011**, *18* (1), 42–52. https://doi.org/10.1038/cgt.2010.49.

(62) Stevenson, R. J.; Denny, W. A.; Ashoorzadeh, A.; Pruijn, F. B.; van Leeuwen, W. F.; Tercel, M. The Effect of a Bromide Leaving Group on the Properties of Nitro Analogs of the Duocarmycins as Hypoxia-Activated Prodrugs and Phosphate Pre-Prodrugs for Antitumor Therapy. *Bioorg. Med. Chem.* **2011**, *19* (20), 5989–5998. https://doi.org/10.1016/j.bmc.2011.08.045.

(63) Takagaki, T.; Bando, T.; Kitano, M.; Hashiya, K.; Kashiwazaki, G.; Sugiyama, H. Evaluation of PI Polyamide Conjugates with Eight-Base Pair Recognition and Improvement of the Aqueous Solubility by PEGylation. *Bioorg. Med. Chem.* **2011**, *19*, 5896–5902. https://doi.org/10.1016/j.bmc.2011.08.009.

(64) Tercel, M.; Atwell, G. J.; Yang, S.; Ashoorzadeh, A.; Stevenson, R. J.; Botting, K. J.; Gu, Y.; Mehta, S. Y.; Denny, W. A.; Wilson, W. R.; Pruijn, F. B. Selective Treatment of Hypoxic Tumor Cells In Vivo: Phosphate Pre-Prodrugs of Nitro Analogues of the Duocarmycins. *Angew. Chem. Int. Ed.* **2011**, *50* (11), 2606–2609. https://doi.org/10.1002/anie.201004456.

(65) Tercel, M.; Lee, H. H.; Yang, S.; Liyanage, H. D. S.; Mehta, S. Y.; Boyd, P. D. W.; Jaiswal, J. K.; Tan, K. L.; Pruijn, F. B. Preparation and Antitumour Properties of the Enantiomers of a Hypoxia-Selective Nitro Analogue of the Duocarmycins. *ChemMedChem* **2011**, *6* (10), 1860–1871. https://doi.org/10.1002/cmdc.201100271.

(66) Tietze, L. F.; Schmuck, K.; Schuster, H. J.; Müller, M.; Schuberth, I. Synthesis and Biological Evaluation of Prodrugs Based on the Natural Antibiotic Duocarmycin for Use in ADEPT and PMT. *Chem. Eur. J.* **2011**, *17* (6), 1922–1929. https://doi.org/10.1002/chem.201002798.

(67) Stevenson, R. J.; Denny, W. A.; Tercel, M.; Pruijn, F. B.; Ashoorzadeh, A. Nitro *Seco* Analogues of the Duocarmycins Containing Sulfonate Leaving Groups as Hypoxia-Activated Prodrugs for Cancer Therapy. *J. Med. Chem.* **2012**, *55* (6), 2780–2802. https://doi.org/10.1021/jm201717y.

(68) Tietze, L. F.; Behrendt, F.; Pestel, G. F.; Schuberth, I.; Mitkovski, M. Synthesis, Biological Evaluation, and Live Cell Imaging of Novel Fluorescent Duocarmycin Analogs. *Chem. & Biodiv.* **2012**, *9* (11), 2559–2570. https://doi.org/10.1002/cbdv.201200289.

(69) Wirth, T.; Schmuck, K.; Tietze, L. F.; Sieber, S. A. Duocarmycin Analogues Target Aldehyde Dehydrogenase 1 in Lung Cancer Cells. *Angew. Chem. Int. Ed.* **2012**, *51* (12), 2874–2877. https://doi.org/10.1002/anie.201106334.

(70) Wolfe, A. L.; Duncan, K. K.; Parelkar, N. K.; Weir, S. J.; Vielhauer, G. A.; Boger, D. L. A Novel, Unusually Efficacious Duocarmycin Carbamate Prodrug That Releases No Residual Byproduct. *J. Med. Chem.* **2012**, *55* (12), 5878–5886. https://doi.org/10.1021/jm300330b.

(71) Boger, D. L. Cyclic Prodrugs of Duocarmycin Analogs. WO 2013/148631 A1, October 3, 2013.

(72) Chang, J. Y.-C.; Lu, G.-L.; Stevenson, R. J.; Brothers, P. J.; Clark, G. R.; Botting, K. J.; Ferry, D. M.; Tercel, M.; Wilson, W. R.; Denny, W. A.; Ware, D. C. Cross-Bridged Cyclen or Cyclam Co(III) Complexes Containing Cytotoxic Ligands as Hypoxia-Activated Prodrugs. *Inorg. Chem.* **2013**, *52* (13), 7688–7698. https://doi.org/10.1021/ic4006967.

(73) Chen, K.-C.; Schmuck, K.; Tietze, L. F.; Roffler, S. R. Selective Cancer Therapy by Extracellular Activation of a Highly Potent Glycosidic Duocarmycin Analogue. *Mol. Pharm.* **2013**, *10* (5), 1773–1782. https://doi.org/10.1021/mp300581u.

(74) Sheldrake, H. M.; Travica, S.; Johansson, I.; Loadman, P. M.; Sutherland, M.; Elsalem, L.; Illingworth, N.; Cresswell, A. J.; Reuillon, T.; Shnyder, S. D.; Mkrtchian, S.; Searcey, M.; Ingelman-Sundberg, M.; Patterson, L. H.; Pors, K. Re-Engineering of the Duocarmycin Structural Architecture Enables Bioprecursor Development Targeting CYP1A1 and CYP2W1 for Biological Activity. *J. Med. Chem.* **2013**, *56* (15), 6273–6277. https://doi.org/10.1021/jm4000209.

(75) Tietze, L. F.; Müller, M.; Duefert, S.-C.; Schmuck, K.; Schuberth, I. Photoactivatable Prodrugs of Highly Potent Duocarmycin Analogues for a Selective Cancer Therapy. *Chem. Eur. J.* **2013**, *19* (5), 1726–1731. https://doi.org/10.1002/chem.201202773.

(76) Tietze, L. F.; Sieber, S. A. Duocarmycin Analogues without a DNA-Binding Indole Unit Associate with Aldehyde Dehydrogenase 1A1 and Not DNA: A Reply. *Angew. Chem. Int. Ed.* **2013**, *52* (21), 5447–5449. https://doi.org/10.1002/anie.201301923.

(77) Travica, S.; Pors, K.; Loadman, P. M.; Shnyder, S. D.; Johansson, I.; Alandas, M. N.; Sheldrake, H. M.; Mkrtchian, S.; Patterson, L. H.; Ingelman-Sundberg, M. Colon Cancer–Specific Cytochrome P450 2W1 Converts Duocarmycin Analogues into Potent Tumor Cytotoxins. *Clin. Cancer Res.* **2013**, *19* (11), 2952–2961. https://doi.org/10.1158/1078-0432.CCR-13-0238.

(78) Vielhauer, G. A.; Swink, M.; Parelkar, N. K.; Lajiness, J. P.; Wolfe, A. L.; Boger, D. Evaluation of a Reductively Activated Duocarmycin Prodrug against Murine and Human Solid Cancers. *Cancer Biol. & Ther.* **2013**, *14* (6), 527–536. https://doi.org/10.4161/cbt.24348.

(79) Wolfe, A. L.; Duncan, K. K.; Parelkar, N. K.; Brown, D.; Vielhauer, G. A.; Boger, D. L. Efficacious Cyclic *N* -Acyl *O* -Amino Phenol Duocarmycin Prodrugs. *J. Med. Chem.* **2013**, *56* (10), 4104–4115. https://doi.org/10.1021/jm400413r.

(80) Hunter, F. W.; Jaiswal, J. K.; Hurley, D. G.; Liyanage, H. D. S.; McManaway, S. P.; Gu, Y.; Richter, S.; Wang, J.; Tercel, M.; Print, C. G.; Wilson, W. R.; Pruijn, F. B. The Flavoprotein FOXRED2 Reductively Activates Nitro-Chloromethylbenzindolines and Other Hypoxia-Targeting Prodrugs. *Biochemical Pharmacology* **2014**, *89* (2), 224–235. https://doi.org/10.1016/j.bcp.2014.03.001.

(81) Krall, N.; Pretto, F.; Decurtins, W.; Bernardes, G. J. L.; Supuran, C. T.; Neri, D. A Small-Molecule Drug Conjugate for the Treatment of Carbonic Anhydrase IX Expressing Tumors. *Angew. Chem. Int. Ed.* **2014**, *53* (16), 4231–4235. https://doi.org/10.1002/anie.201310709.

(82) Tercel, M.; McManaway, S. P.; Liyanage, H. D. S.; Pruijn, F. B. Preparation and Properties of Clickable Amino Analogues of the Duocarmycins: Factors That Affect the Efficiency of Their Fluorescent Labelling of DNA. *ChemMedChem* **2014**, *9* (9), 2193–2206. https://doi.org/10.1002/cmdc.201402169.

(83) Uematsu, M.; Boger, D. L. Asymmetric Synthesis of a CBI-Based Cyclic *N* -Acyl *O* -Amino Phenol Duocarmycin Prodrug. *J. Org. Chem.* **2014**, *79* (20), 9699–9703. https://doi.org/10.1021/jo501839x.

(84) Beusker, P. H. Water-Soluble CC-1065 Analogs and Their Conjugates. US008940784B2, January 27, 2015.

(85) Koch, M. F.; Harteis, S.; Blank, I. D.; Pestel, G.; Tietze, L. F.; Ochsenfeld, C.; Schneider, S.; Sieber, S. A. Structural, Biochemical, and Computational Studies Reveal the Mechanism of Selective Aldehyde Dehydrogenase 1A1 Inhibition by Cytotoxic Duocarmycin Analogues. *Angew. Chem. Int. Ed.* **2015**, *54* (46), 13550–13554. https://doi.org/10.1002/anie.201505749.

(86) Uematsu, M.; Brody, D. M.; Boger, D. L. A Five-Membered Lactone Prodrug of CBI-Based Analogs of the Duocarmycins. *Tet. Lett.* **2015**, *56* (23), 3101–3104. https://doi.org/10.1016/j.tetlet.2014.11.038.

(87) Giddens, A. C.; Lee, H. H.; Lu, G.-L.; Miller, C. K.; Guo, J.; Lewis Phillips, G. D.; Pillow, T. H.; Tercel, M. Analogues of DNA Minor Groove Cross-Linking Agents Incorporating AminoCBI, an Amino Derivative of the Duocarmycins: Synthesis, Cytotoxicity, and Potential as Payloads for Antibody–Drug Conjugates. *Bioorg. Med. Chem.* **2016**, *24* (22), 6075–6081. https://doi.org/10.1016/j.bmc.2016.09.068.

(88) Spangler, B.; Fontaine, S. D.; Shi, Y.; Sambucetti, L.; Mattis, A. N.; Hann, B.; Wells, J. A.; Renslo, A. R. A Novel Tumor-Activated Prodrug Strategy Targeting Ferrous Iron Is Effective in Multiple Preclinical Cancer Models. *J. Med. Chem.* **2016**, *59* (24), 11161–11170. https://doi.org/10.1021/acs.jmedchem.6b01470.

(89) Boger, D. L. Cyclic N-Acyl O-Amino Phenol CBI Derivative. US009586974B2, March 7, 2017.

(90) Jiménez-Moreno, E.; Guo, Z.; Oliveira, B. L.; Albuquerque, I. S.; Kitowski, A.; Guerreiro, A.; Boutureira, O.; Rodrigues, T.; Jiménez-Osés, G.; Bernardes, G. J. L. Vinyl Ether/Tetrazine Pair for the Traceless Release of Alcohols in Cells. *Angew. Chem. Int. Ed.* **2017**, *129* (1), 249–253. https://doi.org/10.1002/ange.201609607.

(91) Tercel, M.; Lee, H. H.; Mehta, S. Y.; Youte Tendoung, J.-J.; Bai, S. Y.; Liyanage, H. D. S.; Pruijn, F. B. Influence of a Basic Side Chain on the Properties of Hypoxia-

Selective Nitro Analogues of the Duocarmycins: Demonstration of Substantial Anticancer Activity in Combination with Irradiation or Chemotherapy. *J. Med. Chem.* **2017**, *60* (13), 5834–5856. https://doi.org/10.1021/acs.jmedchem.7b00563.

(92) Tietze, L. F.; Penchalaiah, K. Bifunctional Prodrugs. WO 2017/072295A1, May 4, 2017.

(93) Beekman, A. M.; Cominetti, M. M. D.; Cartwright, O. C.; Boger, D. L.; Searcey, M. A Small Molecule Drug Conjugate (SMDC) of DUPA and a Duocarmycin Built on the Solid Phase. *Med. Chem. Commun.* **2019**, *10* (12), 2170–2174. https://doi.org/10.1039/C9MD00279K.

(94) Lee, H. H.; Dickson, B. D.; Stevenson, R. J.; Yang, S.; Tercel, M. Optimised Synthesis of a NitroCBI Hypoxia-Activated Prodrug with Substantial Anticancer Activity. *Tetrahedron* **2019**, *75* (22), 3001–3007. https://doi.org/10.1016/j.tet.2019.04.027.

(95) Cartwright, O. C.; Beekman, A. M.; Cominetti, M. M. D.; Russell, D. A.; Searcey, M. A Peptide–Duocarmycin Conjugate Targeting the Thomsen-Friedenreich Antigen Has Potent and Selective Antitumor Activity. *Bioconjugate Chem.* **2020**, *31* (7), 1745–1749. https://doi.org/10.1021/acs.bioconjchem.0c00282.

(96) Guerrero, A.; Guiho, R.; Herranz, N.; Uren, A.; Withers, D. J.; Martínez-Barbera, J. P.; Tietze, L. F.; Gil, J. Galactose-modified Duocarmycin Prodrugs as Senolytics. *Aging Cell* **2020**, *19* (4). https://doi.org/10.1111/acel.13133.

(97) Hong, C. R.; Mehta, S. Y.; Liyanage, H. D. S.; McManaway, S. P.; Lee, H. H.; Jaiswal, J. K.; Bogle, G.; Tercel, M.; Pruijn, F. B.; Wilson, W. R.; Hicks, K. O. Spatially-Resolved Pharmacokinetic/Pharmacodynamic Modelling of Bystander Effects of a Nitrochloromethylbenzindoline Hypoxia-Activated Prodrug. *Cancer Chemother. Pharmacol.* **2021**, *88* (4), 673–687. https://doi.org/10.1007/s00280-021-04320-3.

(98) Sharrock, A. V.; McManaway, S. P.; Rich, M. H.; Mumm, J. S.; Hermans, I. F.; Tercel, M.; Pruijn, F. B.; Ackerley, D. F. Engineering the Escherichia Coli Nitroreductase NfsA to Create a Flexible Enzyme-Prodrug Activation System. *Front. Pharmacol.* **2021**, *12*.

(99) Thorn-Seshold, O.; Felber, J.; Thorn-Seshold, J.; Zeisel, L. Disulfide-Based Prodrug Compounds. PCT/EP2022/057483, 2021.

(100) Thorn-Seshold, O.; Zeisel, L.; Felber, J. G. Dichalcogenide Prodrugs. PCT/EP2022/059280, 2021.

(101) Bart, A. G.; Morais, G.; Vangala, V. R.; Loadman, P. M.; Pors, K.; Scott, E. E. Cytochrome P450 Binding and Bioactivation of Tumor-Targeted Duocarmycin Agents. *Drug Metab Dispos* **2022**, *50* (1), 49–57. https://doi.org/10.1124/dmd.121.000642.

(102) Felber, J. G.; Kitowski, A.; Zeisel, L.; Maier, M. S.; Heise, C.; Thorn-Seshold, J.; Thorn-Seshold, O. Cancer Prodrug Activation through Trx/TrxR-Selective Bioreduction of Monothiol-Stable Seco-Duocarmycins in Vivo. *in preparation* **2022**.

(3) duocarmycin ADCs (72 items - 39 journal publications, 33 patents)

(1) Chari, R. V. J.; Jacket, K. A.; Bourret, L. A.; Pullen, S. M.; Tadayoni, B. M.; Mattocks, K. M.; Shah, S. A.; Liu, C.; Blaettler, W. A.; Goldmacher, V. S. Enhancement of the Selectivity and Antitumor Efficacy of a CC-1065 Analogue through Immunoconjugate Formation. *Cancer Res.* **1995**, *55*, 4079–4084.

(2) Chari, R. V. J.; Goldmacher, V. S.; Blaettler, W. A. Cell Binding Agent Conjugates of Analogues and Derivatives of CC-1065. US005475092A, December 12, 1995.

(3) Chari, R. V. J.; Goldmacher, V. S.; Blaettler, W. A. Cyclopropylbenzindole-Containing Cytotoxic Drugs. US005585499A, December 17, 1996.

(4) Chari, R. V. J.; Goldmacher, V. S.; Blaettler, W. A. Targeted Delivery of Cyclopropylbenzindole-Containing Cytotoxic Drugs. US005846545A, December 8, 1998.

(5) Kelly, R. C.; Mitchell, M. A.; Aristoff, P. A. CC-1065 Analogs. US005739350A, April 14, 1998.

(6) Suzawa, T.; Nagamura, S.; Saito, H.; Ohta, S.; Hanai, N.; Yamasaki, M. Synthesis and HPLC Analysis of Enzymatically Cleavable Linker Consisting of Poly(Ethylene Glycol) and Dipeptide for the Development of Immunoconjugate. *J. Control. Rel.* **2000**, *69* (1), 27–41. https://doi.org/10.1016/S0168-3659(00)00282-0.

(7) Suzawa, T.; Nagamura, S.; Saito, H.; Ohta, S.; Hanai, N.; Yamasaki, M. Synthesis of a Novel Duocarmycin Derivative DU-257 and Its Application to Immunoconjugate Using Poly(Ethylene Glycol)- Dipeptidyl Linker Capable of Tumor Specific Activation. *Bioorg. Med. Chem.* **2000**, *8*, 2175–2184. https://doi.org/10.1016/s0968-0896(00)00157-7.

(8) Lillo, A. M.; Sun, C.; Gao, C.; Ditzel, H.; Parrish, J.; Gauss, C.-M.; Moss, J.; Felding-Habermann, B.; Wirsching, P.; Boger, D. L.; Janda, K. D. A Human Single-Chain Antibody Specific for Integrin Alpha1beta2 Capable of Cell Internalization and Delivery of Antitumor Agents. *Chem. & Biol.* **2004**, *11*, 897–906. https://doi.org/10.1016/j.chembiol.2004.04.018.

(9) Jeffrey, S. C.; Torgov, M. Y.; Andreyka, J. B.; Boddington, L.; Cerveny, C. G.; Denny, W. A.; Gordon, K. A.; Gustin, D.; Haugen, J.; Kline, T.; Nguyen, M. T.; Senter, P. D. Design, Synthesis, and in Vitro Evaluation of Dipeptide-Based Antibody Minor Groove Binder Conjugates. *J. Med. Chem.* **2005**, *48* (5), 1344–1358. https://doi.org/10.1021/jm040137q.

(10) Boyd, S. E.; Chen, L.; Gangwar, S.; Guerlavais, V.; Horgan, K.; Sufi, B.; Cardarelli, J. M.; Pan, C.; Huang, H.; King, D. J. Conjugates of Duocarmycin and Anti-CD70 or Anti-PSMA Antibodies. EP 2354163A2, September 26, 2006.

(11) Gangwar, S.; Sufi, B. Cytotoxic Compounds and Conjugates with Cleavable Substrates. US 20060247295A1, November 2, 2006.

(12) Janda, K. D.; Wirsching, P.; Boger, D. L. Compositions and Methods for Delivery of Antitumor Agents. WO 2006/002895A2, January 12, 2006.

(13) Boyd, S. E.; Chen, L.; Gangwar, S.; Guerlavais, V.; Horgan, K.; Sufi, B.; Huang, H.; King, D. J.; Pan, C.; Cardarelli, J. M. Antibody-Drug Conjugates and Methods of Use. US20080279868A1, November 13, 2008.

(14) Chen, L.; Gangwar, S.; Guerlavais, V.; Lonberg, N.; Zhang, Q. Chemical Linkers with Single Amino Acids and Conjugates Thereof. US20100113476A1, May 6, 2010.

(15) Sufi, B.; Guerlavais, V.; Chen, L.; Gangwar, S.; Zhang, Q.; Passmore, D. B. Chemical Linkers and Cleavable Substrates and Conjugates Thereof. US20100145036A1, June 10, 2010.

(16) Gangwar, S.; Sufi, B. Cytotoxic Compounds and Conjugates. US 007968586B2, June 28, 2011.

(17) Zhao, R. Y.; Erickson, H. K.; Leece, B. A.; Reid, E. E.; Goldmacher, V. S.; Lambert, J. M.; Chari, R. V. J. Synthesis and Biological Evaluation of Antibody Conjugates of Phosphate Prodrugs of Cytotoxic DNA Alkylators for the Targeted Treatment of Cancer. *J. Med. Chem.* **2012**, *55* (2), 766–782. https://doi.org/10.1021/jm201284m.

(18) Beusker, P. H.; Coumans, R. G. E.; Elgersma, R. C.; Menge, W. M. P. B.; Joosten, A. F.; Spijker, H. J.; de Groot, F. M. H. Novel Conjugates of CC-1065 Analogs and Bifunctional Linkers. US20130224227A1, August 29, 2013.

(19) Thevanayagam, L.; Bell, A.; Chakraborty, I.; Sufi, B.; Gangwar, S.; Zang, A.; Rangan, V.; Rao, C.; Wang, Z.; Pan, C.; Chong, C.; Cardarelli, P.; Deshpande, S.; Srinivasan, M. Novel Detection of DNA-Alkylated Adducts of Antibody–Drug Conjugates with Potentially Unique Preclinical and Biomarker Applications. *Bioanalysis* **2013**, *5* (9), 1073–1081. https://doi.org/10.4155/bio.13.57.

(20) Beusker, P. H.; Coumans, R. G. E.; Elgersma, R. C.; Menge, W. M. P. B.; Joosten, J. A. F.; Spijker, H. J.; de Groot, F. M. H. CC-1065 Analogs and Their Conjugates. US008889868B2, November 18, 2014.

(21) Beusker, P. H.; Spijker, H. J.; Joosten, A. F.; Huijbregts, T.; de Groot, F. M. H. Substituted CC-1065 Analogs and Their Conjugates. US008680293B2, March 25, 2014.

(22) Dokter, W.; Ubink, R.; van der Lee, M.; van der Vleuten, M.; van Achterberg, T.; Jacobs, D.; Loosveld, E.; van den Dobbelsteen, D.; Egging, D.; Mattaar, E.; Groothuis, P.; Beusker, P.; Coumans, R.; Elgersma, R.; Menge, W.; Joosten, J.; Spijker, H.; Huijbregts, T.; de Groot, V.; Eppink, M.; de Roo, G.; Verheijden, G.; Timmers, M. Preclinical Profile of the HER2-Targeting ADC SYD983/SYD985: Introduction of a New Duocarmycin-Based Linker-Drug Platform. *Mol. Cancer Ther.* **2014**, *13* (11), 2618–2629. https://doi.org/10.1158/1535-7163.MCT-14-0040-T.

(23) Perrino, E.; Steiner, M.; Krall, N.; Bernardes, G. J. L.; Pretto, F.; Casi, G.; Neri, D. Curative Properties of Noninternalizing Antibody–Drug Conjugates Based on Maytansinoids. *Cancer Res.* **2014**, *74* (9), 2569–2578. https://doi.org/10.1158/0008-5472.CAN-13-2990.

(24) Elgersma, R. C.; Coumans, R. G. E.; Huijbregts, T.; Menge, W. M. P. B.; Joosten, J. A. F.; Spijker, H. J.; de Groot, F. M. H.; van der Lee, M. M. C.; Ubink, R.; van den Dobbelsteen, D. J.; Egging, D. F.; Dokter, W. H. A.; Verheijden, G. F. M.; Lemmens, J. M.; Timmers, C. M.; Beusker, P. H. Design, Synthesis, and Evaluation of Linker-Duocarmycin Payloads: Toward Selection of HER2-Targeting Antibody–Drug Conjugate SYD985. *Mol. Pharmaceutics* **2015**, *12* (6), 1813–1835. https://doi.org/10.1021/mp500781a.

(25) Flygare, J. A.; Pillow, T. H.; Safina, B.; Verma, V.; Wei, B.; Denny, W. A.; Giddens, A. C.; Lee, H.; Lu, G.-L.; Miller, C.; Rewcastle, G.; Tercel, M.; Bonnet, M. 1-(Chloromethyl)-2,3-Dihydro-1H-Benzo[e]Indole Dimer Antibody-Drug Conjugate Compounds, and Methods of Use and Treatment. US20150165063A1, June 18, 2015.

(26) Maderna, A.; Doroski, M. D.; Chen, Z.; Risley, H. L.; Casavant, J. M.; O'Donnell, C. J.; Porte, A. M.; Subramanyam, C. Bifunctional Cytotoxic Agents. US20150209445A1, July 30, 2015.

(27) van der Lee, M. M. C.; Groothuis, P. G.; Ubink, R.; van der Vleuten, M. A. J.; van Achterberg, T. A.; Loosveld, E. M.; Damming, D.; Jacobs, D. C. H.; Rouwette, M.; Egging, D. F.; van den Dobbelsteen, D.; Beusker, P. H.; Goedings, P.; Verheijden, G. F. M.; Lemmens, J. M.; Timmers, M.; Dokter, W. H. A. The Preclinical Profile of the Duocarmycin-Based HER2-Targeting ADC SYD985 Predicts for Clinical Benefit in Low HER2-Expressing Breast Cancers. *Mol. Cancer Ther.* **2015**, *14* (3), 692–703. https://doi.org/10.1158/1535-7163.MCT-14-0881-T.

(28) Beusker, P. H.; Coumans, R. G. E.; Elgersma, R. C.; Menge, W. M. P. B.; Joosten, A. F.; Spijker, H. J.; de Groot, F. M. H. Novel CC-1065 Analogs and Their Conjugates. US20160052880A1, February 25, 2016.

(29) Black, J.; Menderes, G.; Bellone, S.; Schwab, C. L.; Bonazzoli, E.; Ferrari, F.; Predolini, F.; De Haydu, C.; Cocco, E.; Buza, N.; Hui, P.; Wong, S.; Lopez, S.; Ratner, E.; Silasi, D.-A.; Azodi, M.; Litkouhi, B.; Schwartz, P. E.; Goedings, P.; Beusker, P. H.; van der Lee, M. M. C.; Timmers, C. M.; Dokter, W. H. A.; Santin, A. D. SYD985, a Novel Duocarmycin-Based HER2-Targeting Antibody–Drug Conjugate, Shows Antitumor Activity in Uterine Serous Carcinoma with HER2/Neu Expression. *Mol. Cancer Ther.* **2016**, *15* (8), 1900–1909. https://doi.org/10.1158/1535-7163.MCT-16-0163.

(30) Chen, X.; Dennis, M.; Junutula, J. R.; Phillips, G. L.; Pillow, T. H.; Sliwkowski, M. X. Anti-Her2 Antibodies and Immunoconjugates. US20160096893A1, April 7, 2016.

(31) Dokter, W.; Goedings, P. J.; Verheijden, G. F. M.; Beusker, P. H. Duocarmycin ADCs Showing Improved in Vivo Antitumor Activity. US20160008486A1, January 14, 2016.

(32) Lin, R.-H.; Lin, S.-Y.; Hsieh, Y.-C.; Huang, C.-C.; Lee, S.-H.; Tsai, Y.-Y. Her2 Antibody-Drug Conjugates. US20160051695A1, February 25, 2016.

(33) Maderna, A.; Subramanyam, C.; Tumey, L. N.; Chen, Z.; Casavant, J. M. Bifunctional Cytotoxic Agents Containing the CTI Pharmacophore. WO 2016/151432A1, September 29, 2016.

(34) Owonikoko, T. K.; Hussain, A.; Stadler, W. M.; Smith, D. C.; Kluger, H.; Molina, A. M.; Gulati, P.; Shah, A.; Ahlers, C. M.; Cardarelli, P. M.; Cohen, L. J. First-in-Human Multicenter Phase I Study of BMS-936561 (MDX-1203), an Antibody-Drug Conjugate Targeting CD70. *Cancer Chemother. Pharmacol.* **2016**, *77* (1), 155–162. https://doi.org/10.1007/s00280-015-2909-2.

(35) Santin, A. D.; Goedings, P. Duocarmycin ADCs for Use in Treatment of Endometrial Cancer. 009427480B2, August 30, 2016.

(36) Wang, H.; Rangan, V. S.; Sung, M.-C.; Passmore, D.; Kempe, T.; Wang, X.; Thevanayagam, L.; Pan, C.; Rao, C.; Srinivasan, M.; Zhang, Q.; Gangwar, S.; Deshpande, S.; Cardarelli, P.; Marathe, P.; Yang, Z. Pharmacokinetic Characterization of BMS-936561, an Anti-CD70 Antibody-Drug Conjugate, in Preclinical Animal Species and Prediction of Its Pharmacokinetics in Humans: PHARMACOKINETICS OF ***α*** CD70_MED-A. *Biopharm. Drug Dispos.* **2016**, *37* (2), 93–106. https://doi.org/10.1002/bdd.1953.

(37) Capone, E.; Piccolo, E.; Fichera, I.; Ciufici, P.; Barcaroli, D.; Sala, A.; De Laurenzi, V.; Iacobelli, V.; Iacobelli, S.; Sala, G. Generation of a Novel Antibody-Drug Conjugate Targeting Endosialin: Potent and Durable Antitumor Response in Sarcoma. *Oncotarget* **2017**, *8* (36), 60368–60377. https://doi.org/10.18632/oncotarget.19499.

(38) Egging, D.; Beusker, P. H.; Mattaar, E.; Bos, E. S. Pan-Reactive Antibodies to Duocarmycins. US20170320965A1, November 9, 2017.

(39) Huijbregts, T.; Elgersma, R. C.; Beusker, P. H.; Joosten, A. F.; Coumans, R. G. E.; Spijker, H. J.; Menge, W.; de Groot, F. M. H. Process for Making Duocarmycin Prodrugs. US20170145006A1, May 25, 2017.

(40) Iacobelli, S.; Di Risio, A.; Piccolo, E.; Sala, G.; Capone, E. Endosialin-Binding Antibody. WO 2017/134234A1, August 10, 2017.

(41) Menderes, G.; Bonazzoli, E.; Bellone, S.; Black, J.; Altwerger, G.; Masserdotti, A.; Pettinella, F.; Zammataro, L.; Buza, N.; Hui, P.; Wong, S.; Litkouhi, B.; Ratner, E.; Silasi, D.-A.; Huang, G. S.; Azodi, M.; Schwartz, P. E.; Santin, A. D. SYD985, a Novel Duocarmycin-Based HER2-Targeting Antibody-Drug Conjugate, Shows Promising Antitumor Activity in Epithelial Ovarian Carcinoma with HER2/Neu Expression. *Gynecologic Oncology* **2017**, *146* (1), 179–186. https://doi.org/10.1016/j.ygyno.2017.04.023.

(42) Menderes, G.; Bonazzoli, E.; Bellone, S.; Black, J.; Predolini, F.; Pettinella, F.; Masserdotti, A.; Zammataro, L.; Altwerger, G.; Buza, N.; Hui, P.; Wong, S.; Litkouhi, B.; Ratner, E.; Silasi, D.-A.; Azodi, M.; Schwartz, P. E.; Santin, A. D. SYD985, a Novel Duocarmycin-Based HER2-Targeting Antibody–Drug Conjugate, Shows Antitumor Activity in Uterine and Ovarian Carcinosarcoma with HER2/Neu Expression. *Clin. Cancer Res.* **2017**, *23* (19), 5836–5845. https://doi.org/10.1158/1078-0432.CCR-16-2862.

(43) Nani, R. R.; Gorka, A. P.; Nagaya, T.; Yamamoto, T.; Ivanic, J.; Kobayashi, H.; Schnermann, M. J. *In Vivo* Activation of Duocarmycin–Antibody Conjugates by Near-Infrared Light. *ACS Cent. Sci.* **2017**, *3* (4), 329–337. https://doi.org/10.1021/acscentsci.7b00026.

(44) Osinga, N. J.; Boxmeer Van, E. J. B. Composition Comprising Antibody-Duocarmycin Drug Conjugates. WO 2017/009255A1, January 19, 2017.

(45) Pillow, T. H.; Sadowsky, J. D.; Zhang, D.; Yu, S.-F.; Del Rosario, G.; Xu, K.; He, J.; Bhakta, S.; Ohri, R.; Kozak, K. R.; Ha, E.; Junutula, J. R.; Flygare, J. A. Decoupling Stability and Release in Disulfide Bonds with Antibody-Small Molecule Conjugates. *Chem. Sci.* **2017**, *8* (1), 366–370. https://doi.org/10.1039/C6SC01831A.

(46) FDA Updates. Fast Track Designation Granted for Breast Cancer Treatment. *Oncology Times* **2018**, *40* (5), 16. https://doi.org/10.1097/01.COT.0000531194.80509.6d.

(47) Helin, J.; Saarinen, J.; Satomaa, T.; Ekholm, F. S. Saccaride Derivative of a Toxic Payload and Antibody Conjugates Thereof. US20180228906A1, August 16, 2018.

(48) Jin, J.; Park, G.; Park, J. B.; Kim, S.; Kim, H.; Chung, J. An Anti-EGFR × Cotinine Bispecific Antibody Complexed with Cotinine-Conjugated Duocarmycin Inhibits Growth of EGFR-Positive Cancer Cells with KRAS Mutations. *Exp. Mol. Med.* **2018**, *50* (5), 1–14. https://doi.org/10.1038/s12276-018-0096-z.

(49) Junutula, J. R.; Smith, S. W.; Borkin, D.; Degrado, S. Isoquinolidinobenzodiazepine (ICQ) - 1-(Chloromethyl)-2,3-Dihydro-1H-Benzo8e9indole (CBI) Dimers. Wo 2018/071455 A1, April 19, 2018.

(50) Lütje, S.; Gerrits, D.; Molkenboer-Kuenen, J. D.; Herrmann, K.; Fracasso, G.; Colombatti, M.; Boerman, O. C.; Heskamp, S. Characterization of Site-Specifically Conjugated Monomethyl Auristatin E– and Duocarmycin-Based Anti-PSMA Antibody–Drug Conjugates for Treatment of PSMA-Expressing Tumors. *J. Nucl. Med.* **2018**, *59* (3), 494–501. https://doi.org/10.2967/jnumed.117.196279.

(51) Nagaya, T.; Gorka, A. P.; Nani, R. R.; Okuyama, S.; Ogata, F.; Maruoka, Y.; Choyke, P. L.; Schnermann, M. J.; Kobayashi, H. Molecularly Targeted Cancer Combination Therapy with Near-Infrared Photoimmunotherapy and Near-Infrared Photorelease with Duocarmycin–Antibody Conjugate. *Mol. Cancer Ther.* **2018**, *17* (3), 661–670. https://doi.org/10.1158/1535-7163.MCT-17-0851.

(52) Ubink, R.; Dirksen, E. H. C.; Rouwette, M.; Bos, E. S.; Janssen, I.; Egging, D. F.; Loosveld, E. M.; van Achterberg, T. A.; Berentsen, K.; van der Lee, M. M. C.; Bichat, F.; Raguin, O.; van der Vleuten, M. A. J.; Groothuis, P. G.; Dokter, W. H. A. Unraveling the Interaction between Carboxylesterase 1c and the Antibody–Drug Conjugate SYD985: Improved Translational PK/PD by Using Ces1c Knockout Mice. *Mol. Cancer Ther.* **2018**, *17* (11), 2389–2398. https://doi.org/10.1158/1535-7163.MCT-18-0329.

(53) Yu, L.; Lu, Y.; Yao, Y.; Liu, Y.; Wang, Y.; Lai, Q.; Zhang, R.; Li, W.; Wang, R.; Fu, Y.; Tao, Y.; Yi, S.; Gou, L.; Chen, L.; Yang, J. Promiximab-Duocarmycin, a New CD56 Antibody-Drug Conjugates, Is Highly Efficacious in Small Cell Lung Cancer Xenograft Models. *Oncotarget* **2018**, *9* (4), 5197–5207. https://doi.org/10.18632/oncotarget.23708.

(54) Zhang, D.; Le, H.; Cruz-Chuh, J. dela; Bobba, S.; Guo, J.; Staben, L.; Zhang, C.; Ma, Y.; Kozak, K. R.; Lewis Phillips, G. D.; Vollmar, B. S.; Sadowsky, J. D.; Vandlen, R.; Wei, B.; Su, D.; Fan, P.; Dragovich, P. S.; Khojasteh, S. C.; Hop, C. E. C. A.; Pillow, T. H. Immolation of *p* -Aminobenzyl Ether Linker and Payload Potency and Stability Determine the Cell-Killing Activity of Antibody–Drug Conjugates with Phenol-Containing Payloads. *Bioconjugate Chem.* **2018**, *29* (2), 267–274. https://doi.org/10.1021/acs.bioconjchem.7b00576.

(55) Ariaans, G. J. A.; Coumans, R. G. E. Site-Specific Conjugation of Linker Drugs to Antibodies and Resulting ADCs. US010407743B2, September 10, 2019.

(56) Banerji, U.; van Herpen, C. M. L.; Saura, C.; Thistlethwaite, F.; Lord, S.; Moreno, V.; Macpherson, I. R.; Boni, V.; Rolfo, C.; de Vries, E. G. E.; Rottey, S.; Geenen, J.; Eskens, F.; Gil-Martin, M.; Mommers, E. C.; Koper, N. P.; Aftimos, P. Trastuzumab Duocarmazine in Locally Advanced and Metastatic Solid Tumours and HER2-Expressing Breast Cancer: A Phase 1 Dose-Escalation and Dose-Expansion Study. *The Lancet Oncology* **2019**, *20* (8), 1124–1135. https://doi.org/10.1016/S1470-2045(19)30328-6.

(57) Fu, Y.; Urban, D. J.; Nani, R. R.; Zhang, Y.-F.; Li, N.; Fu, H.; Shah, H.; Gorka, A. P.; Guha, R.; Chen, L.; Hall, M. D.; Schnermann, M. J.; Ho, M. Glypican-3-Specific Antibody Drug Conjugates Targeting Hepatocellular Carcinoma. *Hepatology* **2019**, *70* (2), 563–576. https://doi.org/10.1002/hep.30326.

(58) Jackson, P. M. J.; Thurston, D. E.; Rahman, K. M. Asymmetric Conjugate Compounds. US20190144443A1, May 16, 2019.

(59) Kim, S.; Kim, H.; Jo, D. H.; Kim, J. H.; Kim, S. R.; Kang, D.; Hwang, D.; Chung, J. Bispecific Anti-MPDGFRβ x Cotinine ScFv-Cκ-ScFv Fusion Protein and Cotinine-Duocarmycin Can Form Antibody-Drug Conjugate-like Complexes That Exert Cytotoxicity against MPDGFRβ Expressing Cells. *Methods* **2019**, *154*, 125–135. https://doi.org/10.1016/j.ymeth.2018.10.002.

(60) Loo, D. T.; Huang, L.; Johnson, L. S.; Son, T.; Scribner, J. A.; Bonvini, E. Novel B7-H3 Binding Molecules, Antibody Drug Conjugates Thereof and Methods of Use Thereof. US2019012471A1, May 2, 2019.

(61) Rinnerthaler, G.; Gampenrieder, S.; Greil, R. HER2 Directed Antibody-Drug-Conjugates beyond T-DM1 in Breast Cancer. *Int. J. Mol. Sci.* **2019**, *20* (5), 1115. https://doi.org/10.3390/ijms20051115.

(62) Su, D.; Chen, J.; Cosino, E.; dela Cruz-Chuh, J.; Davis, H.; Del Rosario, G.; Figueroa, I.; Goon, L.; He, J.; Kamath, A. V.; Kaur, S.; Kozak, K. R.; Lau, J.; Lee, D.; Lee, M. V.; Leipold, D.; Liu, L.; Liu, P.; Lu, G.-L.; Nelson, C.; Ng, C.; Pillow, T. H.; Polakis, P.; Polson, A. G.; Rowntree, R. K.; Saad, O.; Safina, B.; Stagg, N. J.; Tercel, M.; Vandlen, R.; Vollmar, B. S.; Wai, J.; Wang, T.; Wei, B.; Xu, K.; Xue, J.; Xu, Z.; Yan, G.; Yao, H.; Yu, S.-F.; Zhang, D.; Zhong, F.; Dragovich, P. S. Antibody–Drug Conjugates Derived from Cytotoxic *Seco* -CBI-Dimer Payloads Are Highly Efficacious in Xenograft Models and Form Protein Adducts *In Vivo*. *Bioconjugate Chem.* **2019**, *30* (5), 1356–1370. https://doi.org/10.1021/acs.bioconjchem.9b00133.

(63) Tietze, L. F. Method for the Synthesis of Monoprotected Bifunctional Prodrugs and Antibody Drug Conjugates Based Thereon as Well as a Method for Preparing Antibody Drug Conjugates. WO 2019/030367A1, February 14, 2019.

(64) Tong, X.-M.; Feng, L.; Suthe, S. R.; Weng, T.-H.; Hu, C.-Y.; Liu, Y.-Z.; Wu, Z.-G.; Wang, M.-H.; Yao, H.-P. Therapeutic Efficacy of a Novel Humanized Antibody-Drug Conjugate Recognizing Plexin-Semaphorin-Integrin Domain in the RON Receptor for Targeted Cancer Therapy. *J. Immunotherapy Cancer* **2019**, *7* (1), 250. https://doi.org/10.1186/s40425-019-0732-8.

(65) Coumans, R. G. E.; Ariaans, G. J. A.; Spijker, H. J.; Renart Verkerk, P.; Beusker, P. H.; Kokke, B. P. A.; Schouten, J.; Blomenröhr, M.; van der Lee, M. M. C.; Groothuis, P. G.; Ubink, R.; Dokter, W. H. A.; Timmers, C. M. A Platform for the Generation of Site-Specific Antibody–Drug Conjugates That Allows for Selective Reduction of Engineered Cysteines. *Bioconjugate Chem.* **2020**, *31* (9), 2136–2146. https://doi.org/10.1021/acs.bioconjchem.0c00337.

(66) Hackshaw, M. D.; Danysh, H. E.; Singh, J.; Ritchey, M. E.; Ladner, A.; Taitt, C.; Camidge, D. R.; Iwata, H.; Powell, C. A. Incidence of Pneumonitis/Interstitial Lung Disease Induced by HER2-Targeting Therapy for HER2-Positive Metastatic Breast Cancer. *Breast Cancer Res. Treat.* **2020**, *183* (1), 23–39. https://doi.org/10.1007/s10549-020-05754-8.

(67) Nadal-Serrano, M.; Morancho, B.; Escrivá-de-Romaní, S.; Bernadó Morales, C.; Luque, A.; Escorihuela, M.; Espinosa Bravo, M.; Peg, V.; Dijcks, F. A.; Dokter, W. H. A.; Cortés, J.; Saura, C.; Arribas, J. The Second Generation Antibody-Drug Conjugate SYD985 Overcomes Resistances to T-DM1. *Cancers* **2020**, *12* (3), 670. https://doi.org/10.3390/cancers12030670.

(68) Scribner, J. A.; Brown, J. G.; Son, T.; Chiechi, M.; Li, P.; Sharma, S.; Li, H.; De Costa, A.; Li, Y.; Chen, Y.; Easton, A.; Yee-Toy, N. C.; Chen, F. Z.; Gorlatov, S.; Barat, B.; Huang, L.; Wolff, C. R.; Hooley, J.; Hotaling, T. E.; Gaynutdinov, T.; Ciccarone, V.; Tamura, J.; Koenig, S.; Moore, P. A.; Bonvini, E.; Loo, D. Preclinical Development of MGC018, a Duocarmycin-Based Antibody–Drug Conjugate Targeting B7-H3 for Solid Cancer. *Mol. Cancer Ther.* **2020**, *19* (11), 2235–2244. https://doi.org/10.1158/1535-7163.MCT-20-0116.

(69) Voortman, G.; Koper, N. P. SYD985 Treatment of T-DM1 Refractory Cancer Patients. US010821191B2, November 3, 2020.

(70) Jang, S.; Powderly, J. D.; Spira, A. I.; Bakkacha, O.; Loo, D.; Bohac, G. C.; Sharma, M. Phase 1 Dose Escalation Study of MGC018, an Anti-B7-H3 Antibody-Drug Conjugate (ADC), in Patients with Advanced Solid Tumors. *J. Clin. Onc.* **2021**, *39* (15_suppl), 2631–2631. https://doi.org/10.1200/JCO.2021.39.15_suppl.2631.

(71) Ferhati, X.; Jiménez-Moreno, E.; Hoyt, E. A.; Salluce, G.; Cabeza-Cabrerizo, M.; Navo, C. D.; Compañón, I.; Akkapeddi, P.; Matos, M. J.; Salaverri, N.; Garrido, P.; Martínez, A.; Laserna, V.; Murray, T. V.; Jiménez-Osés, G.; Ravn, P.; Bernardes, G. J. L.; Corzana, F. Single Mutation on Trastuzumab Modulates the Stability of Antibody–Drug Conjugates Built Using Acetal-Based Linkers and Thiol-Maleimide Chemistry. *J. Am. Chem. Soc.* **2022**. https://doi.org/10.1021/jacs.1c07675.

(72) Hyung, S.-J.; Leipold, D. D.; Lee, D. W.; Kaur, S.; Saad, O. M. Multiplexed Quantitative Analysis of Antibody–Drug Conjugates with Labile CBI-Dimer Payloads In Vivo Using Immunoaffinity LC-MS/MS. *Anal. Chem.* **2022**, *94* (2), 1158–1168. https://doi.org/10.1021/acs.analchem.1c04338.

(x) duocarmycin reviews (23 items)

(1) Hurley, L. H.; Sun, D. (+)-CC-1065 as a Probe for Intrinsic and Protein-Induced Bending of DNA. *J. Mol. Recognit.* **1994**, *7* (2), 123–132. https://doi.org/10.1002/jmr.300070209.

(2) Boger, D. L. The Duocarmycins: Synthetic and Mechanistic Studies. *Acc. Chem. Res.* **1995**, *28* (1), 20–29. https://doi.org/10.1021/ar00049a004.

(3) Boger, D. L.; Boyce, C. W.; Garbaccio, R. M.; Goldberg, J. A. CC-1065 and the Duocarmycins: Synthetic Studies. *Chem. Rev.* **1997**, *97* (3), 787–828. https://doi.org/10.1021/cr960095g.

(4) Cacciari, B.; Romagnoli, R.; Baraldi, P. G.; Ros, T. D.; Spalluto, G. CC-1065 and the Duocarmycins: Recent Developments. *Exp. Op. Ther. Patents* **2000**, *10* (12), 1853–1871. https://doi.org/10.1517/13543776.10.12.1853.

(5) Denny, W. A. DNA Minor Groove Alkylating Agents. *Curr. Med. Chem.* **2001**, *8*, 533–544. https://doi.org/10.2174/0929867003373283.

(6) Sharma, S.; Jia, G.; Lown, J. Novel Cyclopropylindole Conjugates and Dimers: Synthesis and Anti-Cancer Evaluation. *Curr. Med. Chem.* **2001**, *1* (1), 27–45. https://doi.org/10.2174/1568011013354831.

(7) Denny, W. Nitroreductase-Based GDEPT. *Curr. Pharm. Des.* **2002**, *8* (15), 1349–1361. https://doi.org/10.2174/1381612023394584.

(8) Searcey, M. Duocarmycins - Natures Prodrugs? *Curr. Pharm. Des* **2002**, *8* (15), 1375–1389. https://doi.org/10.2174/1381612023394539.

(9) Wolkenberg, S. E.; Boger, D. L. Mechanisms of in Situ Activation for DNA-Targeting Antitumor Agents. *Chem. Rev.* **2002**, *102* (7), 2477–2496. https://doi.org/10.1021/cr010046q.

(10) Baraldi, P. G.; Bovero, A.; Fruttarolo, F.; Preti, D.; Tabrizi, M. A.; Pavani, M. G.; Romagnoli, R. DNA Minor Groove Binders as Potential Antitumor and Antimicrobial Agents. *Med. Res. Rev.* **2004**, *24* (4), 475–528. https://doi.org/10.1002/med.20000.

(11) Bando, T.; Sugiyama, H. Synthesis and Biological Properties of Sequence-Specific DNA-Alkylating Pyrrole−Imidazole Polyamides. *Acc. Chem. Res.* **2006**, *39* (12), 935–944. https://doi.org/10.1021/ar030287f.

(12) Ghosh, N.; Sheldrake, H.; Searcey, M.; Pors, K. Chemical and Biological Explorations of the Family of CC-1065 and the Duocarmycin Natural Products. *Curr. Top. Med. Chem.* **2009**, *9* (16), 1494–1524. https://doi.org/10.2174/156802609789909812.

(13) Tietze, L. F.; Krewer, B. Antibody-Directed Enzyme Prodrug Therapy: A Promising Approach for a Selective Treatment of Cancer Based on Prodrugs and Monoclonal Antibodies. *Curr. Biol. Drug Des.* **2009**, *74* (3), 205–211. https://doi.org/10.1111/j.1747-0285.2009.00856.x.

(14) Denny, W. A.; Wilson, W. R. The Design of Selectively-Activated Anti-Cancer Prodrugs for Use in Antibody-Directed and Gene-Directed Enzyme-Prodrug Therapies. *J. Pharm. Pharmacol.* **2011**, *50* (4), 387–394. https://doi.org/10.1111/j.2042-7158.1998.tb06878.x.

(15) F. Tietze, L.; Schmuck, K. Prodrugs for Targeted Tumor Therapies: Recent Developments in ADEPT, GDEPT and PMT. *Curr. Pharm. Des.* **2011**, *17* (32), 3527–3547. https://doi.org/10.2174/138161211798194459.

(16) Patil, P.; Satam, V.; Lee, M. A Short Review on the Synthetic Strategies of Duocarmycin Analogs That Are Powerful DNA Alkylating Agents. *Anti-Cancer Agents in Med. Chem.* **2015**, *15* (5), 616–630. https://doi.org/10.2174/1871520615666141216144116.

(17) Beekman, A. M.; Cominetti, M. M. D.; Searcey, M. Duocarmycins as Antibody – Drug Conjugate (ADC) Payloads. In *Drug Discovery Series No. 71*; Cytotoxic Payloads for Antibody-Drug Conjugates; The Royal Society of Chemistry, 2019; pp 187–208.

(18) Pillow, T. H.; Tercel, M. Duocarmycin-PBD Dimers as Antibody-Drug Conjugate (ADC) Payloads. In *Drug Discovery Series No. 71*; Cytotoxic Payloads for Antibody-Drug Conjugates; The Royal Society of Chemistry, 2019; pp 241–258.

(19) Procopiou, G.; O'Donnell, C. J. CXI Dimers as Antibody-Drug Conjugate (ADC) Payloads. In *Drug Discovery Series No. 71*; Cytotoxic Payloads for Antibody-Drug Conjugates; The Royal Society of Chemistry, 2019; pp 209–240.

(20) Yu, Z.; Pandian, G. N.; Hidaka, T.; Sugiyama, H. Therapeutic Gene Regulation Using Pyrrole–Imidazole Polyamides. *Adv. Drug. Del. Rev.* **2019**, *147*, 66–85. https://doi.org/10.1016/j.addr.2019.02.001.

(21) Jukes, Z.; Morais, G. R.; Loadman, P. M.; Pors, K. How Can the Potential of the Duocarmycins Be Unlocked for Cancer Therapy? *Drug Discovery Today* **2021**, *26* (2), 577–584. https://doi.org/10.1016/j.drudis.2020.11.020.

(22) Yao, H.-P.; Zhao, H.; Hudson, R.; Tong, X.-M.; Wang, M.-H. Duocarmycin-Based Antibody–Drug Conjugates as an Emerging Biotherapeutic Entity for Targeted Cancer Therapy: Pharmaceutical Strategy and Clinical Progress. *Drug Discovery Today* **2021**, *26* (8), 1857–1874. https://doi.org/10.1016/j.drudis.2021.06.012.

(23) Denny, W. A. Nitroaromatic Hypoxia-Activated Prodrugs for Cancer Therapy. *Pharmaceuticals* **2022**, *15* (2), 187. https://doi.org/10.3390/ph15020187.
