# Supplementary material for: 40 Years of Duocarmycins: A Graphical Structure/Function Review of Their Chemical Evolution, from SAR to Prodrugs and ADCs
Source: JACS Au. 2022 Nov 15;2(12):2636–44. doi: 10.1021/jacsau.2c00448 (PMC9795467; doi:10.1021/jacsau.2c00448)
Supplement: Supplementary file 2 — au2c00448_si_002.pdf [file au2c00448_si_002.pdf]

40 years of duocarmycins: a graphical structure/function review of their chemical evolution, from SAR to prodrugs and ADCs

Jan G. Felber and Oliver Thorn-Seshold; LMU Munich: https://thornseshold.cup.uni-muenchen.de

(1) SAR COMPOUNDS

Journal abbreviations

ANIE	Angewandte Chemie International Edition
BMC	Bioorganic & Medicinal Chemistry
BMCL	Bioorganic & Medicinal Chemistry Letters
EJOC	European Journal of Organic Chemistry
JACS	Journal of the American Chemical Society
JMC	Journal of Medicinal Chemistry
JOC	The Journal of Organic Chemistry
OBC	Organic & Biomolecular Chemistry

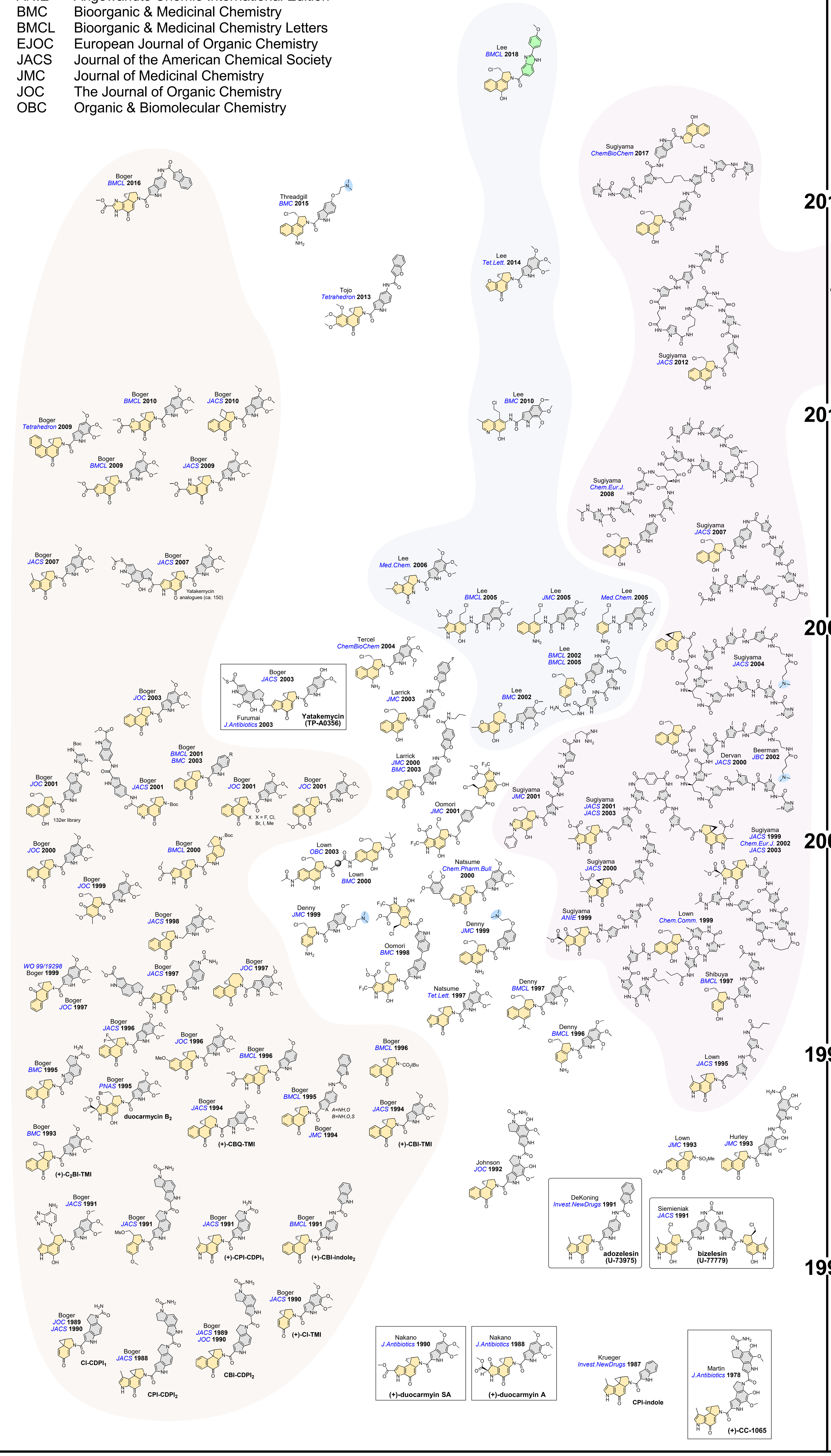

(2) PRODRUGS / BIFUNCTIONAL CONJUGATES

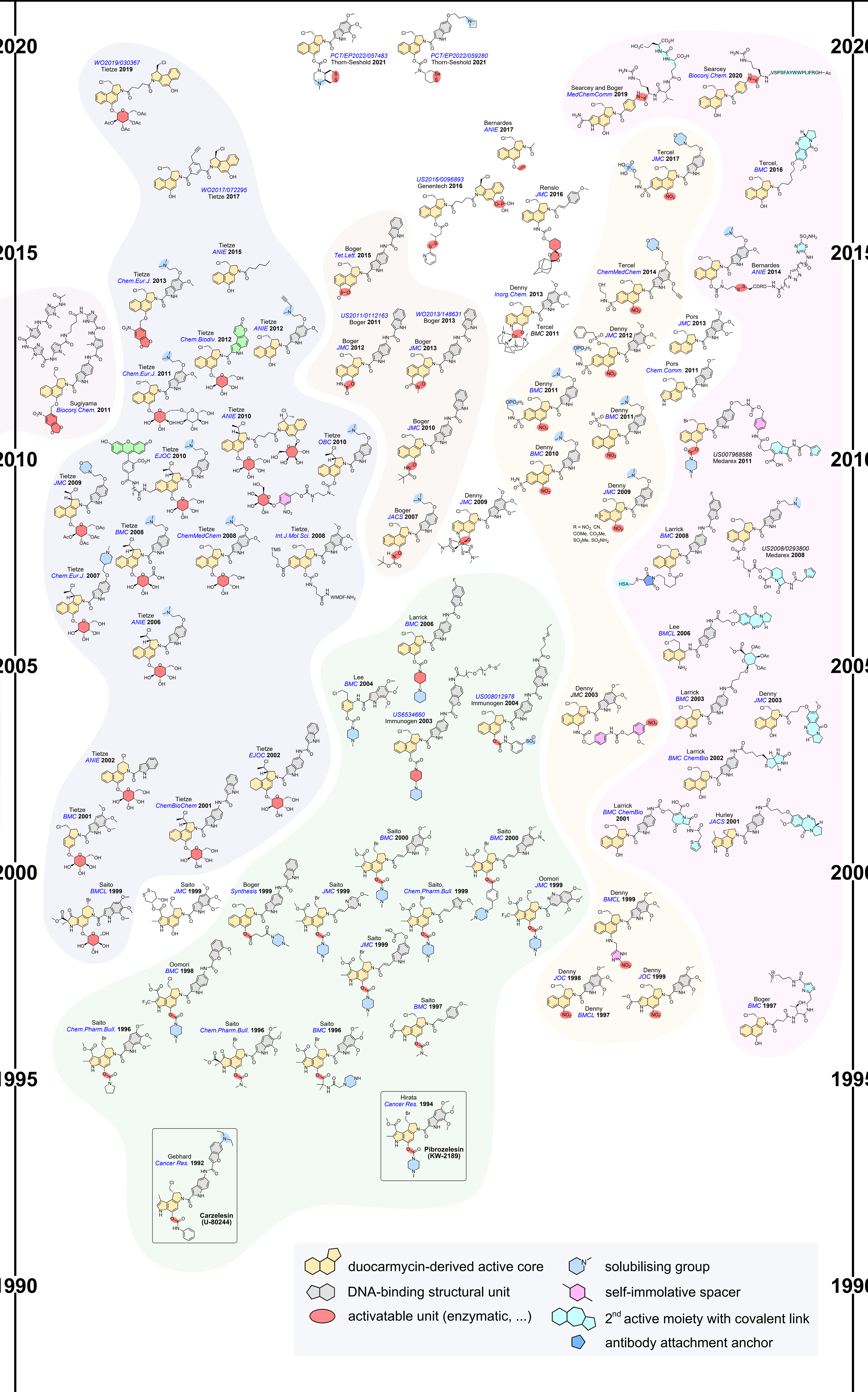

(3) ANTIBODY-DRUG CONJUGATES

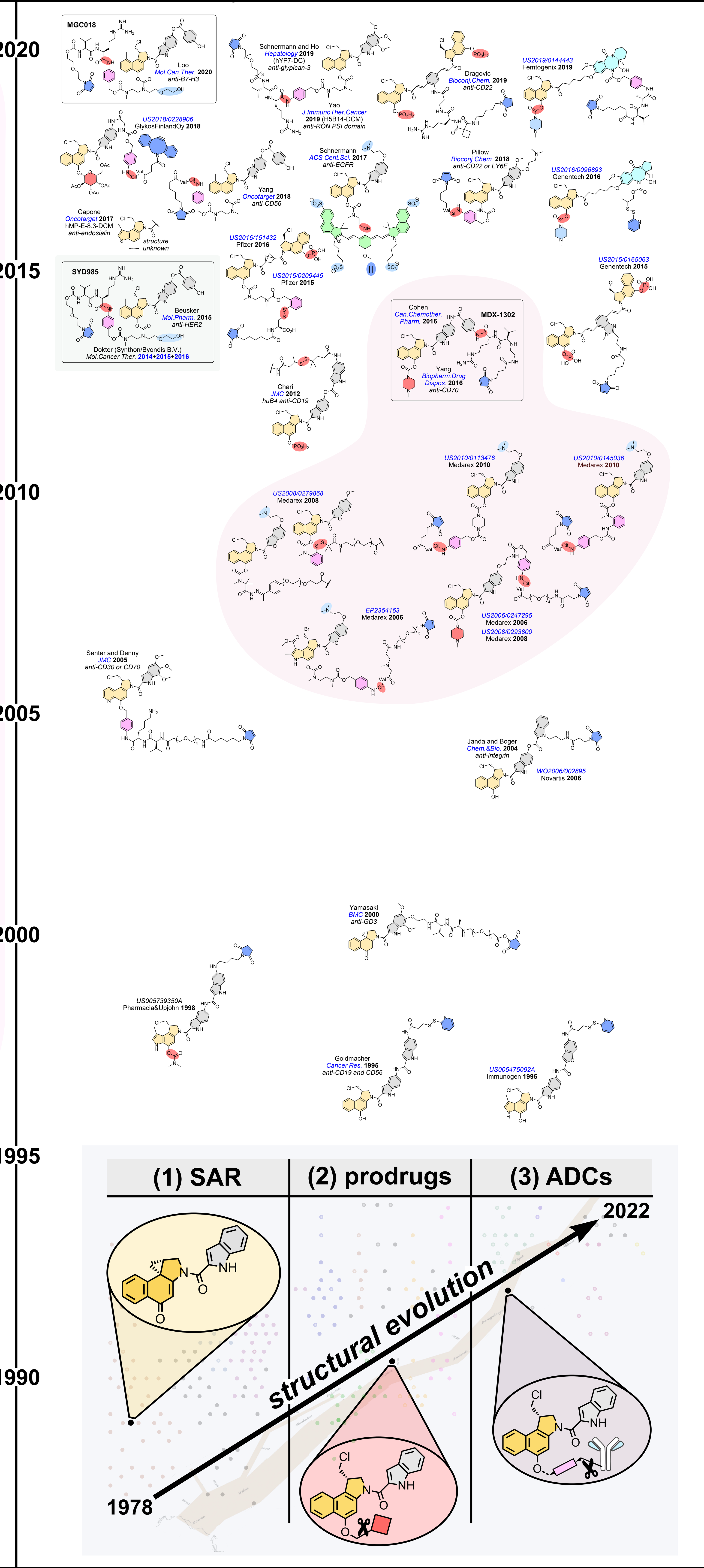

This poster accompanies a Perspective in *JACS Au* (**2022**): doi.org/10.1021/jacsau.2c00448

version 2022.09.28 - download the latest version with embedded hyperlinks at thornseshold.cup.lmu.de or tinyurl.com/200carmycins
